# Diffusion models reveal white matter microstructural changes with ageing, pathology and cognition

**DOI:** 10.1093/braincomms/fcab106

**Published:** 2021-05-19

**Authors:** Sheelakumari Raghavan, Robert I Reid, Scott A Przybelski, Timothy G Lesnick, Jonathan Graff-Radford, Christopher G Schwarz, David S Knopman, Michelle M Mielke, Mary M Machulda, Ronald C Petersen, Clifford R Jack, Prashanthi Vemuri

**Affiliations:** Department of Radiology, Mayo Clinic, Rochester, MN 55905, USA; Department of Information Technology, Mayo Clinic, Rochester, MN 55905, USA; Department of Quantitative Health Sciences, Mayo Clinic, Rochester, MN 55905, USA; Department of Quantitative Health Sciences, Mayo Clinic, Rochester, MN 55905, USA; Department of Neurology, Mayo Clinic, Rochester, MN 55905, USA; Department of Radiology, Mayo Clinic, Rochester, MN 55905, USA; Department of Neurology, Mayo Clinic, Rochester, MN 55905, USA; Department of Quantitative Health Sciences, Mayo Clinic, Rochester, MN 55905, USA; Department of Neurology, Mayo Clinic, Rochester, MN 55905, USA; Department of Psychology, Mayo Clinic, Rochester, MN 55905, USA; Department of Neurology, Mayo Clinic, Rochester, MN 55905, USA; Department of Radiology, Mayo Clinic, Rochester, MN 55905, USA; Department of Radiology, Mayo Clinic, Rochester, MN 55905, USA

**Keywords:** diffusion tensor imaging, neurite dispersion density imaging, cerebrovascular disease

## Abstract

White matter microstructure undergoes progressive changes during the lifespan, but the neurobiological underpinnings related to ageing and disease remains unclear. We used an advanced diffusion MRI, Neurite Orientation Dispersion and Density Imaging, to investigate the microstructural alterations due to demographics, common age-related pathological processes (amyloid, tau and white matter hyperintensities) and cognition. We also compared Neurite Orientation Dispersion and Density Imaging findings to the older Diffusion Tensor Imaging model-based findings. Three hundred and twenty-eight participants (264 cognitively unimpaired, 57 mild cognitive impairment and 7 dementia with a mean age of 68.3 ± 13.1 years) from the Mayo Clinic Study of Aging with multi-shell diffusion imaging, fluid attenuated inversion recovery MRI as well as amyloid and tau PET scans were included in this study. White matter tract level diffusion measures were calculated from Diffusion Tensor Imaging and Neurite Orientation Dispersion and Density Imaging. Pearson correlation and multiple linear regression analyses were performed with diffusion measures as the outcome and age, sex, education/occupation, white matter hyperintensities, amyloid and tau as predictors. Analyses were also performed with each diffusion MRI measure as a predictor of cognitive outcomes. Age and white matter hyperintensities were the strongest predictors of all white matter diffusion measures with low associations with amyloid and tau. However, neurite density decrease from Neurite Orientation Dispersion and Density Imaging was observed with amyloidosis specifically in the temporal lobes. White matter integrity (mean diffusivity and free water) in the corpus callosum showed the greatest associations with cognitive measures. All diffusion measures provided information about white matter ageing and white matter changes due to age-related pathological processes and were associated with cognition. Neurite orientation dispersion and density imaging and diffusion tensor imaging are two different diffusion models that provide distinct information about variation in white matter microstructural integrity. Neurite Orientation Dispersion and Density Imaging provides additional information about synaptic density, organization and free water content which may aid in providing mechanistic insights into disease progression.

## Graphical Abstract

**Graphical Abstract:**
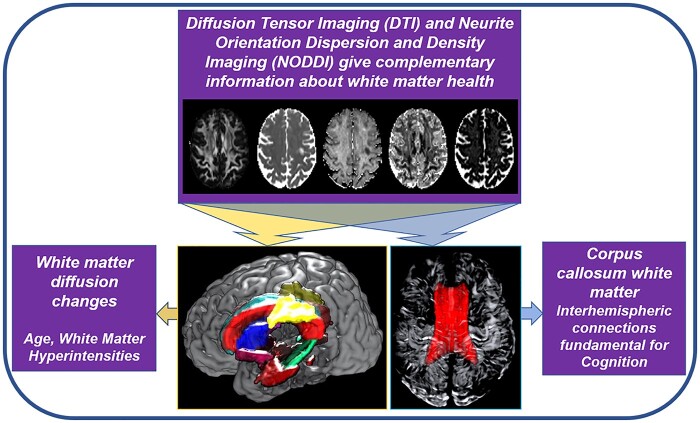

Abbreviated summary

Raghavan et al. report that advanced diffusion MRI can provide specific information about synaptic density, organization, and free water content of white matter microstructure. Age and white matter hyperintensities but not Alzheimer’s disease are key drivers of these changes. Corpus callosum white matter health is fundamental for better cognitive performance.

## Introduction

The white matter (WM) architecture of the human brain undergoes substantial changes across the life span. There is clear evidence for the association between WM changes and age as well as neuropathological processes that will lead to cognitive decline.^1–3^ Diffusion MRI is a versatile method that allows us to study these WM microstructural details. Previous findings based on diffusion tensor imaging (DTI) revealed reduced fractional anisotropy (FA) and increased mean diffusivity (MD) in association with amyloid deposition, a hallmark of Alzheimer’s disease,^4^ and cerebrovascular disease.^5^

Despite its sensitivity, the clinical utility of DTI is constrained by its inherent limitation in specificity of identifying the different diffusion environments^6^ that exist within most individual voxels. Characterizing the different water pools within a voxel with a single diffusion tensor is well known to be problematic in crossing fibre regions of WM,^7^ and also confounds the macroscopically isotropic diffusion of grey matter (GM)^8^^,^^9^ with that of CSF. The growing availability of multiband excitation allows the acquisition of roughly three times as much data in the same time as a standard DTI scan, making multiple b value (diffusion weighting) shells clinically practical. Distributing the diffusion samples over >2 b values allows the use of more sophisticated and biologically plausible models to characterize the general properties of the microstructural environments inside the axons, between them, and in the extracellular water. In addition, these models can ideally handle the ‘crossing fibre problem’ better than DTI.^7^ Neurite orientation dispersion and density imaging (NODDI) is an advanced dMRI technique that uses the additional degrees of freedom from multi-shell data to probe the microstructural complexity of neurites (dendrites and axons),^10^ separately from CSF and to a large degree also separately from each other. This biophysical modelling method divides water diffusion in the brain into three microstructural compartments: intracellular space through the Neurite Density Index (NDI), which measures the signal fraction that is due to axons and dendrites; Orientation Dispersion Index (ODI), which measures angular variation or dispersion of the neurites; and the Isotropic Volume Fraction (ISOVF), which measures free water (FW) fraction. More recently, a number of studies have demonstrated the efficiency of NODDI to provide finer granularity, in comparison to DTI metrics, to decipher the intra and extracellular microscopic features of age- and sex-specific diffusion trajectories.^11^^,^^12^ In addition, NODDI has been found to be useful for the early detection of neurodegenerative changes^13–15^ and its association with cognitive deficits.^14^^,^^16^

Recent findings have suggested that amyloid affects DTI measures^4^^,^^17^ and also the greater effect of cerebrovascular disease on diffusion alteration than Alzheimer’s disease in memory clinic patients.^17^ It is also well known that WM plays an important role in normal cognition and age-related cognitive decline.^18^^,^^19^ However, the efficiency of NODDI models over DTI models to detect Alzheimer’s disease and cerebrovascular disease pathologies, and their contribution to cognitive performance, in population-based studies remains unclear. Given the detailed quantification of biological processes by NODDI models,^6^^,^^20^^,^^21^ we hypothesized that NODDI measures would provide more sensitive features of microstructural brain changes than conventional FA and MD^10^^,^^22^ and would be more sensitive in capturing disease related processes. The overall goal of the study was to identify the relationships between demographics (age, sex and education/occupation), neuroimaging measures of Alzheimer’s disease and cerebrovascular disease, and cognition with diffusion MRI (NODDI and conventional DTI) in participants from Mayo Clinic Study of Aging (MCSA).

## Materials and methods

### Selection of participants

We identified 328 participants consisting of 264 cognitively unimpaired (CU), 57 mild cognitive impairment (MCI) and 7 dementia from the MCSA, an epidemiological cohort designed to investigate the prevalence, incidence and risk factors for MCI and dementia among the residents of Olmsted County, Minnesota. The Rochester Epidemiology Project (REP) medical records-linkage system^23^^,^^24^ was used to enumerate the MCSA sample population. The MCI and dementia participants were diagnosed based on the previously published consensus criteria.^25^ Our inclusion criteria were participants who had multi-shell diffusion data, fluid attenuated inversion recovery (FLAIR)-MRI, amyloid and tau PET scans. The clinical diagnosis was made at the time of MRI assessment and almost all clinical and imaging visits were within 60–70 days (median was 63 days with a range of 0–124 days). The amyloid negative/positive (A−/A+) and tau negative/positive (T−/T+) proportions in the sample were CU (A − T − *n* = 171, A − T + *n* = 23, A + T − *n* = 41 and A + T + *n* = 29), MCI (A − T − *n* = 16, A − T + *n* = 3, A + T − *n* = 12 and A + T + *n* = 26) and dementia (A + T − *n* = 1 and A + T + *n* = 6).

*Standard protocol approvals, registrations and patient consents*: The study was approved by the Mayo Clinic institutional review board and written informed consent was obtained from all participants or their surrogates.

### Imaging

#### MRI acquisition and processing

All participants underwent a 3 T head MRI protocol on one of two 3 T Siemens Prisma scanners running VE11 software with 64 channel receiver head coils. The protocol included a magnetization prepared rapid gradient echo (MPRAGE) sequence (TR/TE/TI = 2300/3.14/945 ms, flip angle 9°, 0.8 mm isotropic resolution), and a diffusion scan using the product VE11 Simultaneous Multi-Slice acceleration with adaptive coil combination. For the diffusion scan the field of view was 232 mm in X and Y and 162 mm in the Z direction, with 2.0 mm isotropic voxels. The echo and repetition times were 71 and 3400 ms respectively. Data consisted of 127 volumes with 13 non-diffusion-weighted images (b = 0 s/mm^2^), and 114 diffusion-encoding gradient directions (6 b = 500, 48 b = 1000 and 60 b = 2000 s/mm^2^), evenly spread over the entire spherical shells using an electrostatic repulsion model,^26^ and interspersed in time to minimize gradient heating.

**Figure 1:**
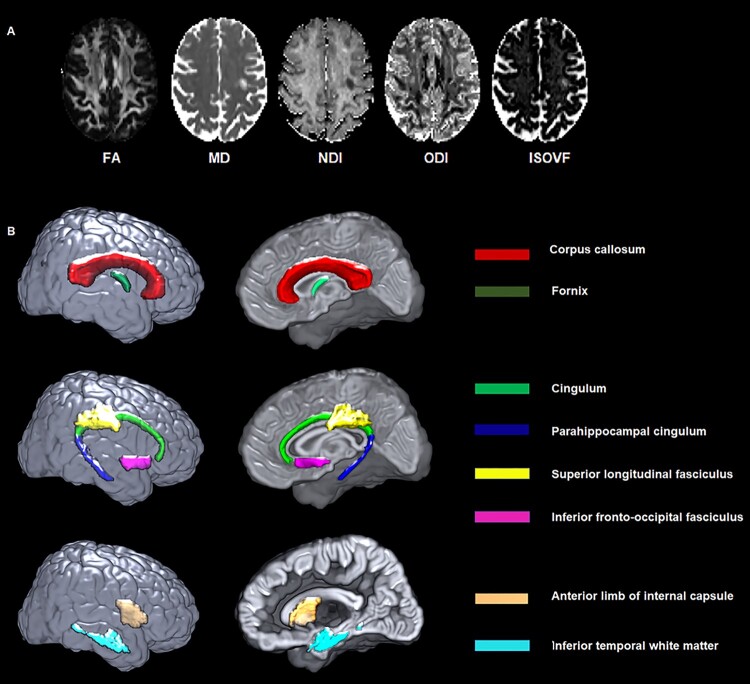
**DTI and NODDI maps from a participant and white matter tracts of interest from the JHU atlas**. (**A**) FA, MD, NDI, ODI and ISOVF maps generated from a 67-year-old cognitively unimpaired female participant. (**B**) White matter tracts of interest from JHU atlas. FA, fractional anisotropy; ISOVF, isotropic volume fraction; MD, mean diffusivity; NDI, neurite density index; ODI, orientation dispersion index.

The diffusion data were preprocessed using the in-house developed pipeline. After visual inspection, an intracranial mask was made for the diffusion MRI scan^27^ and the noise in the raw diffusion images was estimated and removed using random matrix theory.^28^ Then FSL’s eddy_cuda was used to correct for head motion and eddy current distortion,^29^ followed by the correction of Gibbs ringing^30^ and Rician bias.^31^ Diffusion tensors were fitted for both the multi-shell and extracted b = 1000 data using a non-linear least-squares fitting algorithm implemented in dipy,^32^ from which FA and MD images were generated. The NODDI model was fit by the Accelerated Microstructure Imaging via Convex Optimization (AMICO) implementation^33^ in Python. FA, MD, NDI, ODI and ISOVF maps generated from a cognitively unimpaired subject are shown in Fig. 1A.

#### Amyloid and tau assessment from PET scans

The acquisition and processing were described previously.^34^ From amyloid PET scans, a global amyloid load measure for each subject [standardized uptake value ratio (SUVR)] was computed by calculating the median uptake in the prefrontal, orbitofrontal, parietal, temporal, anterior cingulate and posterior cingulate/precuneus regions of interest (ROIs) normalized by the median amyloid PET uptake in the cerebellar crus grey matter. From tau PET scans, a composite ratio for each subject was computed by calculating median tau PET uptake in the entorhinal, amygdala, parahippocampal, fusiform, inferior temporal and middle temporal ROIs normalized by the median tau PET uptake in the cerebellar crus grey matter.

#### WMH assessment from FLAIR scans

The 3D MPRAGE and 3D T_2_-weighted FLAIR images were used to calculate WMH volume via a fully automated algorithm, updated from a previously described in-house semi-automated method.^35^ Briefly, 3D FLAIR images were preprocessed for intensity inhomogeneity correction^36^ and de-noising using a non-local means filter.^37^ Then, WMH were segmented based on location (spatial priors), intensity relative to the global distribution of GM intensity values, and intensity relative to the local neighbourhood of WM voxels. False-positive WMH segmentations were reduced by applying a white matter mask derived from the 3D MPRAGE segmentation, and by removing isolated single-voxel detections.

### Cognitive performance measures

All MCSA participants underwent a detailed neuropsychological test battery that consisted of 9 tests covering 4 cognitive subdomains.^25^^,^^38^ The present study utilized a global cognitive z-score that was derived as the *z*-transformation of the average of all nine tests across the 4 cognitive domains (memory, language, attention/executive and visuo-spatial function).^39^ Individual and compound scores from Trail Making Test (Trails) A and B (time to complete the test) were used as a sensitive test for processing speed. The raw scores were transformed into *z*-scores and averaged to create a composite score.

### Image analysis

#### ROI-based analysis

We performed an ROI analysis in ten WM tracts which were selected based on literature suggesting their association with cognition.^1^^,^^14^^,^^16^^,^^40–42^: commissural fibres: genu (GCC), body (BCC), and splenium (SCC) of corpus callosum, and fornix (FX); association fibres: cingulum (CGC), parahippocampal cingulum (CGH), superior longitudinal fasciculus (SLF), inferior fronto-occipital fasciculus; and other relevant tracts: inferior temporal WM (ITWM) and anterior limb of internal capsule (Fig. 1B). The median values of FA, MD, NDI, ODI and ISOVF were computed in these tracts by non-linearly registering an in-house modified version of the JHU ‘Eve’ WM atlas^43^ to each subject’s image using Advanced Normalization Tools–Symmetric Normalization (ANTS-SyN).^44^ In this analysis, we excluded the cuneus, precuneus, fusiform and lingual WM regions since they are too small for reliable registration. The median values of bilateral regions were then averaged, weighting by region size, to produce a single measure for each bilateral structure.

#### Voxel-based analysis of diffusion metrics

Diffusion images were analysed using an in house developed voxel-based analysis (VBA) pipeline for SPM12 in MATLAB to identify the global brain changes in association with demographics and disease pathologies. Briefly, each subjects FA, MD, NDI, ODI and ISOVF maps were non-linearly registered to a custom-made study-specific template using ANTs-SyN. To reduce partial volume effects and understand the regional results based on tissue classes, additional mask images were made using GM, WM and GM+WM masks from each subject’s segmented T_1_-weighted image. The masks were registered to the study template using an ANTS-calculated warp from the subject’s T_1_-weighted image to a T_1_-like target synthesized from the FA and MD templates. The GM, WM and GM+WM masks were thresholded to include voxels with respective tissue type fractions >0.5. Each of the normalized diffusion images was then smoothed with an 8-mm FWHM isotropic Gaussian kernel and analysed per-voxel within each tissue-class mask, using SPM12.

### Statistical analyses

Characteristics of the participants were summarized as mean (standard deviation) for the continuous variables and count (%) for the categorical variables. WMH was presented and analysed as a percentage of total intracranial volume (TIV). The distributions of WMH and amyloid were skewed, and hence log transformed to obtain a more normal distribution. To describe the relationships between NODDI and DTI parameters, we performed a series of unadjusted Pearson correlation analyses associating FA with NDI, FA with ODI, MD with NDI, MD with ODI, and MD with ISOVF across subjects within each WM tract. We also used unadjusted Pearson correlation analyses to describe associations between demographics (age, sex and education/occupation), cerebrovascular disease (WMH) biomarkers, Alzheimer’s disease (amyloid and tau) biomarkers, and ROI-based diffusion (FA, MD, NDI, ODI and ISOVF) measures (corrplot package 0.84).

To assess the contributions of cerebrovascular disease (WMH) and Alzheimer’s disease (amyloid and tau) biomarkers on the WM integrity changes, we fit multiple linear regression models with each ROI diffusion measure as the outcome variable, and with age, sex, education/occupation scores, WMH, amyloid and tau as predictor variables. All the imaging variables were standardized.

We also repeated the above analyses using voxel-wise multiple regression analyses on the smoothed DTI and NODDI images with age, sex, education/occupation, WMH, amyloid and tau as predictor variables. The generated SPM-T maps were corrected for multiple comparisons using family-wise error (FWE) with *P*_FWE_ <0.05. The voxel level analyses also helped confirm ROI level analyses and provide insights into subtle associations missed by ROI analyses.

Finally, we estimated the association of global cognition with each diffusion variable after adjusting for age, sex, education/occupation, cycle number (the number of times the cognitive battery was administered to each specific subject to adjust for practice effects), and amyloid and tau PET. We repeated the analyses for subdomain scores (memory, attention, language and visuospatial) and processing speed (Trail A, Trail B, composite score) with regional WM microstructural integrity measures. We computed partial Pearson correlations with 95% confidence intervals and report the beta coefficients from the multiple regression analyses.

### Data availability

Data used in this study are available upon reasonable request via MCSA/ADRC data sharing website.

## Results

The characteristics of the participants are summarized in Table 1. The mean (standard deviation) age was 68.3 (13.1) years, 52% were men, 30% were APOE4 carriers, 35% were amyloid positive and 27% were tau positive. Cognitively unimpaired individuals comprised 80% of this sample.

**Table 1:** Characteristics table with the mean (SD) listed for the continuous variables and count (%) for the categorical variables

	CU *n* = 264	MCI *n* = 57	Dementia *n* = 7	*P*-value
Male, *n* (%)	135 (51%)	30 (53%)	4 (57%)	0.94
Age, years	65.9 (12.8)	77.2 (9.8)	84.7 (4.2)	<0.001
APOE4 carrier, *n* (%)	63 (27%)	21 (40%)	5 (71%)	0.010
Education/occupation	12.9 (2.2)	11.9 (2.8)	12.1 (2.3)	0.013
CMC	1.5 (1.4)	2.6 (1.8)	3.0 (1.0)	<0.001
MMSE	29.0 (1.1)	26.1 (1.9)	20.3 (3.9)	<0.001
zGlobal	0.63 (0.99)	−1.80 (1.17)	−3.05 (0.49)	<0.001
zMemory	0.64 (0.96)	−1.85 (0.92)	−3.04 (0.15)	<0.001
zAttention	0.41 (1.02)	−1.50 (1.57)	−3.24 (0.78)	<0.001
zLanguage	0.35 (0.99)	−1.48 (1.38)	−2.96 (1.32)	<0.001
zVisual-spatial	0.56 (0.99)	−1.05 (1.23)	−1.87 (1.36)	<0.001
Trails A	32.0 (11.7)	51.4 (24.9)	91.5 (51.3)	<0.001
Trails B	75.6 (40.0)	163.5 (96.2)	262.3 (93.0)	<0.001
Amyloid, SUVR	1.49 (0.34)	1.95 (0.62)	3.04 (0.41)	<0.001
Amyloid positive, *n* (%)	70 (27%)	38 (67%)	7 (100%)	<0.001
Tau, SUVR	1.18 (0.11)	1.29 (0.21)	1.54 (0.30)	<0.001
Tau positive, *n* (%)	52 (20%)	29 (51%)	6 (86%)	<0.001
WMH percentage	0.66 (0.88)	1.22 (0.93)	1.98 (1.57)	<0.001

The *P*-values reported are from an ANOVA for continuous measurements and a chi-squared for categorical variables.

CU, cognitively unimpaired; CMC, cardiovascular and metabolic conditions; MCI, mild cognitive impairment; MMSE, mini mental state examination; SUVR, standardized uptake value ratio; WMH—white matter hyperintensity.

### Association between DTI and NODDI metrics in different WM tracts

**Figure 2:**
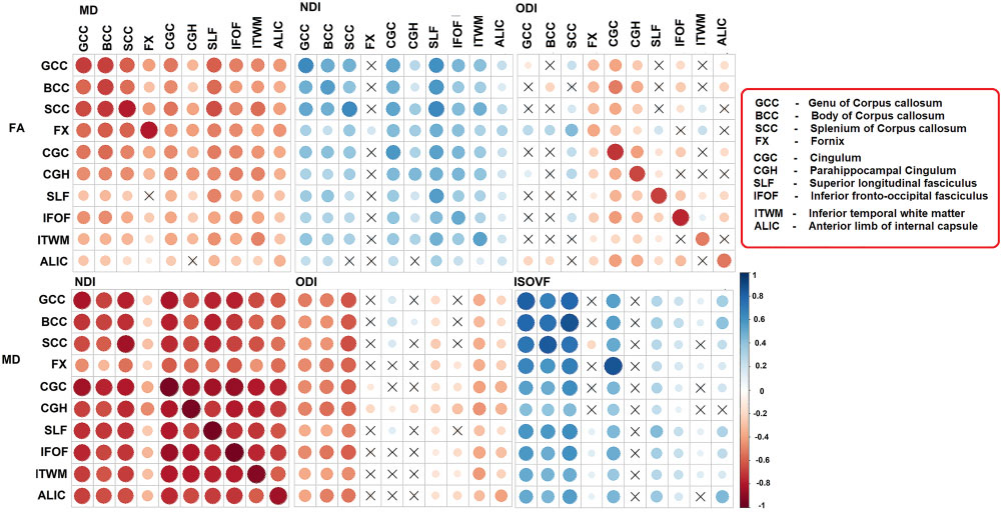
**Correlation matrix. Association between diffusion measures (FA and MD, FA and NDI, FA and ODI, MD and NDI, MD and ODI, MD and ISOVF)**. Colour legend indicates the range of correlations, the size of the circle indicates the strength of the correlation, and the symbol ‘X’ indicates the non-significant *P*-value. FA, fractional anisotropy; ISOVF, isotropic volume fraction; MD, mean diffusivity; NDI, neurite density index; ODI, orientation dispersion index.

Pearson correlations between FA, MD, NDI, ODI and ISOVF are shown in Fig. 2. Correlations within the same regions between measures: across the WM tracts, MD and NDI showed the strongest association with each other (*r* ≤ −0.676) except for in the fornix. In contrast, a modest association was observed between MD and ODI in half of the regions (fornix and association tracts). FA and NDI were associated modestly in most of the WM tracts, while FA and ODI (indicators of dispersion) had strong associations in the association tracts, anterior limb of internal capsule and inferior temporal WM (*r* ≤ −0.51). Similarly, MD was associated strongly with ISOVF in the corpus callosum.

### Associations with demographics and biomarkers of cerebrovascular disease and Alzheimer’s disease

**Figure 3:**
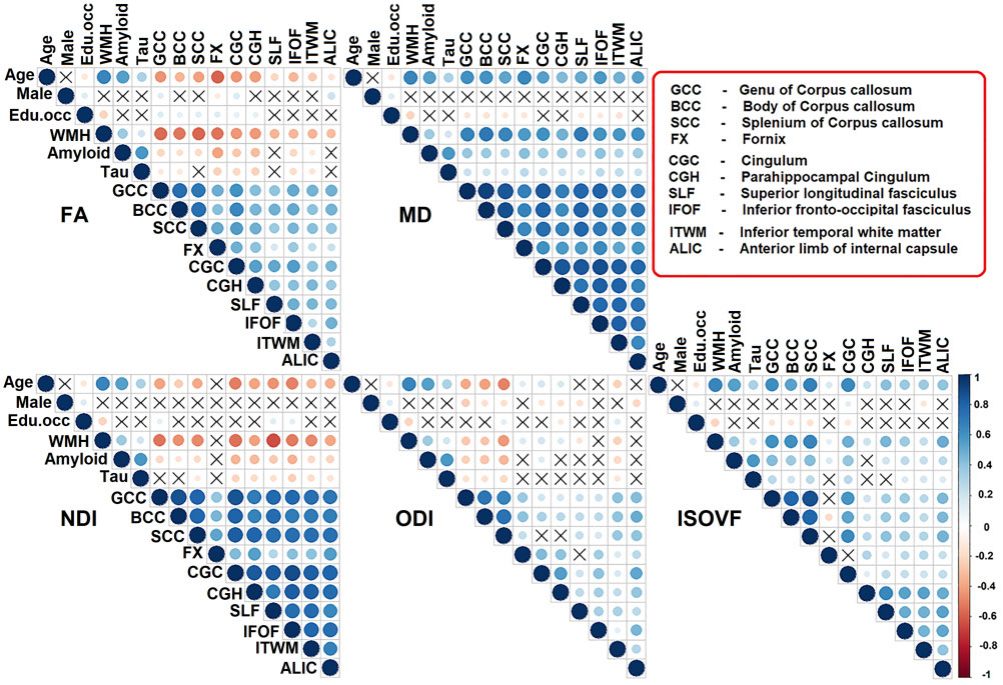
**Correlation matrix**. Association between demographics (age, sex, education/occupation) or white matter hyperintensity (WMH) or amyloid or tau and diffusion measures. Edu.occ represents education/occupation. Colour legend indicates the range of correlations, the size of the circle indicates the strength of the correlation, and the symbol “X” indicates the non-significant *P*-value. FA, fractional anisotropy; ISOVF, isotropic volume fraction; MD, mean diffusivity; NDI, neurite density index; ODI, orientation dispersion index.

Univariate associations: the univariate associations using unadjusted Pearson correlations between tract measures and age, sex, education/occupation, WMH, amyloid and tau are shown in Fig. 3. This figure highlights three broad aspects of the data: older age was significantly associated with lower FA, lower NDI, higher MD and higher ISOVF; the sex and education/occupation scores had either modest or no association with diffusion measures; and WMH showed the strongest association with all diffusion metrics in major WM tracts. We also observed associations across pairs of tracts with each DTI and NODDI measure (bottom of each triangle in Fig. 3). One can observe greater variability in the FA, ODI and ISOVF correlations across the tracts but MD and NDI appear to be correlated across all the tracts.

### Multiple regression models with focus on disease pathologies

**Figure 4:**
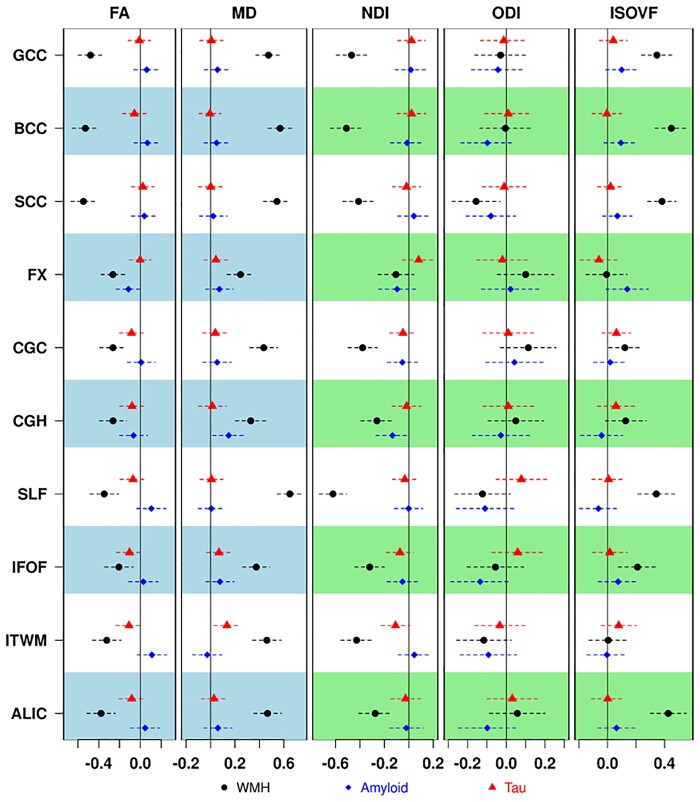
**Association of diffusion metrics with white matter hyperintensity (WMH), amyloid and tau after controlling for age, sex and education/occupation**. Different symbols below are used for each of the primary predictors. FA, fractional anisotropy; ISOVF, isotropic volume fraction; MD, mean diffusivity; NDI, neurite density index; ODI, orientation dispersion index.

The regression models with standardized disease pathologies (WMH, amyloid and tau) as predictors and standardized WM integrity measures of FA, MD, NDI, ODI and ISOVF as outcomes are shown in Fig. 4 and Supplementary Table 1. **Associations with WMH:** Across all models, WMH had the strongest associations with all dMRI metrics from all tracts. Higher WMH (a surrogate of cerebrovascular disease) was significantly associated with lower FA, higher MD, lower NDI and higher ISOVF. Splenium was the only region where WMH showed a statistically significant association with ODI.

### Associations with amyloid and tau

Higher amyloid was significantly associated with higher MD in parahippocampal cingulum (*P* = 0.026). Higher amyloid was associated with lower NDI in the same region, but the *P*-value was 0.053. In addition, higher MD was significantly associated with greater tau in inferior temporal WM (*P* = 0.014).

### Voxel level associations for confirmation of ROI analyses

**Figure 5:**
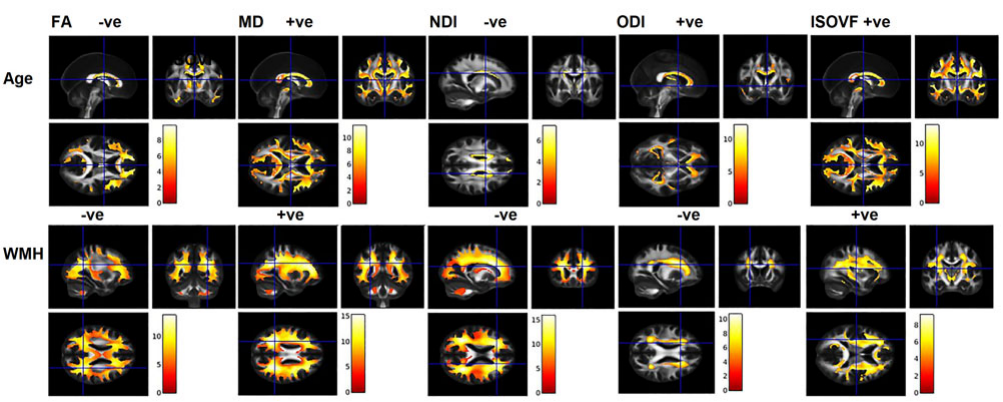
**Association between age or white matter hyper intensity (WMH) and diffusion measures**. Significance level set at *P* < 0.05, FWE corrected with an extend threshold *K* = 100. (+ve and –ve represents the kind of association between variables). FA, fractional anisotropy; ISOVF, isotropic volume fraction; MD, mean diffusivity; NDI, neurite density index; ODI, orientation dispersion index.

Similar to the ROI analysis, the voxel-wise analyses found the strongest associations for age and WMH with all diffusion metrics as displayed in Fig. 5. Modest associations were found with amyloid for both DTI and NODDI in the medial temporal lobe regions, specifically at the grey and WM junctions (Supplementary Fig. 1A). The extent and strength of tau associations with dMRI measures was minimal (Supplementary Fig. 1B).

### Association of diffusion measures with cognition

**Figure 6:**
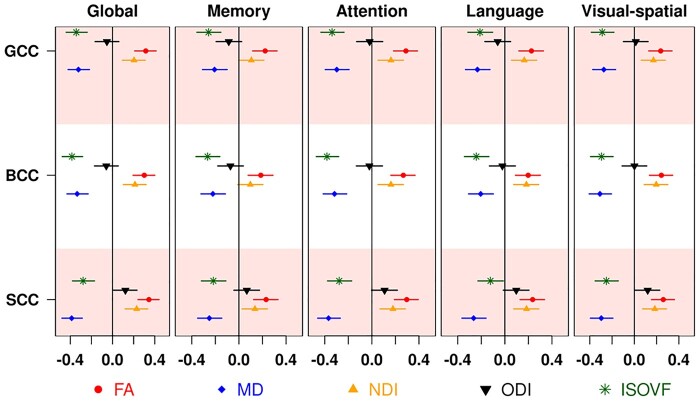
**Association of diffusion metrics with cognition after controlling for age, sex and education/occupation, cycle visit, amyloid and tau**. Different symbols below are used for each of the diffusion measures. BCC, body of corpus callosum; FA, fractional anisotropy; GCC, genu of corpus callosum; ISOVF, isotropic volume fraction; MD, mean diffusivity; NDI, neurite density index; ODI, orientation dispersion index; SCC, splenium of corpus callosum.

Association results from multiple linear regression models of the global cognition and cognitive subdomain *z*-scores with DTI and NODDI metrics after controlling for age, sex, education/occupation, cycle visit, amyloid and tau are shown in Fig. 6 for the corpus callosum tracts (where the correlations were highest). The regression coefficients for all tracts with global cognition are shown in Supplementary Table 2. Corpus callosum generally had the most significant findings except for analyses of NDI and ODI where superior longitudinal fasciculus and cingulum respectively had the greatest impact. The associations between subdomain scores and diffusion metrics had a similar pattern to that of global cognition with stronger associations with attention. Further analyses revealed significant associations between diffusion metrics and speed scores (Supplementary Fig. 2). As expected, the strongest associations were observed for corpus callosum fibres with MD and ISOVF and these changes were greatest with Trail B as an outcome.

### Association of diffusion measures with disease pathologies and cognition in non-demented participants

We also performed sensitivity analyses after excluding dementia participants. As described above, we evaluated (i) the contribution of WMH, amyloid and tau on WM changes (after adjusting for age, sex, education/occupation) and (ii) association of global cognition, subdomain and processing speed scores with corpus callosum WM measures (after adjusting for age, sex, education/occupation, and cycle number and amyloid and tau PET). There were no significant differences observed in these sensitivity analyses as shown in the supplemental material (Supplementary Figs 3–5).

## Discussion

We investigated the performance of DTI and NODDI models in capturing the microstructural brain changes associated with demographics and pathological processes and their association with cognition in 328 MCSA subjects aged 33–98 years. The major findings of the study were as follows: (i) NODDI and DTI are two different biophysical models that provide distinct information about variation in WM health. There was complementary information such that only MD and NDI had the strongest correlations with each other across the tracts; (ii) Age and WMH had the strongest associations with DTI and NODDI measures among the measured WM tracts; (iii) After adjusting for demographics, WMH was the strongest predictor of diffusion measures; (iv) Both dMRI measures were able to detect subtle Alzheimer’s disease related WM changes mainly at the medial temporal grey-white matter junctions and also WM tracts in the temporal lobes; and (v) MD and ISOVF in the corpus callosum were strongest predictors of cognitive function. Taken together, NODDI and traditional DTI measures are comparable in their predictive ability of WMH and cognition, but the non-overlapping information provided by each may aid in providing mechanistic insights into disease progression.

### Advanced biophysical models versus traditional models

An advanced biophysical model such as NODDI leverages richer multi-shell diffusion gradients to examine the physiological alterations in neurites. In Fig. 2, we directly compared variation in DTI with variation in NODDI signal. The idea is that DTI signals are sensitive to gross anatomical and neuropathological changes associated with WM,^45^ but they are inherently non-specific to disentangle the complex tissue properties of a given voxel with crossing, kissing and fanning fibres.^10^^,^^46^ On the other hand, NODDI measures demonstrated more putative cell microstructure associations across studies^10^^,^^47–51^ and have been found to be strongly correlated with neurobiological underpinnings.^7^^,^^52^^,^^53^ While decreases in NDI and increases in ISOVF are straightforward to understand, ODI changes have been hard to interpret because there is no simple physical mechanism that directly relates them to disease processes, such as demyelination, inflammation, or atrophy. Also, the direction of the correlation between axonal loss and ODI changes depends on region. Consider a hypothetical axon that runs parallel to a bundle for a few centimetres and then perpendicularly to a different bundle. The loss of the axon would increase the ODI in the parallel region and decrease it in the perpendicular region (ODI = 0 for a perfectly aligned bundle and goes to 1 where fibres spread out equally in all directions).

Unfortunately, at the macroscopic scale of a voxel the NODDI measures by themselves do not specify which axons changed. FA also suffers from this ambiguity, but unlike ODI is also directly coupled to demyelination and atrophy. Therefore, contrasting the relationship between DTI (FA and MD) and NODDI (NDI, ODI and ISOVF) can help understand the regional variations in these associations, which are largely unknown in the population.

The most consistent relationship between NODDI and DTI was seen with MD and NDI (but not with ODI and ISOVF), implicating that rather than orientation and geometry of tracts, a higher density may drive more diffusion restriction.^50^ We found a positive association of FA with NDI across various WM tracts with primarily a stronger relationship in corpus callosum fibres. This fibre pathway connects the two hemispheres, and the observed positive association between NDI and FA suggests the existence of the same underlying physiological processes (reduced axonal packing and demyelination).^10^^,^^50^ Interestingly, ODI did not show a close association with FA in the corpus callosum, which may be due to their different responses to degeneration when most of the fibres are strongly aligned.^10^^,^^54^ Specifically, if the callosal boundary retreats due to atrophy, the edge voxels will be filled in by more CSF, affecting FA but not ODI.

Outside the corpus callosum, the associations between NODDI and DTI measures were inconsistent in the association and temporal WM tracts, which may be explained by the differing sensitivity of neurites to growth/maturational trajectories.^55^^,^^56^ Across the regions, the correlation between DTI and NODDI measures was smallest in the fornix. The fornix is part of the limbic system that connects the hippocampus to the subcortical structures and is also well known for partial volume contamination by CSF. This selective weakened association indicates the correction for CSF-contamination effects in the NODDI method.

### Diffusion measures and age, sex and education/occupation

Age-associated WM changes in imaging have been widely reported. Past studies demonstrated NODDI as a key marker for studying ageing,^47^^,^^57–59^ and the association of age and sex.^1^ Consistent with the ageing literature, age was associated with a decrease in FA and NDI, increase in MD ISOVF, and increase or decrease in ODI (which depends on the tract tortuosity and the presence of crossing fibres). Among the diffusion parameters, MD had the most sensitive age effects across the majority of tracts,^1^^,^^60^ while the unconstrained diffusivity metric ISOVF demonstrated the greatest age effect in corpus callosum (genu and splenium) and cingulum. The overall widespread increase in ISOVF with age suggests the increase in FW concentration in specific brain regions. However, the key drivers of this increased FW are largely unknown. In addition to cerebrovascular disease and neurodegenerative pathologies, other possible underlying neuropathological factors include an influx of CSF or other factors like cell shrinkage,^41^ edoema,^61^ and neuroinflammation.^62^ Interestingly, past MRI and histology evidence clarified this as age- related increase in interstitial water.^63^^,^^64^

While most tracts had strong correlations with age, there were some subtle differences. With NDI, the association tracts (especially cingulum, superior longitudinal fasciculus and inferior fronto-occipital fasciculus) had the greatest age associations suggesting the presence of higher neurite density fibres in more metabolically active brain regions^65^^,^^66^ that may be vulnerable to detrimental systemic age effects. ODI exhibited heterogeneous regional variations with age. Although there is reduced tract complexity in the corpus callosum fibres, the higher dispersion in fornix, cingulum and parahippocampal cingulum suggests the greater loosening, fanning and possibly bending of axonal bundles with the advancement of age.^1^^,^^49^ As stated above, this could be explained as evidence of continuous remodelling of WM during the life span which is more evident after the sixth decade. Notably, the age-related changes in the hippocampal connections might explain the amnestic changes in the elderly population.^16^^,^^49^

The sex-specific WM integrity association of DTI and NODDI is sparsely covered in the literature. A few DTI studies evaluated sex differences and reported inconsistent findings. The inconsistency across these findings may be due to the heterogeneity of populations and differences in the analysis methods. In this study, we observed small sex differences in eight tracts, with higher FA in males and greater ODI and ISOVF in females which is consistent with previous studies in healthy adults.^1^^,^^49^^,^^67^

Reserve and resilience factors are important modulating parameters between brain injury and cognitive outcomes. Past studies showed education/occupation as a ‘proxy’ for cognitive reserve and related differences in fibre tract integrity.^68^^,^^69^ Similarly, a previous study in the MCSA population demonstrated a significant association of intellectual enrichment on FA of the genu.^70^ Although this association was modest, the present study showed a positive association of DTI and NODDI measures with corpus callosum, cingulum, parahippocampal cingulum, superior longitudinal fasciculus, inferior fronto-occipital fasciculus and inferior temporal WM, which is consistent with prior DTI studies.^70^^,^^71^ The anatomical localization provides insights into the associations between resilience mechanisms and brain maturation and plasticity.

### Diffusion measures as markers of cerebrovascular disease

Although WMH is the most commonly used biomarker for cerebrovascular disease,^2^ it only represents extensive (and structurally visible) WM damage and fails to measure the disruption or subtle changes of the underlying WM tracts. There is growing evidence supporting the utility of DTI to characterize the WM changes in cerebrovascular disease^40^^,^^72^^,^^73^ even before the appearance of WMH and cognitive decline. Notably, the observed decrease in diffusion directionality and an increase in the extent of water diffusion in conjunction with WMH are consistent with prior findings.^40^^,^^67^^,^^73^ Consistent with this idea, a recent study using FW imaging demonstrated a greater contribution of cerebrovascular disease markers than Alzheimer’s disease biomarkers (CSF and PET) in memory clinic patients.^17^

Similar to the age effect, we found a strong association of WMH with corpus callosum and association fibres.^5^ Although there is evidence for more vascular damage in the thinly myelinated anterior corpus callosum,^5^^,^^74^^,^^75^ the present study showed slight variations across the measures. Importantly, conventional DTI performed as well as NDI in detecting cerebrovascular disease changes.^76^ The decreased density and dispersion of the neurites and increased FW might contrast the lack of specificity in FA and MD to explain the underlying histological changes associated with WMH. The NDI finding in the genu of the corpus callosum was in accordance with a prior NODDI study that explored the diabetic encephalopathy in subjects with cognitive impairment.^72^ The only measure that did not show consistent associations with WMH was ODI. We believe this is due to ODI nominally being a property of only healthy neurites, and thus being more orthogonal to neuronal decay than the other NODDI measures or DTI.

Though the correlations with vascular risk were not a focus of the manuscript, as previously reported^74^ we found that WM measures from traditional DTI (FA and MD) and NODDI (NDI specifically) were significantly associated with worsening vascular risk (**Results not shown).** Recent researchers focussed on using global diffusion MRI as a cerebrovascular disease marker.^71^^,^^76^^,^^77^ However, this work sheds light on the variability in regional associations suggesting a greater sensitivity and specificity of regional markers. Future work should be undertaken to widely validate and compare diffusion outcomes as cerebrovascular disease measures.

### Diffusion measures and neuroimaging Alzheimer’s disease measures

The association between amyloid deposition and WM microstructure is still a matter of debate. A non-monotonic behaviour was found between both measures in GM^78^ and WM^79^^,^^80^ in human studies. Consistent with our region level findings, prior DTI studies reported reduced FA in corpus callosum and fornix^81^^,^^82^ in cognitively unimpaired individuals and increased axial diffusivity^83^ and accelerated FA decrease^84^ in the parahippocampal cingulum of amyloid positive individuals. As expected, the current study identified a significant global association between Aβ deposition and increased MD and ISOVF along with decreased NDI in medial temporal lobe grey-white matter junctions, which are consistent with a more recent study that reported lower neurite density in limbic and association fibres and higher medial temporal FW.^85^ The medial temporal lobe is an early region of neuronal changes in Alzheimer’s disease, so the parahippocampal cingulum findings were as expected. We also found associations between tau and non-specific MD and ISOVF association in the inferior temporal WM (Fig. 4). These results are supported by a study of tau and NODDI in a transgenic Alzheimer’s disease model.^86^ Our findings in the temporal lobe (hippocampal and parahippocampal regions) and the temporo-occipital fusiform gyrus suggest that NODDI may be able to provide more detailed information about neurite health in the presence of Alzheimer’s disease pathology.

### Diffusion measures with cognition

Association between WM DTI alterations and cognitive decline in the CU, MCI and Alzheimer’s disease populations have been reported previously.^40^^,^^67^^,^^70^^,^^75^ Although there was decreased FA and increased MD in association with cognitive decline, the exact sources of DTI signal were not studied. The present study is one of the earliest studies to compare DTI and NODDI based on their association with cognitive performance after accounting for amyloid and tau, which allows us to evaluate its utility as a cerebrovascular disease marker. As expected, both DTI and NODDI were significantly associated with global cognition and cognitive subdomain scores after adjusting for age, sex, cycle visit and Alzheimer’s disease biomarkers. The overall pattern suggests that higher coherence and density, and lower FW concentration and tract complexity, both correlate with better cognitive performance.^16^^,^^87^ Across the tracts, the strongest association of reduced WM integrity and worse global cognitive performance was observed in the corpus callosum. This is consistent with a previous DTI study in cerebrovascular disease that reported highly significant correlations of genu and splenium with global cognitive performance.^40^ Impaired interhemispheric connection pathways contribute to multiple impaired cognitive functions, such as impaired memory, psychomotor speed, frontal lobe mediated attention and executive function.^88^^,^^89^ Additionally, these observations replicated our recent study in MCI that showed greater predictability of high FA of genu on better cognitive performance,^75^ even after controlling for amyloid and tau PET.

Although there are contributions from other domains, we found that the associations with WM integrity and cognitive performance were mainly driven by attention. Importantly, our detailed investigation indicated that FW fraction in the corpus callosum predicted cognitive decline. In general, reduced neurite density correlated with worse cognitive performance with most of the tracts in all domains. Among these, the stronger association of NDI than FA in superior longitudinal fasciculus may be due to its proximity to the crossing fibres in the centrum semiovale,^90^ which corresponds to higher FA and lower tract complexity. Another speculation may be that the superior longitudinal fasciculus is connecting lateral prefrontal to parietal brain areas, which are responsible for the multifaceted processes we studied here. Notably, cingulum performed uniformly well across all domains and diffusion metrics to predict cognition. This bundle is the prominent WM tract that interconnects frontal, parietal, and medial temporal lobe and the posterior cingulate cortex. Surprisingly, the parahippocampal cingulum bundle, which connects the hippocampus to the rest of the brain areas, emerged as an important tract in visuospatial function. In contrast, deteriorations in parahippocampal cingulum have previously been implicated in association with episodic memory in older subjects^91^ and Alzheimer’s disease.^92^

Diffusion metrics are suggested to be most strongly associated with processing speed.^71^^,^^93^ Therefore, we also tested these hypotheses in the supplemental material and found that both DTI and NODDI strongly predicted processing speed (Trail B and combined). As expected, commissural fibres had the greatest effect size.

The present study has several strengths and limitations. The main strength was the extensive analyses of single and multi-shell diffusion data on WM health and cognition. Also, this is the first study to assess the relationship between NODDI metrics and cerebrovascular disease and Alzheimer’s disease biomarkers together along with associations with cognitive performance. The inclusion of a representative sample population strengthens the generalizability of the findings. Our voxel-wise and regional findings mostly corroborated each other, and the slight differences may be due to partial volume effects, smoothing, and more stringent FWE corrections. The major limitations are the cross-sectional nature of the study and the lack of histological confirmation of the observed associations. Another limitation is the smaller number of subjects in the dementia group, but the results remain the same after excluding them. Furthermore, the regularization scheme used by the AMICO implementation of NODDI acts like a prior that gives a mild preference to some values of NDI, ISOVF, and especially ODI, which could be obscuring some differences between subjects. Future longitudinal research with multiple biophysical models^11^ may provide more sensitive and conclusive findings.

In summary, the present study provides evidence of microstructural WM alterations due to ageing and age-related pathological processes, and their impact on cognition. Although NODDI-derived indices perform similar to traditional FA and MD in predicting cognitive performance, NODDI provides additional insights into the underlying synaptic density, organization and FW content which are biological processes that cannot be separated with DTI. Among DTI and NODDI indices, MD and FW fraction provided by ISOVF were the key parameters in predicting cognition. This study also highlights the spatial heterogeneity of tracts across the metrics and which highlights the importance of looking at each diffusion metrics to investigate changes in each WM region of the brain as a function of disease progression.

## Supplementary material

Supplementary material is available at *Brain Communication* online.

## Supplementary Material

fcab106_Supplementary_Data

## Abbreviations

BCC =: body of corpus callosum
CGC =: cingulum
CGH =: parahippocampal cingulum
CU =: cognitively unimpaired
DTI =: diffusion tensor imaging
FA =: fractional anisotropy
FLAIR =: fluid attenuated inversion recovery
FX =: fornix
GCC =: genu of corpus callosum
GM =: grey matter
ISOVF =: isotropic volume fraction
ITWM =: inferior temporal white matter
MCI =: mild cognitive impairment
MCSA =: Mayo Clinic Study of Aging
MD =: mean diffusivity
NDI =: neurite density index
NODDI =: neurite orientation dispersion and density Imaging
ODI =: orientation dispersion index
SCC =: splenium of corpus callosum
SLF =: superior longitudinal fasciculus
WM =: white matter
WMH =: white matter hyperintensity

## References

[1] Cox SR , Ritchie SJ, Tucker-Drob EM, et al Ageing and brain white matter structure in 3,513 UK Biobank participants. Nat Commun. 2016;7:13629. 27976682 10.1038/ncomms13629 PMC5172385

[2] Wardlaw JM , Smith EE, Biessels GJ, et al; STandards for ReportIng Vascular changes on nEuroimaging (STRIVE v1). Neuroimaging standards for research into small vessel disease and its contribution to ageing and neurodegeneration. Lancet Neurol. 2013;12(8):822-838. 23867200 10.1016/S1474-4422(13)70124-8 PMC3714437

[3] Nasrabady SE , Rizvi B, Goldman JE, Brickman AM. White matter changes in Alzheimer's disease: A focus on myelin and oligodendrocytes. Acta Neuropathol Commun. 2018;6(1):22. 29499767 10.1186/s40478-018-0515-3 PMC5834839

[4] Caballero MÁA , Song Z, Rubinski A, et al Age-dependent amyloid deposition is associated with white matter alterations in cognitively normal adults during the adult life span. Alzheimers Dement. 2020;16(4):651-661. 32147939 10.1002/alz.12062

[5] Cox SR , Lyall DM, Ritchie SJ, et al Associations between vascular risk factors and brain MRI indices in UK Biobank. Eur Heart J. Jul 21 2019;40(28):2290-2300. 30854560 10.1093/eurheartj/ehz100 PMC6642726

[6] Pines AR , Cieslak M, Larsen B, et al Leveraging multi-shell diffusion for studies of brain development in youth and young adulthood. Dev Cogn Neurosci. 2020;43:100788. 32510347 10.1016/j.dcn.2020.100788 PMC7200217

[7] Schilling KG , Janve V, Gao Y, Stepniewska I, Landman BA, Anderson AW. Histological validation of diffusion MRI fiber orientation distributions and dispersion. Neuroimage. 2018;165:200-221. 29074279 10.1016/j.neuroimage.2017.10.046 PMC5732036

[8] Jensen JH , Helpern JA, Ramani A, Lu H, Kaczynski K. Diffusional kurtosis imaging: The quantification of non-gaussian water diffusion by means of magnetic resonance imaging. Magn Reson Med. 2005;53(6):1432-1440. 15906300 10.1002/mrm.20508

[9] Jensen JH , Helpern JA. MRI quantification of non-Gaussian water diffusion by kurtosis analysis. NMR Biomed. Aug 2010;23(7):698-710. 20632416 10.1002/nbm.1518 PMC2997680

[10] Zhang H , Schneider T, Wheeler-Kingshott CA, Alexander DC. NODDI: Practical in vivo neurite orientation dispersion and density imaging of the human brain. Neuroimage. 2012;61(4):1000-16. 22484410 10.1016/j.neuroimage.2012.03.072

[11] Beck D , de Lange AG, Maximov II, et al White matter microstructure across the adult lifespan: A mixed longitudinal and cross-sectional study using advanced diffusion models and brain-age prediction. Neuroimage. 2020;224:117441. 33039618 10.1016/j.neuroimage.2020.117441

[12] Toschi N , Gisbert RA, Passamonti L, Canals S, De Santis S. Multishell diffusion imaging reveals sex-specific trajectories of early white matter degeneration in normal aging. Neurobiol Aging. 2020;86:191-200. 31902522 10.1016/j.neurobiolaging.2019.11.014

[13] Parker TD , Slattery CF, Zhang J, et al Cortical microstructure in young onset Alzheimer's disease using neurite orientation dispersion and density imaging. Hum Brain Mapp. 2018;39(7):3005-3017. 29575324 10.1002/hbm.24056 PMC6055830

[14] Slattery CF , Zhang J, Paterson RW, et al ApoE influences regional white-matter axonal density loss in Alzheimer's disease. Neurobiol Aging. 2017;57:8-17. 28578156 10.1016/j.neurobiolaging.2017.04.021 PMC5538347

[15] Vogt NM , Hunt JF, Adluru N, et al Cortical microstructural alterations in mild cognitive impairment and Alzheimer's disease dementia. Cereb Cortex (New York, NY: 1991). 2020;30(5):2948-2960. 10.1093/cercor/bhz286 PMC7197091 31833550

[16] Wen Q , Mustafi SM, Li J, et al White matter alterations in early-stage Alzheimer's disease: A tract-specific study. Alzheimers Dement (Amsterdam, Netherlands). 2019;11:576-587. 10.1016/j.dadm.2019.06.003 PMC6713788 31467968

[17] Finsterwalder S , Vlegels N, Gesierich B, et al; Utrecht VCI study group. Small vessel disease more than Alzheimer's disease determines diffusion MRI alterations in memory clinic patients. Alzheimers Dement. 2020;16(11):1504-1514. 32808747 10.1002/alz.12150 PMC8102202

[18] Bells S , Lefebvre J, Prescott SA, et al Changes in white matter microstructure impact cognition by disrupting the ability of neural assemblies to synchronize. J Neurosci. 2017;37(34):8227-8238. 28743724 10.1523/JNEUROSCI.0560-17.2017 PMC6596784

[19] Filley CM , Fields RD. White matter and cognition: Making the connection. J Neurophysiol. 2016;116(5):2093-2104. 27512019 10.1152/jn.00221.2016 PMC5102321

[20] Jelescu IO , Budde MD. Design and validation of diffusion MRI models of white matter. Front Phys. 2017;28:61. 29755979 10.3389/fphy.2017.00061 PMC5947881

[21] Novikov DS , Veraart J, Jelescu IO, Fieremans E. Rotationally-invariant mapping of scalar and orientational metrics of neuronal microstructure with diffusion MRI. Neuroimage. 2018;174:518-538. 29544816 10.1016/j.neuroimage.2018.03.006 PMC5949281

[22] Zhang YZ , Chang C, Wei XE, Fu JL, Li WB. Comparison of diffusion tensor image study in association fiber tracts among normal, amnestic mild cognitive impairment, and Alzheimer's patients. Neurology India. 2011;59(2):168-173. 21483111 10.4103/0028-3886.79129

[23] Rocca WA , Yawn BP, St Sauver JL, Grossardt BR, Melton LJ 3rd. History of the Rochester Epidemiology Project: Half a century of medical records linkage in a US population. Mayo Clinic Proc. 2012;87(12):1202-1213. 10.1016/j.mayocp.2012.08.012 PMC3541925 23199802

[24] St Sauver JL , Grossardt BR, Yawn BP, et al Data resource profile: The Rochester Epidemiology Project (REP) medical records-linkage system. Int J Epidemiol. 2012;41(6):1614-1624. 23159830 10.1093/ije/dys195 PMC3535751

[25] Petersen RC , Roberts RO, Knopman DS, et al Prevalence of mild cognitive impairment is higher in men. The Mayo Clinic Study of Aging. Neurology. 2010;75(10):889-897. 20820000 10.1212/WNL.0b013e3181f11d85 PMC2938972

[26] Caruyer E , Lenglet C, Sapiro G, Deriche R. Design of multishell sampling schemes with uniform coverage in diffusion MRI. Magn Reson Med. 2013;69(6):1534-1540. 23625329 10.1002/mrm.24736 PMC5381389

[27] Reid RI , Nedelska Z, Schwarz CG, et al Diffusion specific segmentation: Skull stripping with diffusion MRI data alone. Computational diffusion MRI mathematics and visualization. Cham: Springer; 2018.

[28] Veraart J , Novikov DS, Christiaens D, Ades-Aron B, Sijbers J, Fieremans E. Denoising of diffusion MRI using random matrix theory. Neuroimage. 2016;142:394-406. 27523449 10.1016/j.neuroimage.2016.08.016 PMC5159209

[29] Andersson JLR , Sotiropoulos SN. An integrated approach to correction for off-resonance effects and subject movement in diffusion MR imaging. Neuroimage. 2016;125:1063-1078. 26481672 10.1016/j.neuroimage.2015.10.019 PMC4692656

[30] Kellner E , Dhital B, Kiselev VG, Reisert M. Gibbs-ringing artifact removal based on local subvoxel-shifts. Magn Reson Med. 2016;76(5):1574-1581. 26745823 10.1002/mrm.26054

[31] Koay CG , Ozarslan E, Basser PJ. A signal transformational framework for breaking the noise floor and its applications in MRI. J Magn Reson (San Diego, Calif: 1997). 2009;197(2):108-119. 10.1016/j.jmr.2008.11.015 PMC2765718 19138540

[32] Garyfallidis E , Brett M, Amirbekian B, et al; Dipy Contributors. Dipy, a library for the analysis of diffusion MRI data. Front Neuroinform. 2014;8:8. 24600385 10.3389/fninf.2014.00008 PMC3931231

[33] Daducci A , Canales-Rodríguez EJ, Zhang H, Dyrby TB, Alexander DC, Thiran JP. Accelerated Microstructure Imaging via Convex Optimization (AMICO) from diffusion MRI data. Neuroimage. 2015;105:32-44. 25462697 10.1016/j.neuroimage.2014.10.026

[34] Jack CR Jr. , Wiste HJ, Weigand SD, et al Defining imaging biomarker cut points for brain aging and Alzheimer's disease. Alzheimers Dement. 2017;13(3):205-216. 27697430 10.1016/j.jalz.2016.08.005 PMC5344738

[35] Graff-Radford J , Arenaza-Urquijo EM, Knopman DS, et al White matter hyperintensities: Relationship to amyloid and tau burden. Brain. 2019;142(8):2483-2491. 31199475 10.1093/brain/awz162 PMC6658846

[36] Zhang Y , Brady M, Smith S. Segmentation of brain MR images through a hidden Markov random field model and the expectation-maximization algorithm. IEEE Trans Med Imaging. 2001;20(1):45-57. 11293691 10.1109/42.906424

[37] Manjón JV , Coupé P, Martí-Bonmatí L, Collins DL, Robles M. Adaptive non-local means denoising of MR images with spatially varying noise levels. J Magn Reson Imaging. 2010;31(1):192-203. 20027588 10.1002/jmri.22003

[38] Roberts RO , Geda YE, Knopman DS, et al The Mayo Clinic Study of Aging: Design and sampling, participation, baseline measures and sample characteristics. Neuroepidemiology. 2008;30(1):58-69. 18259084 10.1159/000115751 PMC2821441

[39] Vemuri P , Lesnick TG, Przybelski SA, et al Association of lifetime intellectual enrichment with cognitive decline in the older population. JAMA Neurol. 2014;71(8):1017-1024. 25054282 10.1001/jamaneurol.2014.963 PMC4266551

[40] Tuladhar AM , van Norden AG, de Laat KF, et al White matter integrity in small vessel disease is related to cognition. Neuroimage Clin. 2015;7:518-524. 25737960 10.1016/j.nicl.2015.02.003 PMC4338206

[41] Merluzzi AP , Dean DC 3rd, Adluru N, et al Age-dependent differences in brain tissue microstructure assessed with neurite orientation dispersion and density imaging. Neurobiol Aging. 2016;43:79-88. 27255817 10.1016/j.neurobiolaging.2016.03.026 PMC4893194

[42] Bendlin BB , Fitzgerald ME, Ries ML, et al White matter in aging and cognition: A cross-sectional study of microstructure in adults aged eighteen to eighty-three. Dev Neuropsychol. 2010;35(3):257-277. 20446132 10.1080/87565641003696775 PMC2895988

[43] Oishi K , Faria A, Jiang H, et al Atlas-based whole brain white matter analysis using large deformation diffeomorphic metric mapping: Application to normal elderly and Alzheimer's disease participants. Neuroimage. 2009;46(2):486-499. 19385016 10.1016/j.neuroimage.2009.01.002 PMC2885858

[44] Avants BB , Tustison NJ, Song G, Cook PA, Klein A, Gee JC. A reproducible evaluation of ANTs similarity metric performance in brain image registration. Neuroimage. 2011;54(3):2033-2044. 20851191 10.1016/j.neuroimage.2010.09.025 PMC3065962

[45] Alexander AL , Lee JE, Lazar M, Field AS. Diffusion tensor imaging of the brain. Neurotherapeutics. 2007;4(3):316-329. 17599699 10.1016/j.nurt.2007.05.011 PMC2041910

[46] Jeurissen B , Leemans A, Tournier JD, Jones DK, Sijbers J. Investigating the prevalence of complex fiber configurations in white matter tissue with diffusion magnetic resonance imaging. Hum Brain Mapp. 2013;34(11):2747-2766. 22611035 10.1002/hbm.22099 PMC6870534

[47] Chang YS , Owen JP, Pojman NJ, et al White matter changes of neurite density and fiber orientation dispersion during human brain maturation. PloS One. 2015;10(6):e0123656. 26115451 10.1371/journal.pone.0123656 PMC4482659

[48] Eaton-Rosen Z , Melbourne A, Orasanu E, et al Longitudinal measurement of the developing grey matter in preterm subjects using multi-modal MRI. Neuroimage. 2015;111:580-589. 25681570 10.1016/j.neuroimage.2015.02.010

[49] Kodiweera C , Alexander AL, Harezlak J, McAllister TW, Wu YC. Age effects and sex differences in human brain white matter of young to middle-aged adults: A DTI, NODDI, and q-space study. Neuroimage. 2016;128:180-192. 26724777 10.1016/j.neuroimage.2015.12.033 PMC4824064

[50] Mah A , Geeraert B, Lebel C. Detailing neuroanatomical development in late childhood and early adolescence using NODDI. PloS One. 2017;12(8):e0182340. 28817577 10.1371/journal.pone.0182340 PMC5560526

[51] Timmers I , Zhang H, Bastiani M, Jansma BM, Roebroeck A, Rubio-Gozalbo ME. White matter microstructure pathology in classic galactosemia revealed by neurite orientation dispersion and density imaging. J Inherit Metab Dis. 2015;38(2):295-304. 25344151 10.1007/s10545-014-9780-x PMC4341012

[52] Sato K , Kerever A, Kamagata K, et al Understanding microstructure of the brain by comparison of neurite orientation dispersion and density imaging (NODDI) with transparent mouse brain. Acta Radiol Open. 2017;6(4):2058460117703816. 28491462 10.1177/2058460117703816 PMC5405886

[53] Sepehrband F , Clark KA, Ullmann JF, et al Brain tissue compartment density estimated using diffusion-weighted MRI yields tissue parameters consistent with histology. Hum Brain Mapp. 2015;36(9):3687-3702. 26096639 10.1002/hbm.22872 PMC4545675

[54] Genc S , Malpas CB, Holland SK, Beare R, Silk TJ. Neurite density index is sensitive to age related differences in the developing brain. Neuroimage. 2017;148:373-380. 28087489 10.1016/j.neuroimage.2017.01.023

[55] Lebel C , Gee M, Camicioli R, Wieler M, Martin W, Beaulieu C. Diffusion tensor imaging of white matter tract evolution over the lifespan. Neuroimage. 2012;60(1):340-352. 22178809 10.1016/j.neuroimage.2011.11.094

[56] Huang H , Zhang J, Wakana S, et al White and gray matter development in human fetal, newborn and pediatric brains. Neuroimage. 2006;33(1):27-38. 16905335 10.1016/j.neuroimage.2006.06.009

[57] Billiet T , Vandenbulcke M, Mädler B, et al Age-related microstructural differences quantified using myelin water imaging and advanced diffusion MRI. Neurobiol Aging. 2015;36(6):2107-2121. 25840837 10.1016/j.neurobiolaging.2015.02.029

[58] Kunz N , Zhang H, Vasung L, et al Assessing white matter microstructure of the newborn with multi-shell diffusion MRI and biophysical compartment models. Neuroimage. 2014;96:288-299. 24680870 10.1016/j.neuroimage.2014.03.057

[59] Nazeri A , Chakravarty MM, Rotenberg DJ, et al Functional consequences of neurite orientation dispersion and density in humans across the adult lifespan. J Neurosci. 2015;35(4):1753-1762. 25632148 10.1523/JNEUROSCI.3979-14.2015 PMC4308611

[60] Slater DA , Melie-Garcia L, Preisig M, Kherif F, Lutti A, Draganski B. Evolution of white matter tract microstructure across the life span. Hum Brain Mapp. 2019;40(7):2252-2268. 30673158 10.1002/hbm.24522 PMC6865588

[61] Pasternak O , Sochen N, Gur Y, Intrator N, Assaf Y. Free water elimination and mapping from diffusion MRI. Magn Reson Med. 2009;62(3):717-730. 19623619 10.1002/mrm.22055

[62] Wang Y , Wang Q, Haldar JP, et al Quantification of increased cellularity during inflammatory demyelination. Brain. 2011;134(Pt 12):3590-3601. 22171354 10.1093/brain/awr307 PMC3235568

[63] Chad JA , Pasternak O, Salat DH, Chen JJ. Re-examining age-related differences in white matter microstructure with free-water corrected diffusion tensor imaging. Neurobiol Aging. 2018;71:161-170. 30145396 10.1016/j.neurobiolaging.2018.07.018 PMC6179151

[64] Meier-Ruge W , Ulrich J, Brühlmann M, Meier E. Age-related white matter atrophy in the human brain. Ann N Y Acad Sci. 1992;673:260-269. 1485724 10.1111/j.1749-6632.1992.tb27462.x

[65] Bartzokis G , Sultzer D, Lu PH, Nuechterlein KH, Mintz J, Cummings JL. Heterogeneous age-related breakdown of white matter structural integrity: Implications for cortical "disconnection" in aging and Alzheimer's disease. Neurobiol Aging. 2004;25(7):843-851. 15212838 10.1016/j.neurobiolaging.2003.09.005

[66] Kochunov P , Thompson PM, Lancaster JL, et al Relationship between white matter fractional anisotropy and other indices of cerebral health in normal aging: Tract-based spatial statistics study of aging. Neuroimage. 2007;35(2):478-487. 17292629 10.1016/j.neuroimage.2006.12.021

[67] Tu MC , Lo CP, Huang CF, et al Effectiveness of diffusion tensor imaging in differentiating early-stage subcortical ischemic vascular disease, Alzheimer's disease and normal ageing. PloS One. 2017;12(4):e0175143. 28388630 10.1371/journal.pone.0175143 PMC5384760

[68] Arenaza-Urquijo EM , Bosch B, Sala-Llonch R, et al Specific anatomic associations between white matter integrity and cognitive reserve in normal and cognitively impaired elders. Am J Geriatr Psychiatry. 2011;19(1):33-42. 20808130 10.1097/JGP.0b013e3181e448e1

[69] Teipel SJ , Meindl T, Wagner M, et al Longitudinal changes in fiber tract integrity in healthy aging and mild cognitive impairment: A DTI follow-up study. J Alzheimers Dis. 2010;22(2):507-522. 20847446 10.3233/JAD-2010-100234

[70] Vemuri P , Lesnick TG, Knopman DS, et al Amyloid, vascular, and resilience pathways associated with cognitive aging. Ann Neurol. 2019;86(6):866-877. 31509621 10.1002/ana.25600 PMC6899909

[71] Baykara E , Gesierich B, Adam R, et al; Alzheimer's Disease Neuroimaging Initiative. A novel imaging marker for small vessel disease based on skeletonization of white matter tracts and diffusion histograms. Ann Neurol. 2016;80(4):581-592. 27518166 10.1002/ana.24758

[72] Xiong Y , Zhang S, Shi J, Fan Y, Zhang Q, Zhu W. Application of neurite orientation dispersion and density imaging to characterize brain microstructural abnormalities in type-2 diabetics with mild cognitive impairment. J Magn Reson Imaging. 2019;50(3):889-898. 30779402 10.1002/jmri.26687

[73] Croall ID , Lohner V, Moynihan B, et al Using DTI to assess white matter microstructure in cerebral small vessel disease (SVD) in multicentre studies. Clin Sci (London, England: 1979). 2017;131(12):1361-1373. 10.1042/CS20170146 PMC5461938 28487471

[74] Vemuri P , Lesnick TG, Przybelski SA, et al Development of a cerebrovascular magnetic resonance imaging biomarker for cognitive aging. Ann Neurol. 2018;84(5):705-716. 30264411 10.1002/ana.25346 PMC6282853

[75] Raghavan S , Przybelski SA, Reid RI, et al Reduced fractional anisotropy of the genu of the corpus callosum as a cerebrovascular disease marker and predictor of longitudinal cognition in MCI. Neurobiol Aging. 2020;96:176-183. 33022474 10.1016/j.neurobiolaging.2020.09.005 PMC7722208

[76] Duering M , Finsterwalder S, Baykara E, et al Free water determines diffusion alterations and clinical status in cerebral small vessel disease. Alzheimers Dement. 2018;14(6):764-774. 29406155 10.1016/j.jalz.2017.12.007 PMC5994358

[77] Maillard P , Fletcher E, Singh B, et al Cerebral white matter free water: A sensitive biomarker of cognition and function. Neurology. 2019;92(19):e2221-e2231. 30952798 10.1212/WNL.0000000000007449 PMC6537135

[78] Montal V , Vilaplana E, Alcolea D, et al Cortical microstructural changes along the Alzheimer's disease continuum. Alzheimers Dement. 2018;14(3):340-351. 29080407 10.1016/j.jalz.2017.09.013

[79] Dong JW , Jelescu IO, Ades-Aron B, et al Diffusion MRI biomarkers of white matter microstructure vary nonmonotonically with increasing cerebral amyloid deposition. Neurobiol Aging. 2020;89:118-128. 32111392 10.1016/j.neurobiolaging.2020.01.009 PMC7314576

[80] Wolf D , Fischer FU, Scheurich A, Fellgiebel A., Alzheimer’s Disease Neuroimaging Initiative. Non-linear association between cerebral amyloid deposition and white matter microstructure in cognitively healthy older adults. J Alzheimers Dis. 2015;47(1):117-127. 26402760 10.3233/JAD-150049

[81] Gold BT , Zhu Z, Brown CA, et al White matter integrity is associated with cerebrospinal fluid markers of Alzheimer's disease in normal adults. Neurobiol Aging. 2014;35(10):2263-2271. 24866404 10.1016/j.neurobiolaging.2014.04.030 PMC4087077

[82] Chao LL , Decarli C, Kriger S, et al Associations between white matter hyperintensities and β amyloid on integrity of projection, association, and limbic fiber tracts measured with diffusion tensor MRI. PloS One. 2013;8(6):e65175. 23762308 10.1371/journal.pone.0065175 PMC3675157

[83] Molinuevo JL , Ripolles P, Simó M, et al White matter changes in preclinical Alzheimer's disease: A magnetic resonance imaging-diffusion tensor imaging study on cognitively normal older people with positive amyloid β protein 42 levels. Neurobiol Aging. 2014;35(12):2671-2680. 25002037 10.1016/j.neurobiolaging.2014.05.027

[84] Rieckmann A , Van Dijk KR, Sperling RA, Johnson KA, Buckner RL, Hedden T. Accelerated decline in white matter integrity in clinically normal individuals at risk for Alzheimer's disease. Neurobiol Aging. 2016;42:177-188. 27143434 10.1016/j.neurobiolaging.2016.03.016 PMC4857135

[85] Reas ET , Hagler DJ, Kuperman JM, et al Associations between microstructure, amyloid, and cognition in amnestic mild cognitive impairment and dementia. J Alzheimers Dis. 2020;73(1):347-357. 31796676 10.3233/JAD-190871 PMC7266036

[86] Colgan N , Siow B, O'Callaghan JM, et al Application of neurite orientation dispersion and density imaging (NODDI) to a tau pathology model of Alzheimer's disease. Neuroimage. 2016;125:739-744. 26505297 10.1016/j.neuroimage.2015.10.043 PMC4692518

[87] Fu X , Shrestha S, Sun M, et al Microstructural white matter alterations in mild cognitive impairment and Alzheimer's disease: Study based on neurite orientation dispersion and density imaging (NODDI). Clin Neuroradiol. 2020;30(3):569-579. 31175374 10.1007/s00062-019-00805-0

[88] Jokinen H , Ryberg C, Kalska H, et al LADIS group. Corpus callosum atrophy is associated with mental slowing and executive deficits in subjects with age-related white matter hyperintensities: The LADIS Study. J Neurol Neurosurg Psychiatry. 2007;78(5):491-496. 17028118 10.1136/jnnp.2006.096792 PMC2117833

[89] Román GC , Erkinjuntti T, Wallin A, Pantoni L, Chui HC. Subcortical ischaemic vascular dementia. Lancet Neurol. 2002;1(7):426-436. 12849365 10.1016/s1474-4422(02)00190-4

[90] Douaud G , Jbabdi S, Behrens TE, et al DTI measures in crossing-fibre areas: Increased diffusion anisotropy reveals early white matter alteration in MCI and mild Alzheimer's disease. Neuroimage. 2011;55(3):880-890. 21182970 10.1016/j.neuroimage.2010.12.008 PMC7116583

[91] Ezzati A , Katz MJ, Lipton ML, Zimmerman ME, Lipton RB. Hippocampal volume and cingulum bundle fractional anisotropy are independently associated with verbal memory in older adults. Brain Imaging Behav. 2016;10(3):652-659. 26424564 10.1007/s11682-015-9452-y PMC4816657

[92] Kantarci K , Murray ME, Schwarz CG, et al White-matter integrity on DTI and the pathologic staging of Alzheimer's disease. Neurobiol Aging. 2017;56:172-179. 28552181 10.1016/j.neurobiolaging.2017.04.024 PMC5523458

[93] Konieczny MJ , Dewenter A, Telgte AT, et al Multi-shell diffusion MRI models for white matter characterization in cerebral small vessel disease. Neurology. 2020;96(5):e698-e708. 33199431 10.1212/WNL.0000000000011213

