# Supplementary material for: Diffusion models reveal white matter microstructural changes with ageing, pathology and cognition
Source: Brain Commun. 2021 May 19;3(2):fcab106. doi: 10.1093/braincomms/fcab106 (PMC8202149; doi:10.1093/braincomms/fcab106)

OXFORD UNIVERSITY PRESS | Brain Communications

Diffusion models reveal white matter microstructural changes with aging, pathology, and cognition

Journal:	*Brain Communications*
Manuscript ID	BRAINCOM-2021-065
Manuscript Type:	Original Article
Date Submitted by the Author:	17-Feb-2021
Complete List of Authors:	Raghavan, Sheelakumari; Mayo Clinic Minnesota<br>Reid, Robert; Mayo Clinic, Radiology<br>Przybelski, Scott; Mayo Clinic, Biostatistics<br>Lesnick, Timothy; Mayo Clinic,<br>Graff-Radford, Jonathan; Mayo Clinic, Neurology<br>Schwarz, Christopher; Mayo Clinic, Radiology<br>Knopman, David; Mayo Clinic, Neurology<br>Mielke, Michelle; Mayo Clinic, Health Sciences Research; Mayo Clinic<br>Machulda, Mary; Mayo Clinic, Psychiatry<br>Petersen, Ronald; Mayo Clinic College of Medicine, Neurology<br>Jack, Jr., Clifford; Mayo Clinic, Department of Diagnostic Radiology;<br>Vemuri, Prashanthi; Mayo Clinic, Radiology;
Keywords:	

SCHOLARONE™
Manuscripts

Diffusion models reveal white matter microstructural changes with aging, pathology, and cognition

Sheelakumari Raghavan[1], Robert I. Reid[2], Scott A. Przybelski[3], Timothy G. Lesnick[3], Jonathan Graff-Radford[4], Christopher G. Schwarz[1], David S. Knopman[4], Michelle M. Mielke[3,4], Mary M. Machulda[5], Ronald C. Petersen[4], Clifford R. Jack Jr[1], Prashanthi Vemuri[1]

[1]Departments of Radiology, Mayo Clinic, Rochester, MN

[2]Information Technology, Mayo Clinic, Rochester, MN

[3]Health Sciences Research, Mayo Clinic, Rochester, MN

[4]Neurology, [5]Psychology, Mayo Clinic Rochester, MN

Running Title: Basis of microstructural changes

Title character count: 97

Word count: Abstract: **276**; Text: **5278**; Introduction: **553**; Discussion: **2613**

References: **100**; Figures: **6**, Color Figures: **6,** Tables: **1**, Supplementary Tables: **2**, Supplementary Figures: **2**

Disclosure: The authors report no conflicts of interest

Corresponding Author: Prashanthi Vemuri, Ph.D.

Mayo Clinic and Foundation

200 First Street SW, Rochester, MN 55905

Phone: +1 507 538 0761, Fax:+1 507 284 9778, e-mail: **vemuri.prashanthi@mayo.edu**

For Review Only

Abstract (276)

White matter microstructure undergoes progressive changes during the life-span but the neurobiological underpinnings related to aging and disease remains unclear. We used an advanced diffusion MRI, Neurite Orientation Dispersion and Density Imaging (NODDI), to investigate the microstructural alterations due to demographics, common age-related pathological processes (amyloid, tau, and white matter hyperintensities (WMH)), and cognition. We also compared NODDI findings to the older Diffusion Tensor Imaging (DTI) model based findings. 328 participants (264 cognitively unimpaired, 57 mild cognitive impairment (MCI), and 7 dementia with a mean age of 68.3 ±13.1 years) from the Mayo Clinic Study of Aging with multi-shell diffusion imaging, FLAIR-MRI as well as amyloid and tau PET scans were included in this study. White matter (WM) tract level diffusion measures were calculated from DTI and NODDI. Pearson correlation and multiple linear regression analyses were performed with diffusion measures as the outcome and age, sex, education/occupation, WMH, amyloid, and tau as predictors. Analyses were also performed with each dMRI measure as a predictor of cognitive outcomes. Age and WMH were the strongest predictors of all WM diffusion measures with low associations with amyloid and tau. However, neurite density decrease from NODDI was observed with amyloidosis specifically in the temporal lobes. WM integrity (mean diffusivity and free water) in the corpus callosum showed the greatest associations with cognitive measures. All diffusion measures provided information about WM aging and WM disease processes and were associated with cognition. NODDI and DTI are two different biophysical models that provide distinct information about variation in WM microstructural integrity. NODDI provides additional information about synaptic density, organization, and free water content which may aid in providing mechanistic insights into disease progression.

Keywords: Diffusion tensor imaging; neurite dispersion density imaging; cerebrovascular disease

For Review Only

Introduction

The white matter (WM) architecture of the human brain undergoes substantial changes across the life span. There is clear evidence for the association between WM changes and age as well as neuropathological processes that will lead to cognitive decline.[1-3] Diffusion MRI is a versatile method that allows us to study these WM microstructural details. Previous findings based on Diffusion Tensor Imaging (DTI) revealed reduced fractional anisotropy (FA) and increased mean diffusivity (MD) in association with amyloid deposition, a hallmark of Alzheimer's disease (AD)[4], and cerebrovascular disease (CVD).[5]

Despite its sensitivity, the clinical utility of DTI is constrained by its inherent limitation in specificity of identifying the different diffusion environments[6] that exist within most individual voxels. Characterizing the different water pools within a voxel with a single diffusion tensor is well known to be problematic in crossing fiber regions of WM[7], and also confounds the macroscopically isotropic diffusion of gray matter (GM)[8,9] with that of CSF. The growing availability of multiband excitation allows the acquisition of roughly 3 times as much data in the same time as a standard DTI scan, making multiple b value (diffusion weighting) shells clinically practical. Distributing the diffusion samples over > 2 b values allows the use of more sophisticated and biologically plausible models to characterize the general properties of the microstructural environments inside the axons, between them, and in the extracellular water. In addition, these models can ideally handle the "crossing fiber problem" better than DTI.[7] Neurite orientation dispersion and density imaging (NODDI) is an advanced dMRI technique that uses the additional degrees of freedom from multi-shell data to probe the microstructural complexity of neurites (dendrites and axons)[10], separately from CSF and to a large degree also separately from each other. This biophysical modelling method divides water diffusion in the brain into three microstructural compartments: intracellular space through the Neurite Density Index (NDI), which measures the signal fraction that is due to axons and dendrites; Orientation Dispersion Index (ODI), which measures angular variation or dispersion of the neurites; and the Isotropic Volume Fraction (ISOVF), which measures free water (FW) fraction. More recently, a number of studies have demonstrated the efficiency of NODDI to provide finer granularity, in comparison to DTI metrics, to decipher the intra and extracellular microscopic features of age-

For Review Only

and sex-specific diffusion trajectories.[11,12] In addition, NODDI has been found to be useful for the early detection of neurodegenerative changes[13-15] and its association with cognitive deficits.[14,16]

Recent findings have suggested that amyloid affects DTI measures[4,17] and also the greater effect of CVD on diffusion alteration than AD in memory clinic patients.[17] It is also well known that WM plays an important role in normal cognition and age-related cognitive decline.[18,19] However, the efficiency of NODDI models over DTI models to detect AD and CVD pathologies, and their contribution to cognitive performance, in population based studies remains unclear. Given the detailed quantification of biological processes by NODDI models,[6,20,21] we hypothesized that NODDI measures would provide more sensitive features of microstructural brain changes than conventional FA and MD[10,22] and would be more sensitive in capturing disease related processes. The overall goal of the study was to identify the relationships between demographics (age, sex, and education/occupation), neuroimaging measures of AD and CVD, and cognition with diffusion MRI (NODDI and conventional DTI) in participants from Mayo Clinic Study of Aging (MCSA).

For Review Only

Materials and methods

Selection of Participants

We identified 328 participants consisting of 264 cognitively unimpaired, 57 mild cognitive impairment (MCI), and 7 dementia from the MCSA, an epidemiological cohort designed to investigate the prevalence, incidence, and risk factors for MCI and dementia among the residents of Olmsted County, Minnesota. The Rochester Epidemiology Project (REP) medical records-linkage system[23, 24] was used to enumerate the MCSA sample population. The MCI and dementia participants were diagnosed based on the previously published consensus criteria.[25] Our inclusion criteria was participants who had multi-shell diffusion data, FLAIR-MRI, amyloid, and tau PET scans.

Standard protocol approvals, registrations, and patient consents: The study was approved by the Mayo Clinic institutional review board and written informed consent was obtained from all participants or their surrogates.

Imaging

MRI acquisition and processing: All participants underwent a 3T head MRI protocol on one of two 3T Siemens Prisma scanners running VE11 software with 64 channel receiver head coils. The protocol included a magnetization prepared rapid gradient echo (MPRAGE) sequence (TR/TE/TI = 2300/3.14/945 ms, flip angle 9°, 0.8 mm isotropic resolution), and a diffusion scan using the product VE11 Simultaneous Multi-Slice acceleration with adaptive coil combination. For the diffusion scan the field of view was 232 mm in X and Y and 162 mm in the Z direction, with 2.0 mm isotropic voxels. The echo and repetition times were 71 and 3400 ms respectively. Data consisted of 127 volumes with 13 non diffusion-weighted images (b=0 s/mm^2), and 114 diffusion-encoding gradient directions (6 b = 500, 48 b = 1000, and 60 b = 2000 s/mm^2), evenly spread over the entire spherical shells using an electrostatic repulsion model[26], and interspersed in time to minimize gradient heating.

The diffusion data were preprocessed using the in-house developed pipeline. After visual inspection, an intracranial mask was made for the diffusion MRI scan[27] and the noise in the raw diffusion images was estimated and removed using random matrix theory.[28] Then FSL's eddy_cuda was used to correct for head motion and eddy current distortion,[29] followed by the correction of Gibbs ringing[30] and Rician bias.[31] Diffusion tensors were fitted for both the multi-shell and extracted b=1000 data using a nonlinear least-squares fitting algorithm implemented in dipy,[32] from which FA and MD images were generated. The NODDI model was fit by the Accelerated Microstructure Imaging via Convex Optimization (AMICO) implementation[33] in Python. FA, MD, NDI, ODI and ISOVF maps generated from a representative subject are shown in **Fig. 1A.**

Amyloid and Tau Assessment from PET scans: The acquisition and processing were described previously[34]. From amyloid PET scans, a global amyloid load measure for each subject (standardized uptake value ratio (SUVR)) was computed by calculating the median uptake in the prefrontal, orbitofrontal, parietal, temporal, anterior cingulate, and posterior cingulate/precuneus ROI's normalized by the median amyloid PET uptake in the cerebellar crus grey matter. From tau PET scans, a composite ratio for each subject was computed by calculating median tau PET uptake in the entorhinal, amygdala, parahippocampal, fusiform, inferior temporal, and middle temporal ROI's normalized by the median tau PET uptake in the cerebellar crus grey matter.

For Review Only

WMH assessment from FLAIR scans: The 3D MPRAGE and 3D T2-weighted FLAIR images were used to calculate WMH volume via a fully-automated algorithm, updated from a previously described in-house semi-automated method.[35] Briefly, 3D FLAIR images were preprocessed for intensity inhomogeneity correction[36] and de-noising using a non-local means filter.[37] Then, WMH were segmented based on location (spatial priors), intensity relative to the global distribution of GM intensity values, and intensity relative to the local neighborhood of WM voxels. False-positive WMH segmentations were reduced by applying a white matter mask derived from the 3D MPRAGE segmentation, and by removing isolated single-voxel detections.

Cognitive Performance Measures

For Review Only

All MCSA participants underwent a detailed neuropsychological test battery that consisted of 9 tests covering 4 cognitive subdomains.[25,38] The present study utilized a global cognitive z-score that was derived as the z-transformation of the average of all nine tests across the 4 cognitive domains (memory, language, attention/executive, and visuo-spatial function).[39] Individual and compound scores from Trail Making Test (Trails) A and B (time to complete the test) were used as a sensitive test for processing speed. The raw scores were transformed into z-scores and averaged to create a composite score.

Image Analysis

Region of Interest (ROI) based Analysis

We performed an ROI analysis in ten WM regions: commissural fibers: genu (GCC), body (BCC), and splenium (SCC) of corpus callosum, and fornix (FX); association fibers: cingulum (CGC), parahippocampal cingulum (CGH), superior longitudinal fasciculus (SLF), inferior fronto-occipital fasciculus (IFOF); and other relevant tracts: inferior temporal WM (ITWM) and anterior limb of internal capsule (ALIC) (**Fig. 1B**). We selected these tracts based on literature suggesting their association with cognition.[1,14,16,40-42] The median values of FA, MD, NDI, ODI and ISOVF were computed in these tracts by non-linearly registering an in-house modified version of the JHU "Eve" WM atlas[43] to each subject's image using Advanced Normalization Tools –Symmetric Normalization (ANTS-SyN).[44] In this analysis, we excluded the cuneus, precuneus, fusiform, and lingual WM regions since they are too small for reliable registration. The median values of bilateral regions were then averaged, weighting by region size, to produce a single measure for each bilateral structure.

Voxel Based Analysis of diffusion metrics

Diffusion images were analyzed using an in house developed voxel-based analysis (VBA) pipeline for SPM12 in MATLAB to identify the global brain changes in association with demographics and disease pathologies. Briefly, each subjects FA, MD, NDI, ODI and ISOVF maps were nonlinearly registered to a custom-made study-specific template using ANTs-SyN. In order to reduce partial volume effects and understand the regional results on the basis of tissue class, additional mask images were made using GM, WM, and GM+WM masks from each subject's segmented T1 weighted image. The masks were registered to the study template using an ANTS-calculated warp from the subject's T1 weighted image to a T1-like target synthesized from the FA and MD templates. The GM, WM, and GM+WM masks were thresholded to include voxels with respective tissue type fractions > 0.5. Each of the normalized diffusion images was then smoothed with an 8-mm FWHM isotropic Gaussian kernel and analyzed per-voxel within each tissue-class mask, using SPM12.[45]

Statistical analyses

Characteristics of the participants were summarized as mean (standard deviation) for the continuous variables and count (%) for the categorical variables. WMH was presented and analyzed as a percentage of total intracranial volume (TIV). The distributions of WMH and amyloid were skewed, and hence log transformed to obtain a more normal distribution. To describe the relationships between NODDI and DTI parameters, we performed a series of unadjusted Pearson correlation analyses associating FA with NDI, FA with ODI, MD with NDI, MD with ODI, and MD with ISOVF across subjects within each WM tract. We also used unadjusted Pearson correlation analyses to describe associations between demographics (age, sex, and education/occupation), CVD (WMH) biomarkers, AD (amyloid and tau) biomarkers, and ROI based diffusion (FA, MD, NDI, ODI and ISOVF) measures (corrplot package 0.84).

To assess the contributions of CVD (WMH) and AD (amyloid and tau) biomarkers on the WM integrity changes, we fit multiple linear regression models with each ROI diffusion measure as the outcome variable, and with age, sex, education/occupation scores, WMH, amyloid and tau as predictor variables. All of the imaging variables were standardized.

We also repeated the above analyses using voxel-wise multiple regression analyses on the smoothed DTI and NODDI images with age, sex, education/occupation, WMH, amyloid and tau as predictor variables. The generated SPM-T maps were corrected for multiple comparisons

For Review Only

using family-wise error (FWE) with $P_{FWE}<0.05$. The voxel level analyses also helped confirm ROI level analyses and also provide insights into subtle associations missed by ROI analyses.

Finally, we estimated the association of global cognition with each diffusion variable after adjusting for age, sex, education/occupation, cycle number (the number of times the cognitive battery was administered to each specific subject to adjust for practice effects), and amyloid and tau PET. We repeated the analyses for subdomain scores (memory, attention, language and visuospatial) and processing speed (Trail A, Trail B, composite score) with regional WM microstructural integrity measures. We computed partial Pearson correlations with 95% confidence intervals and report the beta coefficients from the multiple regression analyses.

Results

The characteristics of the participants are summarized in **Table 1**. The mean (standard deviation) age was 68.3 (13.1) years, 52% were men, 30% were APOE4 carriers, 35% were amyloid positive, and 27% were tau positive. Cognitively unimpaired individuals comprised 80% of this sample.

Association between DTI and NODDI metrics in different WM tracts

Pearson correlations between FA, MD, NDI, ODI, and ISOVF are shown in **Fig. 2**. **Correlations within the same regions between measures:** Across the WM tracts, MD and NDI showed the strongest association with each other ($r \leq -0.676$) except for in the fornix. In contrast, a modest association was observed between MD and ODI in half of the regions (fornix and association tracts). FA and NDI were associated modestly in most of the WM tracts, while FA and ODI(indicators of dispersion) had strong associations in the association tracts, anterior limb of internal capsule, and inferior temporal WM ($r \leq -0.51$). Similarly, MD was associated strongly with ISOVF in the corpus callosum.

Associations with demographics and biomarkers of CVD and AD

Univariate Associations: The univariate associations using unadjusted Pearson correlations between tract measures and age, sex, education/occupation, WMH, amyloid, and tau are shown in **Fig. 3**. This figure highlights three broad aspects of the data: older age was significantly associated with lower FA, lower NDI, higher MD and higher ISOVF; the sex and education/occupation scores had either modest or no association with diffusion measures; and WMH showed the strongest association with all diffusion metrics in major WM tracts. We also observed associations across pairs of tracts with each DTI and NODDI measure (bottom of each

For Review Only

triangle in **Fig. 3**). One can observe greater variability in the FA, ODI, and ISOVF correlations across the tracts but MD and NDI appear to be correlated across all the tracts.

Multiple regression models with focus on disease pathologies: The regression models with standardized disease pathologies (WMH, amyloid, and tau) as predictors and standardized WM integrity measures of FA, MD, NDI, ODI and ISOVF as outcomes are shown in **Fig. 4** and **Supplementary Table 1**. **Associations with WMH:** Across all models, WMH had the strongest associations with all dMRI metrics from all tracts. Higher WMH (a surrogate of CVD) was significantly associated with lower FA, higher MD, lower NDI, and higher ISOVF. Splenium was the only region where WMH showed a statistically significant association with ODI.

Associations with amyloid and tau: Higher amyloid was significantly associated with higher MD in parahippocampal cingulum ($p = 0.026$). Higher amyloid was associated with lower NDI in the same region but the p-value was 0.053. In addition, higher MD was significantly associated with greater tau in inferior temporal WM ($p = 0.014$).

For Review Only

Voxel level associations for confirmation of ROI analyses

Similar to the ROI analysis, the voxel-wise analysis found the strongest associations for age and WMH with all diffusion metrics as displayed in **Fig. 5.** Modest associations were found with amyloid for both DTI and NODDI in the medial temporal lobe regions, specifically at the grey and WM junctions (**Supplementary Fig. 1A**). The extent and strength of tau associations with dMRI measures was minimal (**Supplemental Fig. 1B**).

Association of diffusion measures with Cognition

Association results from multiple linear regression models of the global cognition and cognitive subdomain z-scores with DTI and NOODDI metrics after controlling for age, sex, education/occupation, cycle visit, amyloid, and tau are shown in **Fig. 6** for the corpus callosum tracts (where the correlations were highest). The regression coefficients for all tracts with global cognition are shown in **Supplementary Table 2**. Corpus callosum generally had the most significant findings except for NDI and ODI, in which superior longitudinal fasciculus and cingulum respectively had the greatest impact. The associations between subdomain scores and diffusion metrics had a similar pattern to that of global cognition with the stronger associations primarily with attention. Further analyses revealed significant associations between diffusion metrics and speed scores **(Supplementary Fig. 2)**. As expected, the strongest associations were

observed for corpus callosum fibers with MD and ISOVF and Trail B having the strongest association.

Discussion

We investigated the performance of DTI and NODDI models in capturing the microstructural brain changes associated with demographics and pathological processes and their association with cognition in 328 MCSA subjects aged 33-98 years. The major findings of the study were: (1) NODDI and DTI are two different biophysical models that provide distinct information about variation in WM health. There was complementary information such that –only MD and NDI had the strongest correlations with each other across the tracts; (2) Age and WMH had the strongest associations with DTI and NODDI measures among the measured WM tracts; (3) After adjusting for demographics, WMH was the strongest predictor of diffusion measures; (4) Both dMRI measures were able to detect subtle AD related WM changes mainly at the medial temporal grey-white matter junctions and also WM tracts in the temporal lobes; (5) MD and ISOVF in the corpus callosum were strongest predictors of cognitive function. Taken together, NODDI and traditional DTI measures are comparable in their predictive ability of WMH and cognition but the non-overlapping information provided by each may aid in providing mechanistic insights into disease progression.

Advanced biophysical models versus traditional models

An advanced biophysical model such as NODDI leverages richer multi-shell diffusion gradients to examine the physiological alterations in neurites. In **Fig. 2**, we directly compared variation in DTI with variation in NODDI signal. The idea is that DTI signals are sensitive to gross anatomical and neuropathological changes associated with WM,[46] but they are inherently non-specific to disentangle the complex tissue properties of a given voxel with crossing, kissing and fanning fibers.[10,47] On the other hand, NODDI measures demonstrated more putative cell microstructure associations across studies[10,48-52] and have been found to be strongly correlated with neurobiological underpinnings.[7,53,54] While decreases in NDI and increases in ISOVF are straightforward to understand, ODI changes have been hard to interpret because there is no simple physical mechanism that directly relates them to disease processes such as demyelination, inflammation, or atrophy. Also, the direction of the correlation between axonal loss and ODI changes depends on region. Consider a hypothetical axon that runs parallel to a bundle for a few

For Review Only

centimeters and then perpendicularly to a different bundle. The loss of the axon would increase the ODI in the parallel region, and decrease it in the perpendicular region (ODI = 0 for a perfectly aligned bundle and goes to 1 where fibers spread out equally in all directions).

Unfortunately at the macroscopic scale of a voxel the NODDI measures by themselves do not specify which axons changed. FA also suffers from this ambiguity, but unlike ODI is also directly coupled to demyelination and atrophy. Therefore, contrasting the relationship between DTI (FA and MD) and NODDI (NDI, ODI, and ISOVF) can help in understanding the regional variations in these associations, which are largely unknown in the population.

The most consistent relationship between NODDI and DTI was seen with MD and NDI (but not with ODI and ISOVF), implicating that rather than orientation and geometry of tracts, a higher density may drive more diffusion restriction.[51,55] We found a positive association of FA with NDI across various WM tracts with primarily a stronger relationship in corpus callosum fibers. This fiber pathway connects the two hemispheres, and the observed positive association between NDI and FA suggests the existence of the same underlying physiological processes (reduced axonal packing and demyelination).[10,51] Interestingly, ODI did not show a close association with FA in the corpus callosum, which may be due to their different responses to degeneration when most of the fibers are strongly aligned.[10,56] Specifically, if the callosal boundary retreats due to atrophy, the edge voxels will be filled in by more CSF, affecting FA but not ODI.

Outside the corpus callosum, the associations between NODDI and DTI measures were inconsistent in the association and temporal WM tracts, which may be explained by the differing sensitivity of neurites to growth/maturational trajectories.[57,58] Across the regions, the correlation between DTI and NODDI measures were smallest in the fornix. The fornix is part of the limbic system that connects the hippocampus to the subcortical structures and is also well known for partial volume contamination by CSF. This selective weakened association indicates the correction for CSF-contamination effects in the NODDI method.

Diffusion measures and age, sex and education/occupation

Age-associated WM changes in imaging have been widely reported. Past studies demonstrated NODDI as a key marker for studying aging,[48,59-61] and the association of age and sex.[1] Consistent with the aging literature, age was associated with a decrease in FA and NDI, increase in MD ISOVF, and increase or decrease in ODI (which depends on the tract tortuosity and the presence

For Review Only

of crossing fibers). Among the diffusion parameters, MD had the most sensitive age effects across the majority of tracts,[1,62] while the unconstrained diffusivity metric ISOVF demonstrated the greatest age effect in corpus callosum (genu and splenium) and cingulum. The overall widespread increase in ISOVF with age suggests the increase in FW concentration in specific brain regions. However, the key drivers of this increased FW are largely unknown. In addition to CVD and neurodegenerative pathologies, other possible underlying neuropathological factors include an influx of CSF or other factors like cell shrinkage,[41] edema,[63] and neuroinflammation.[64] Interestingly, past MRI and histology evidence clarified this as age- related increase in interstitial water. [65,66]

While most tracts had strong correlations with age, there were some subtle differences. With NDI, the association tracts (especially cingulum, superior longitudinal fasciculus, and inferior fronto-occipital fasciculus) had the greatest age associations suggesting the presence of higher neurite density fibers in more metabolically active brain regions[67,68] that may be vulnerable to detrimental systemic age effects. ODI exhibited heterogeneous regional variations with age. Although there is reduced tract complexity in the corpus callosum fibers, the higher dispersion in fornix, cingulum, and parahippocampal cingulum suggests the greater loosening, fanning, and possibly bending of axonal bundles with the advancement of age.[1,50] As stated above, this could be explained as evidence of continuous remodelling of WM during the life span which is more evident after the sixth decade. Notably, the age-related changes in the hippocampal connections might explain the amnestic changes in the population.[16,50]

The sex-specific WM integrity association of DTI and NODDI is sparsely covered in the literature. A few DTI studies evaluated sex differences and reported inconsistent findings. While some studies showed higher FA in the corpus callosum in females,[69-71] others suggested greater FA in males.[72,73] Males also showed significantly higher FA in the temporal,[74] temporoparietal,[71] cingulum and deep WM regions[73,75] such as superior longitudinal fasciculus, a tract implicated in language processing.[69] The inconsistency across these findings may be due to the heterogeneity of populations and differences in the analysis methods. In this study, we observed small sex differences in eight tracts, with higher FA in males and greater ODI and ISOVF in females which is consistent with previous studies in healthy adults.[1,50]

Reserve and resilience factors are important modulating parameters between brain injury and cognitive outcomes. Past studies showed education/occupation as a “proxy” for cognitive

For Review Only

reserve and related differences in fiber tract integrity.[76,77] Similarly, a previous study in the MCSA population demonstrated a significant association between cognitive reserve and FA of the genu.[78] Although this association was modest, the present study showed a positive association of DTI and NODDI measures with corpus callosum, cingulum, parahippocampal cingulum, superior longitudinal fasciculus, inferior fronto-occipital fasciculus, and inferior temporal WM, which is consistent with prior DTI studies.[76,78] However, the voxel-wise associations were not significant after correcting for multiple comparisons. The anatomical localization provides an insight into the biological mechanism of reserve capacity with brain maturation and plasticity.

For Review Only

Diffusion measures as markers of CVD

Although WMH is the most commonly used biomarker for CVD,[2] it only represents extensive WM damage and fails to measure the disruption or subtle changes of the underlying WM tracts. There is growing evidence supporting the utility of DTI to characterize the WM changes in CVD[40,79,80] even before the appearance of WMH and cognitive decline. Notably, the observed decrease in diffusion directionality and an increase in the extent of water diffusion in conjunction with WMH are consistent with prior findings.[40,81,80] Consistent with this idea, a recent study using FW imaging demonstrated a greater contribution of CVD markers than AD biomarkers (CSF and PET) in memory clinic patients.[17]

Similar to the age effect, we found a strong association of WMH with corpus callosum and association fibers.[5] Although there is evidence for more vascular damage in the thinly myelinated anterior corpus callosum,[5,82,83] the present study showed slight variations across the measures. Importantly, conventional DTI performed as well as NDI in detecting CVD changes.[84] The decreased density and dispersion of the neurites and increased FW might contrast the lack of specificity in FA and MD to explain the underlying histological changes associated with WMH. The NDI finding in the genu of the corpus callosum was in accordance with a prior NODDI study that explored the diabetic encephalopathy in subjects with cognitive impairment.[79] The only measure that did not show consistent associations with WMH was ODI. We believe this is due to ODI nominally being a property of only healthy neurites, and thus being more orthogonal to neuronal decay than the other NODDI measures or DTI.

Though the correlations with vascular risk were not a focus of the manuscript, as previously reported[82] we found that WM measures from traditional DTI (FA and MD) and NODDI (NDI specifically) were significantly associated with worsening vascular risk (**Results not shown).** Recent researchers focused on using global diffusion MRI as a CVD marker.[84-86] However, this work sheds light on the variability in regional associations suggesting a greater sensitivity and specificity of regional markers. Future work should be undertaken to widely validate and compare diffusion outcomes as CVD measures.

Diffusion measures and neuroimaging AD measures

The association between amyloid deposition and WM microstructure is still a matter of debate. A non-monotonic behavior was found between both measures in GM[87] and WM[88,89] in human studies. Consistent with our region level findings, prior DTI studies reported reduced FA in corpus callosum and fornix[90,91] in cognitively unimpaired individuals and increased axial diffusivity[92] and accelerated FA decrease[93] in the parahippocampal cingulum of amyloid positive individuals. As expected, the current study identified a significant global association between Aβ deposition and increased MD and ISOVF along with decreased NDI in medial temporal lobe grey-white matter junctions, which are consistent with a more recent study that reported lower neurite density in limbic and association fibers and higher medial temporal FW.[94] The medial temporal lobe is an early region of neuronal changes in AD, so the parahippocampal cingulum findings were as expected. We also found associations between tau and non-specific MD and ISOVF association in the inferior temporal WM (**Fig. 4**). These results are supported by a study of tau and NODDI in a transgenic AD model.[95] Our findings in the temporal lobe (hippocampal and parahippocampal regions) and the temporo-occipital fusiform gyrus suggest that NODDI may be able to provide more detailed information about neurite health in the presence of AD pathology.

For Review Only

Diffusion measures with Cognition

Association between WM DTI alterations and cognitive decline in the cognitively unimpaired, MCI, and AD populations have been reported previously.[40,78,81, 83] Although there was decreased FA and increased MD in association with cognitive decline, the exact sources of DTI signal were not studied. The present study is one of the earliest studies to compare DTI and NODDI on the

basis of their association with cognitive performance after accounting for amyloid and tau, which allows us to evaluate its utility as a CVD marker. As expected, both DTI and NODDI were significantly associated with global cognition and cognitive subdomain scores after adjusting for age, sex, cycle visit, and AD biomarkers. The overall pattern suggests that higher coherence and density, and lower FW concentration and tract complexity, both correlate with better cognitive performance.[16,96] Across the tracts, the strongest association of reduced WM integrity and worse global cognitive performance was observed in the corpus callosum. This is consistent with a previous DTI study in CVD that reported highly significant correlations of genu and splenium with global cognitive performance.[40] Impaired interhemispheric connection pathways contribute to multiple impaired cognitive functions such as impaired memory, psychomotor speed, frontal lobe mediated attention and executive function.[97,98] Additionally, these observations replicated our recent study in MCI that showed greater predictability of high FA of genu on better cognitive performance,[83] even after controlling for amyloid and tau PET.

Although there are contributions from other domains, we found that the associations with WM integrity and cognitive performance were mainly driven by attention. Importantly, our detailed investigation indicated that FW fraction in the corpus callosum predicted cognitive decline. In general, reduced neurite density correlated with worse cognitive performance with most of the tracts in all domains. Among these, the stronger association of NDI than FA in superior longitudinal fasciculus may be due to its proximity to the crossing fibers in the centrum semiovale,[99] which corresponds to higher FA and lower tract complexity. Another speculation may be that the superior longitudinal fasciculus is connecting lateral prefrontal to parietal brain areas, which are responsible for the multifaceted processes as we studied here. Notably, cingulum performed uniformly well across all domains and diffusion metrics to predict cognition. This bundle is the prominent WM tract that interconnects frontal, parietal, and medial temporal lobe and the posterior cingulate cortex. Surprisingly, the parahippocampal cingulum bundle, which connects the hippocampus to the rest of the brain areas, emerged as an important tract in visuospatial function. In contrast, deteriorations in parahippocampal cingulum have previously been implicated in association with episodic memory in older subjects[100] and AD.[101]

Diffusion metrics are suggested to be most strongly associated with processing speed.[85, 102] Therefore we also tested these hypotheses in the supplemental material and found that both

For Review Only

DTI and NODDI strongly predicted processing speed (Trail B and combined). As expected, commissural fibers had the greatest effect size.

The present study has several strengths and limitations. The main strength was the extensive analyses of single and multi-shell diffusion data on WM health and cognition. Also, this is the first study to assess the relationship between NODDI metrics and CVD and AD biomarkers together along with associations with cognitive performance. The inclusion of a representative sample population strengthens the generalizability of the findings. Our voxel-wise and regional findings mostly corroborated each other, and the slight differences may be due to partial volume effects, smoothing and more stringent FWE corrections. The major limitations are the cross-sectional nature of the study and the lack of histological confirmation of the observed associations. Furthermore, the regularization scheme used by the AMICO implementation of NODDI acts like a prior that gives a mild preference to some values of NDI, ISOVF, and especially ODI, which could be obscuring some differences between subjects. Future longitudinal research with multiple biophysical models[11] may provide more sensitive and conclusive findings.

In summary, the present study provides evidence of microstructural brain alterations associated with brain aging, disease pathologies and cognition. Although NODDI-derived indices provide additional insights into the underlying synaptic density, organization and FW content that are mixed together when measured with DTI, traditional FA and MD measures are comparable in capturing disease-specific pathologies and cognition. Among the indices, MD was the most significant parameter, which correlated well with FW fraction provided by ISOVF. Together, these findings suggest that traditional FA and MD measures perform well in detecting disease-specific pathologies and cognitive effects but more specific models such as NODDI will be needed to distinguish or explain these effects. Furthermore, the observed spatial heterogeneity in tracts across the metrics highlights the importance of multiple diffusion metrics to investigate changes in each WM region of the brain as a function of disease progression.

For Review Only

Acknowledgments

We would like to thank Lorraine Vassallo for her help with editing the manuscript. We thank all the study participants and staff in the Mayo Clinic Study of Aging, Mayo Alzheimer's Disease Research Center, and Aging Dementia Imaging Research laboratory at the Mayo Clinic for making this study possible. We gratefully acknowledge the support of NVIDIA Corporation for the donation of the Quadro P5000 GPU used in this research.

Funding

This work was supported by NIH grants R01 NS097495 (PI: Vemuri), U01 AG06786 (PI: Petersen/Mielke/Jack), R01 AG56366 (PI: Vemuri), P50 AG16574 (PI: Petersen), R37 AG11378 (PI: Jack), R01 AG41851 (PIs: Jack and Knopman); the Gerald and Henrietta Rauenhorst Foundation grant, Alzheimer's Drug Discovery Foundation (ADDF), the Alexander Family Alzheimer's Disease Research Professorship of the Mayo Foundation, Liston Award, Elsie and Marvin Dekelboum Family Foundation, Schuler Foundation, Opus building NIH grant C06 RR018898, and was made possible by Rochester Epidemiology Project (R01 AG34676).

Competing Interests

The authors report no competing interests related to the submitted manuscript.

Supplementary material

Supplementary material is available at *Brain* online.

For Review Only

References

1. Cox SR, Ritchie SJ, Tucker-Drob EM, et al. Ageing and brain white matter structure in 3,513 UK Biobank participants. *Nature communications*. 2016;7:13629. doi:10.1038/ncomms13629

2. Wardlaw JM, Smith EE, Biessels GJ, et al. Neuroimaging standards for research into small vessel disease and its contribution to ageing and neurodegeneration. *The Lancet Neurology*. 2013;12(8):822-38. doi:10.1016/s1474-4422(13)70124-8

3. Nasrabady SE, Rizvi B, Goldman JE, Brickman AM. White matter changes in Alzheimer's disease: a focus on myelin and oligodendrocytes. *Acta neuropathologica communications*. 2018;6(1):22. doi:10.1186/s40478-018-0515-3

4. Caballero MÁ A, Song Z, Rubinski A, et al. Age-dependent amyloid deposition is associated with white matter alterations in cognitively normal adults during the adult life span. *Alzheimer's & dementia : the journal of the Alzheimer's Association*. 2020;16(4):651-661. doi:10.1002/alz.12062

5. Cox SR, Lyall DM, Ritchie SJ, et al. Associations between vascular risk factors and brain MRI indices in UK Biobank. *European heart journal*. 2019;40(28):2290-2300. doi:10.1093/eurheartj/ehz100

6. Pines AR, Cieslak M, Larsen B, et al. Leveraging multi-shell diffusion for studies of brain development in youth and young adulthood. *Developmental cognitive neuroscience*. 2020;43:100788. doi:10.1016/j.dcn.2020.100788

7. Schilling KG, Janve V, Gao Y, Stepniewska I, Landman BA, Anderson AW. Histological validation of diffusion MRI fiber orientation distributions and dispersion. *NeuroImage*. 2018;165:200-221. doi:10.1016/j.neuroimage.2017.10.046

8. Jensen JH, Helpern JA, Ramani A, Lu H, Kaczynski K. Diffusional kurtosis imaging: the quantification of non-gaussian water diffusion by means of magnetic resonance imaging. *Magnetic resonance in medicine*. 2005;53(6):1432-40. doi:10.1002/mrm.20508

9. Jensen JH, Helpern JA. MRI quantification of non-Gaussian water diffusion by kurtosis analysis. *NMR in biomedicine*. 2010;23(7):698-710. doi:10.1002/nbm.1518

10. Zhang H, Schneider T, Wheeler-Kingshott CA, Alexander DC. NODDI: practical in vivo neurite orientation dispersion and density imaging of the human brain. *NeuroImage*. 2012;61(4):1000-16. doi:10.1016/j.neuroimage.2012.03.072

11. Beck D, de Lange AG, Maximov, II, et al. White matter microstructure across the adult lifespan: A mixed longitudinal and cross-sectional study using advanced diffusion models and brain-age prediction. *NeuroImage*. 2020;224:117441. doi:10.1016/j.neuroimage.2020.117441

12. Toschi N, Gisbert RA, Passamonti L, Canals S, De Santis S. Multishell diffusion imaging reveals sex-specific trajectories of early white matter degeneration in normal aging. *Neurobiology of aging*. 2020;86:191-200. doi:10.1016/j.neurobiolaging.2019.11.014

For Review Only

13. Parker TD, Slattery CF, Zhang J, et al. Cortical microstructure in young onset Alzheimer's disease using neurite orientation dispersion and density imaging. *Human brain mapping*. 2018;39(7):3005-3017. doi:10.1002/hbm.24056

14. Slattery CF, Zhang J, Paterson RW, et al. ApoE influences regional white-matter axonal density loss in Alzheimer's disease. *Neurobiology of aging*. 2017;57:8-17. doi:10.1016/j.neurobiolaging.2017.04.021

15. Vogt NM, Hunt JF, Adluru N, et al. Cortical Microstructural Alterations in Mild Cognitive Impairment and Alzheimer's Disease Dementia. *Cerebral cortex (New York, NY : 1991)*. 2020;30(5):2948-2960. doi:10.1093/cercor/bhz286

16. Wen Q, Mustafi SM, Li J, et al. White matter alterations in early-stage Alzheimer's disease: A tract-specific study. *Alzheimer's & dementia (Amsterdam, Netherlands)*. 2019;11:576-587. doi:10.1016/j.dadm.2019.06.003

17. Finsterwalder S, Vlegels N, Gesierich B, et al. Small vessel disease more than Alzheimer's disease determines diffusion MRI alterations in memory clinic patients. *Alzheimer's & dementia : the journal of the Alzheimer's Association*. 2020;16(11):1504-1514. doi:10.1002/alz.12150

18. Bells S, Lefebvre J, Prescott SA, et al. Changes in White Matter Microstructure Impact Cognition by Disrupting the Ability of Neural Assemblies to Synchronize. *The Journal of neuroscience : the official journal of the Society for Neuroscience*. 2017;37(34):8227-8238. doi:10.1523/jneurosci.0560-17.2017

19. Filley CM, Fields RD. White matter and cognition: making the connection. *Journal of neurophysiology*. 2016;116(5):2093-2104. doi:10.1152/jn.00221.2016

20. Jelescu IO, Budde MD. Design and validation of diffusion MRI models of white matter. *Frontiers in physics*. 2017;28doi:10.3389/fphy.2017.00061

21. Novikov DS, Veraart J, Jelescu IO, Fieremans E. Rotationally-invariant mapping of scalar and orientational metrics of neuronal microstructure with diffusion MRI. *NeuroImage*. 2018;174:518-538. doi:10.1016/j.neuroimage.2018.03.006

22. Zhang YZ, Chang C, Wei XE, Fu JL, Li WB. Comparison of diffusion tensor image study in association fiber tracts among normal, amnestic mild cognitive impairment, and Alzheimer's patients. *Neurology India*. 2011;59(2):168-73. doi:10.4103/0028-3886.79129

23. Rocca WA, Yawn BP, St Sauver JL, Grossardt BR, Melton LJ, 3rd. History of the Rochester Epidemiology Project: half a century of medical records linkage in a US population. *Mayo Clinic proceedings*. 2012;87(12):1202-13. doi:10.1016/j.mayocp.2012.08.012

24. St Sauver JL, Grossardt BR, Yawn BP, et al. Data resource profile: the Rochester Epidemiology Project (REP) medical records-linkage system. *Int J Epidemiol*. 2012;41(6):1614-24. doi:10.1093/ije/dys195

For Review Only

25. Petersen RC, Roberts RO, Knopman DS, et al. Prevalence of mild cognitive impairment is higher in men. The Mayo Clinic Study of Aging. *Neurology*. 2010;75(10):889-97.

26. Caruyer E, Lenglet C, Sapiro G, Deriche R. Design of multishell sampling schemes with uniform coverage in diffusion MRI. *Magnetic resonance in medicine*. 2013;69(6):1534-40. doi:10.1002/mrm.24736

27. Reid RI, Nedelska Z, Schwarz CG, et al. *Diffusion specific segmentation: skull stripping with diffusion MRI data alone*. Computational diffusion MRI mathematics and visualization. Cham: Springer; 2018:67– 80.

28. Veraart J, Novikov DS, Christiaens D, Ades-Aron B, Sijbers J, Fieremans E. Denoising of diffusion MRI using random matrix theory. *NeuroImage*.2016;142:394-406. doi:10.1016/j.neuroimage.2016.08.016

29. Andersson JLR, Sotiropoulos SN. An integrated approach to correction for off-resonance effects and subject movement in diffusion MR imaging. *NeuroImage*. 2016;125:1063-1078. doi:10.1016/j.neuroimage.2015.10.019

30. Kellner E, Dhital B, Kiselev VG, Reisert M. Gibbs-ringing artifact removal based on local subvoxel-shifts. *Magnetic resonance in medicine*. 2016;76(5):1574-1581. doi:10.1002/mrm.26054

31. Koay CG, Ozarslan E, Basser PJ. A signal transformational framework for breaking the noise floor and its applications in MRI. *Journal of magnetic resonance (San Diego, Calif : 1997)*. 2009;197(2):108-19. doi:10.1016/j.jmr.2008.11.015

32. Garyfallidis E, Brett M, Amirbekian B, et al. Dipy, a library for the analysis of diffusion MRI data. *Frontiers in neuroinformatics*. 2014;8:8. doi:10.3389/fninf.2014.00008

33. Daducci A, Canales-Rodríguez EJ, Zhang H, Dyrby TB, Alexander DC, Thiran JP. Accelerated Microstructure Imaging via Convex Optimization (AMICO) from diffusion MRI data. *NeuroImage*. 2015;105:32-44. doi:10.1016/j.neuroimage.2014.10.026

34. Jack CR, Jr., Wiste HJ, Weigand SD, et al. Defining imaging biomarker cut points for brain aging and Alzheimer's disease. *Alzheimer's & dementia : the journal of the Alzheimer's Association*. 2017;13(3):205-216. doi:10.1016/j.jalz.2016.08.005

35. Graff-Radford J, Arenaza-Urquijo EM, Knopman DS, et al. White matter hyperintensities: relationship to amyloid and tau burden. *Brain : a journal of neurology*. 2019;142(8):2483-2491. doi:10.1093/brain/awz162

36. Zhang Y, Brady M, Smith S. Segmentation of brain MR images through a hidden Markov random field model and the expectation-maximization algorithm. *IEEE transactions on medical imaging*. 2001;20(1):45-57. doi:10.1109/42.906424

For Review Only

37. Manjón JV, Coupé P, Martí-Bonmatí L, Collins DL, Robles M. Adaptive non-local means denoising of MR images with spatially varying noise levels. *Journal of magnetic resonance imaging : JMRI*. 2010;31(1):192-203. doi:10.1002/jmri.22003

38. Roberts RO, Geda YE, Knopman DS, et al. The Mayo Clinic Study of Aging: design and sampling, participation, baseline measures and sample characteristics. *Neuroepidemiology*. 2008;30(1):58-69.

39. Vemuri P, Lesnick TG, Przybelski SA, et al. Association of lifetime intellectual enrichment with cognitive decline in the older population. *JAMA neurology*. 2014;71(8):1017-24. doi:10.1001/jamaneurol.2014.963

40. Tuladhar AM, van Norden AG, de Laat KF, et al. White matter integrity in small vessel disease is related to cognition. *NeuroImage Clinical*. 2015;7:518-24. doi:10.1016/j.nicl.2015.02.003

41. Merluzzi AP, Dean DC, Adluru N, et al. Age-dependent differences in brain tissue microstructure assessed with neurite orientation dispersion and density imaging. *Neurobiology of aging*. 2016;43:79-88. doi:10.1016/j.neurobiolaging.2016.03.026

42. Bendlin BB, Fitzgerald ME, Ries ML, et al. White matter in aging and cognition: a cross-sectional study of microstructure in adults aged eighteen to eighty-three. *Developmental neuropsychology*. 2010;35(3):257-77. doi:10.1080/87565641003696775

43. Oishi K, Faria A, Jiang H, et al. Atlas-based whole brain white matter analysis using large deformation diffeomorphic metric mapping: application to normal elderly and Alzheimer's disease participants. *Neuroimage*. 2009;46(2):486-99. doi:10.1016/j.neuroimage.2009.01.002

44. Avants BB, Tustison NJ, Song G, Cook PA, Klein A, Gee JC. A reproducible evaluation of ANTs similarity metric performance in brain image registration. *NeuroImage*. 2011;54(3):2033-44. doi:10.1016/j.neuroimage.2010.09.025

45. Ashburner J, Friston KJ. Voxel-based morphometry--the methods. *NeuroImage*. Jun 2000;11(6):805-21. doi:10.1006/nimg.2000.0582

46. Alexander AL, Lee JE, Lazar M, Field AS. Diffusion tensor imaging of the brain. *Neurotherapeutics : the journal of the American Society for Experimental NeuroTherapeutics*. 2007;4(3):316-29. doi:10.1016/j.nurt.2007.05.011

47. Jeurissen B, Leemans A, Tournier JD, Jones DK, Sijbers J. Investigating the prevalence of complex fiber configurations in white matter tissue with diffusion magnetic resonance imaging. *Human brain mapping*. 2013;34(11):2747-66. doi:10.1002/hbm.22099

48. Chang YS, Owen JP, Pojman NJ, et al. White Matter Changes of Neurite Density and Fiber Orientation Dispersion during Human Brain Maturation. *PloS one*. 2015;10(6):e0123656. doi:10.1371/journal.pone.0123656

For Review Only

49. Eaton-Rosen Z, Melbourne A, Orasanu E, et al. Longitudinal measurement of the developing grey matter in preterm subjects using multi-modal MRI. *NeuroImage*. 2015;111:580-9. doi:10.1016/j.neuroimage.2015.02.010

50. Kodiweera C, Alexander AL, Harezlak J, McAllister TW, Wu YC. Age effects and sex differences in human brain white matter of young to middle-aged adults: A DTI, NODDI, and q-space study. *NeuroImage*. 2016;128:180-192. doi:10.1016/j.neuroimage.2015.12.033

51. Mah A, Geeraert B, Lebel C. Detailing neuroanatomical development in late childhood and early adolescence using NODDI. *PloS one*. 2017;12(8):e0182340. doi:10.1371/journal.pone.0182340

52. Timmers I, Zhang H, Bastiani M, Jansma BM, Roebroeck A, Rubio-Gozalbo ME. White matter microstructure pathology in classic galactosemia revealed by neurite orientation dispersion and density imaging. *Journal of inherited metabolic disease*. 2015;38(2):295-304. doi:10.1007/s10545-014-9780-x

53. Sato K, Kerever A, Kamagata K, et al. Understanding microstructure of the brain by comparison of neurite orientation dispersion and density imaging (NODDI) with transparent mouse brain. *Acta radiologica open*. 2017;6(4):2058460117703816. doi:10.1177/2058460117703816

54. Sepehrband F, Clark KA, Ullmann JF, et al. Brain tissue compartment density estimated using diffusion-weighted MRI yields tissue parameters consistent with histology. *Human brain mapping*. 2015;36(9):3687-702. doi:10.1002/hbm.22872

55. Fukutomi H, Glasser MF, Zhang H, et al. Neurite imaging reveals microstructural variations in human cerebral cortical gray matter. *NeuroImage*. 2018;182:488-499. doi:10.1016/j.neuroimage.2018.02.017

56. Genc S, Malpas CB, Holland SK, Beare R, Silk TJ. Neurite density index is sensitive to age related differences in the developing brain. *NeuroImage*. 2017;148:373-380. doi:10.1016/j.neuroimage.2017.01.023

57. Lebel C, Gee M, Camicioli R, Wieler M, Martin W, Beaulieu C. Diffusion tensor imaging of white matter tract evolution over the lifespan. *NeuroImage*. 2012;60(1):340-52. doi:10.1016/j.neuroimage.2011.11.094

58. Huang H, Zhang J, Wakana S, et al. White and gray matter development in human fetal, newborn and pediatric brains. *NeuroImage*. 2006;33(1):27-38. doi:10.1016/j.neuroimage.2006.06.009

59. Billiet T, Vandenbulcke M, Mädler B, et al. Age-related microstructural differences quantified using myelin water imaging and advanced diffusion MRI. *Neurobiology of aging*. 2015;36(6):2107-21. doi:10.1016/j.neurobiolaging.2015.02.029

60. Kunz N, Zhang H, Vasung L, et al. Assessing white matter microstructure of the newborn with multi-shell diffusion MRI and biophysical compartment models. *NeuroImage*. 2014;96:288-99. doi:10.1016/j.neuroimage.2014.03.057

For Review Only

61. Nazeri A, Chakravarty MM, Rotenberg DJ, et al. Functional consequences of neurite orientation dispersion and density in humans across the adult lifespan. *The Journal of neuroscience : the official journal of the Society for Neuroscience*. 2015;35(4):1753-62. doi:10.1523/jneurosci.3979-14.2015

62. Slater DA, Melie-Garcia L, Preisig M, Kherif F, Lutti A, Draganski B. Evolution of white matter tract microstructure across the life span. *Human brain mapping*. 2019;40(7):2252-2268. doi:10.1002/hbm.24522

63. Pasternak O, Sochen N, Gur Y, Intrator N, Assaf Y. Free water elimination and mapping from diffusion MRI. *Magnetic resonance in medicine*. 2009;62(3):717-30. doi:10.1002/mrm.22055

64. Wang Y, Wang Q, Haldar JP, et al. Quantification of increased cellularity during inflammatory demyelination. *Brain : a journal of neurology*. 2011;134(12):3590-601. doi:10.1093/brain/awr307

65. Chad JA, Pasternak O, Salat DH, Chen JJ. Re-examining age-related differences in white matter microstructure with free-water corrected diffusion tensor imaging. *Neurobiology of aging*. 2018;71:161-170. doi:10.1016/j.neurobiolaging.2018.07.018

66. Meier-Ruge W, Ulrich J, Brühlmann M, Meier E. Age-related white matter atrophy in the human brain. *Annals of the New York Academy of Sciences*. 1992;673:260-9. doi:10.1111/j.1749-6632.1992.tb27462.x

67. Bartzokis G, Sultzer D, Lu PH, Nuechterlein KH, Mintz J, Cummings JL. Heterogeneous age-related breakdown of white matter structural integrity: implications for cortical "disconnection" in aging and Alzheimer's disease. *Neurobiology of aging*. 2004;25(7):843-51. doi:10.1016/j.neurobiolaging.2003.09.005

68. Kochunov P, Thompson PM, Lancaster JL, et al. Relationship between white matter fractional anisotropy and other indices of cerebral health in normal aging: tract-based spatial statistics study of aging. *NeuroImage*. 2007;35(2):478-87. doi:10.1016/j.neuroimage.2006.12.021

69. Kanaan RA, Allin M, Picchioni M, et al. Gender differences in white matter microstructure. *PloS one*. 2012;7(6):e38272. doi:10.1371/journal.pone.0038272

70. Oh JS, Song IC, Lee JS, et al. Tractography-guided statistics (TGIS) in diffusion tensor imaging for the detection of gender difference of fiber integrity in the midsagittal and parasagittal corpora callosa. *NeuroImage*. 2007;36(3):606-16. doi:10.1016/j.neuroimage.2007.03.020

71. Phillips OR, Clark KA, Luders E, et al. Superficial white matter: effects of age, sex, and hemisphere. *Brain connectivity*. 2013;3(2):146-59. doi:10.1089/brain.2012.0111

72. Huster RJ, Westerhausen R, Herrmann CS. Sex differences in cognitive control are associated with midcingulate and callosal morphology. *Brain structure & function*. 2011;215(3-4):225-35. doi:10.1007/s00429-010-0289-2

For Review Only

73. Inano S, Takao H, Hayashi N, Abe O, Ohtomo K. Effects of age and gender on white matter integrity. *AJNR American journal of neuroradiology*. 2011;32(11):2103-9. doi:10.3174/ajnr.A2785

74. Hsu JC, Tanel RE, Lee BK, et al. Differences in accessory pathway location by sex and race. *Heart rhythm*. Jan 2010;7(1):52-6. doi:10.1016/j.hrthm.2009.09.023

75. Menzler K, Belke M, Wehrmann E, et al. Men and women are different: diffusion tensor imaging reveals sexual dimorphism in the microstructure of the thalamus, corpus callosum and cingulum. *NeuroImage*. 2011;54(4):2557-62. doi:10.1016/j.neuroimage.2010.11.029

76. Arenaza-Urquijo EM, Bosch B, Sala-Llonch R, et al. Specific anatomic associations between white matter integrity and cognitive reserve in normal and cognitively impaired elders. *The American journal of geriatric psychiatry : official journal of the American Association for Geriatric Psychiatry*. 2011;19(1):33-42. doi:10.1097/JGP.0b013e3181e448e1

77. Teipel SJ, Meindl T, Wagner M, et al. Longitudinal changes in fiber tract integrity in healthy aging and mild cognitive impairment: a DTI follow-up study. *Journal of Alzheimer's disease : JAD*. 2010;22(2):507-22. doi:10.3233/jad-2010-100234

78. Vemuri P, Lesnick TG, Knopman DS, et al. Amyloid, Vascular, and Resilience Pathways Associated with Cognitive Aging. *Annals of neurology*. 2019;86(6):866-877. doi:10.1002/ana.25600

79. Xiong Y, Zhang S, Shi J, Fan Y, Zhang Q, Zhu W. Application of neurite orientation dispersion and density imaging to characterize brain microstructural abnormalities in type-2 diabetics with mild cognitive impairment. *Journal of magnetic resonance imaging : JMRI*. 2019;50(3):889-898. doi:10.1002/jmri.26687

80. Croall ID, Lohner V, Moynihan B, et al. Using DTI to assess white matter microstructure in cerebral small vessel disease (SVD) in multicentre studies. *Clinical science (London, England : 1979)*. Jun 1 2017;131(12):1361-1373. doi:10.1042/cs20170146

81. Tu MC, Lo CP, Huang CF, et al. Effectiveness of diffusion tensor imaging in differentiating early-stage subcortical ischemic vascular disease, Alzheimer's disease and normal ageing. *PloS one*. 2017;12(4):e0175143. doi:10.1371/journal.pone.0175143

82. Vemuri P, Lesnick TG, Przybelski SA, et al. Development of a cerebrovascular magnetic resonance imaging biomarker for cognitive aging. *Annals of neurology*. 2018;84(5):705-716. doi:10.1002/ana.25346

83. Raghavan S, Przybelski SA, Reid RI, et al. Reduced fractional anisotropy of the genu of the corpus callosum as a cerebrovascular disease marker and predictor of longitudinal cognition in MCI. *Neurobiology of aging*. 2020;96:176-183. doi:10.1016/j.neurobiolaging.2020.09.005

For Review Only

84. Duering M, Finsterwalder S, Baykara E, et al. Free water determines diffusion alterations and clinical status in cerebral small vessel disease. *Alzheimer's & dementia : the journal of the Alzheimer's Association*. 2018;14(6):764-774. doi:10.1016/j.jalz.2017.12.007

85. Baykara E, Gesierich B, Adam R, et al. A Novel Imaging Marker for Small Vessel Disease Based on Skeletonization of White Matter Tracts and Diffusion Histograms. *Annals of neurology*. 2016;80(4):581-92. doi:10.1002/ana.24758

86. Maillard P, Fletcher E, Singh B, et al. Cerebral white matter free water: A sensitive biomarker of cognition and function. *Neurology*. 2019;92(19):e2221-e2231. doi:10.1212/wnl.0000000000007449

87. Montal V, Vilaplana E, Alcolea D, et al. Cortical microstructural changes along the Alzheimer's disease continuum. *Alzheimer's & dementia : the journal of the Alzheimer's Association*. 2018;14(3):340-351. doi:10.1016/j.jalz.2017.09.013

88. Dong JW, Jelescu IO, Ades-Aron B, et al. Diffusion MRI biomarkers of white matter microstructure vary nonmonotonically with increasing cerebral amyloid deposition. *Neurobiology of aging*. 2020;89:118-128. doi:10.1016/j.neurobiolaging.2020.01.009

89. Wolf D, Fischer FU, Scheurich A, Fellgiebel A. Non-Linear Association between Cerebral Amyloid Deposition and White Matter Microstructure in Cognitively Healthy Older Adults. *Journal of Alzheimer's disease : JAD*. 2015;47(1):117-27. doi:10.3233/jad-150049

90. Gold BT, Zhu Z, Brown CA, et al. White matter integrity is associated with cerebrospinal fluid markers of Alzheimer's disease in normal adults. *Neurobiology of aging*. 2014;35(10):2263-71. doi:10.1016/j.neurobiolaging.2014.04.030

91. Chao LL, Decarli C, Kriger S, et al. Associations between white matter hyperintensities and β amyloid on integrity of projection, association, and limbic fiber tracts measured with diffusion tensor MRI. *PloS one*. 2013;8(6):e65175. doi:10.1371/journal.pone.0065175

92. Molinuevo JL, Ripolles P, Simó M, et al. White matter changes in preclinical Alzheimer's disease: a magnetic resonance imaging-diffusion tensor imaging study on cognitively normal older people with positive amyloid β protein 42 levels. *Neurobiology of aging*. 2014;35(12):2671-2680. doi:10.1016/j.neurobiolaging.2014.05.027

93. Rieckmann A, Van Dijk KR, Sperling RA, Johnson KA, Buckner RL, Hedden T. Accelerated decline in white matter integrity in clinically normal individuals at risk for Alzheimer's disease. *Neurobiology of aging*. 2016;42:177-88. doi:10.1016/j.neurobiolaging.2016.03.016

94. Reas ET, Hagler DJ, Kuperman JM, et al. Associations Between Microstructure, Amyloid, and Cognition in Amnestic Mild Cognitive Impairment and Dementia. *Journal of Alzheimer's disease : JAD*. 2020;73(1):347-357. doi:10.3233/jad-190871

For Review Only

95. Colgan N, Siow B, O'Callaghan JM, et al. Application of neurite orientation dispersion and density imaging (NODDI) to a tau pathology model of Alzheimer's disease. *NeuroImage*. 2016;125:739-744. doi:10.1016/j.neuroimage.2015.10.043

96. Fu X, Shrestha S, Sun M, et al. Microstructural White Matter Alterations in Mild Cognitive Impairment and Alzheimer's Disease : Study Based on Neurite Orientation Dispersion and Density Imaging (NODDI). *Clinical neuroradiology*. 2020;30(3):569-579. doi:10.1007/s00062-019-00805-0

97. Jokinen H, Ryberg C, Kalska H, et al. Corpus callosum atrophy is associated with mental slowing and executive deficits in subjects with age-related white matter hyperintensities: the LADIS Study. *Journal of neurology, neurosurgery, and psychiatry*. 2007;78(5):491-6. doi:10.1136/jnnp.2006.096792

98. Román GC, Erkinjuntti T, Wallin A, Pantoni L, Chui HC. Subcortical ischaemic vascular dementia. *The Lancet Neurology*. 2002;1(7):426-36. doi:10.1016/s1474-4422(02)00190-4

99. Douaud G, Jbabdi S, Behrens TE, et al. DTI measures in crossing-fibre areas: increased diffusion anisotropy reveals early white matter alteration in MCI and mild Alzheimer's disease. *NeuroImage*. 2011;55(3):880-90. doi:10.1016/j.neuroimage.2010.12.008

100. Ezzati A, Katz MJ, Lipton ML, Zimmerman ME, Lipton RB. Hippocampal volume and cingulum bundle fractional anisotropy are independently associated with verbal memory in older adults. *Brain imaging and behavior*. 2016;10(3):652-9. doi:10.1007/s11682-015-9452-y

101. Kantarci K, Murray ME, Schwarz CG, et al. White-matter integrity on DTI and the pathologic staging of Alzheimer's disease. *Neurobiology of aging*. 2017;56:172-179. doi:10.1016/j.neurobiolaging.2017.04.024

102. Konieczny MJ, Dewenter A, Telgte AT, et al. Multi-shell diffusion MRI models for white matter characterization in cerebral small vessel disease. *Neurology*. 2020;doi:10.1212/wnl.0000000000011213

For Review Only

Table 1. Characteristics table with the mean (SD) listed for the continuous variables and count (%) for the categorical variables.

Characteristic	All Participants n = 328
Male, no. (%)	169 (52%)
Age, yrs	68.3 (13.1)
E4 Carrier, no. (%)	89 (30%)
Education/Occupation	12.7 (2.4)
CMC	1.7 (1.5)
MMSE	28.3 (2.1)
zGlobal	0.19 (1.40)
zMemory	0.14 (1.41)
zAttention	0.06 (1.37)
zLanguage	-0.03 (1.34)
zVisual-spatial	0.23 (1.24)
Trails A	36.5 (19.2)
Trails B	92.2 (65.3)
Amyloid, SUVr	1.60 (0.49)
Amyloid Positive, no. (%)	115 (35%)
Tau, SUVr	1.21 (0.15)
Tau Positive, no. (%)	87 (27%)
WMH percentage	0.79 (0.94)
Diagnosis	
Cognitively Unimpaired, no. (%)	264 (80%)
MCI, no. (%)	57 (17%)
Dementia, no. (%)	7 (2%)

For Review Only

Figure 1A. FA, MD, NDI, ODI and ISOVF maps generated from a representative subject. **1B.** White matter tracts of interest from JHU atlas. FA- fractional anisotropy, MD- mean diffusivity, NDI- neurite density, ODI- orientation dispersion index, ISOVF – isotropic volume fraction

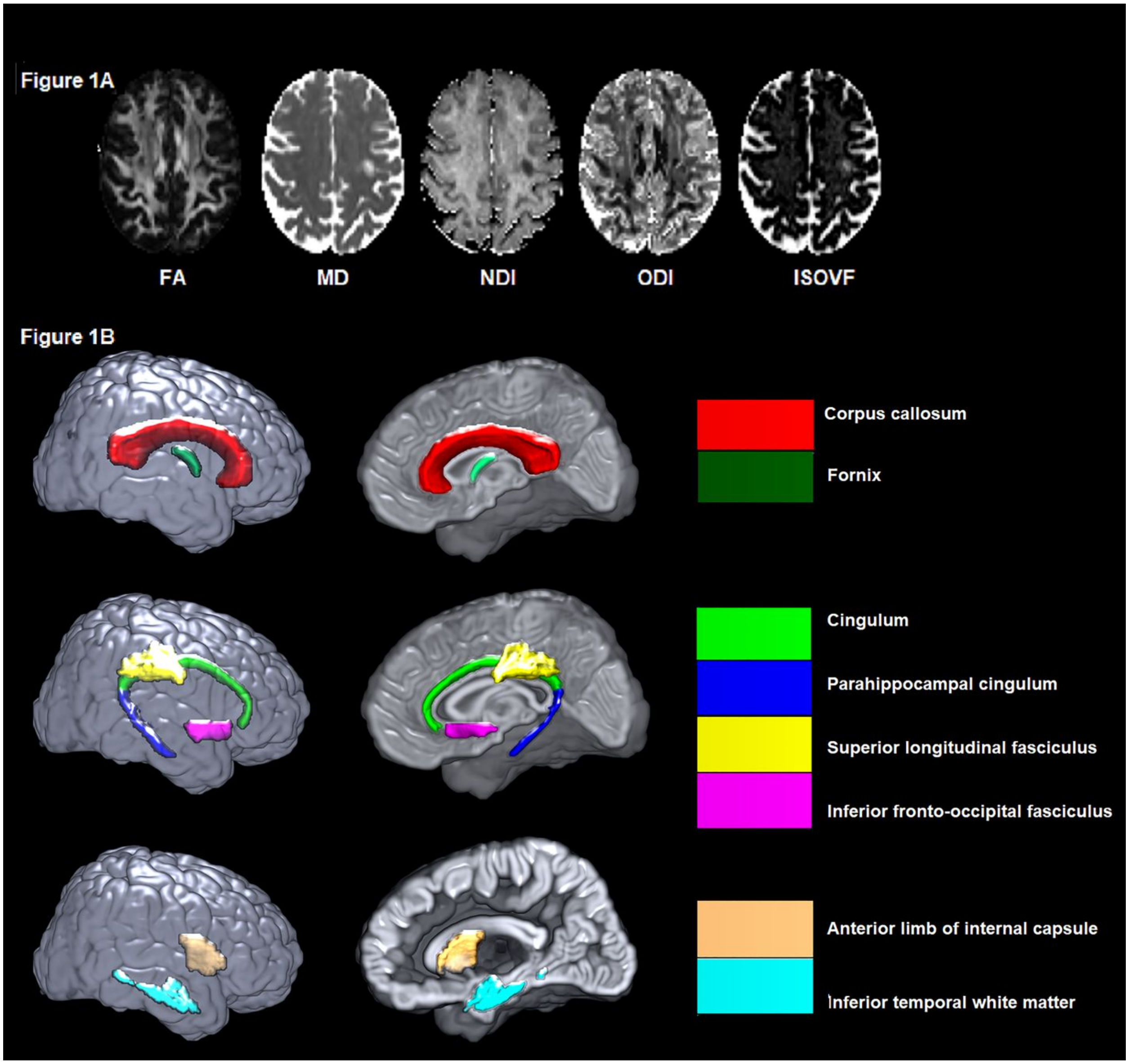

Figure 2: Correlation Matrix. Association between diffusion measures (FA and MD, FA and NDI, FA and ODI, MD and NDI, MD and ODI, MD and ISOVF). Color legend indicates the range of correlations, the size of the circle indicates the strength of the correlation, and the symbol "X" indicates the non-significant p value. FA- fractional anisotropy, MD- mean diffusivity, NDI- neurite density, ODI- orientation dispersion index, ISOVF – isotropic volume fraction, GCC- genu of corpus callosum, BCC- body of corpus callosum, and SCC- splenium of corpus callosum, FX- fornix, CGC- cingulum, CGH- parahippocampal cingulum, SLF- superior longitudinal fasciculus, IFOF- inferior fronto-occipital fasciculus, ITWM- inferior temporal WM, and ALIC- anterior limb of internal capsule.

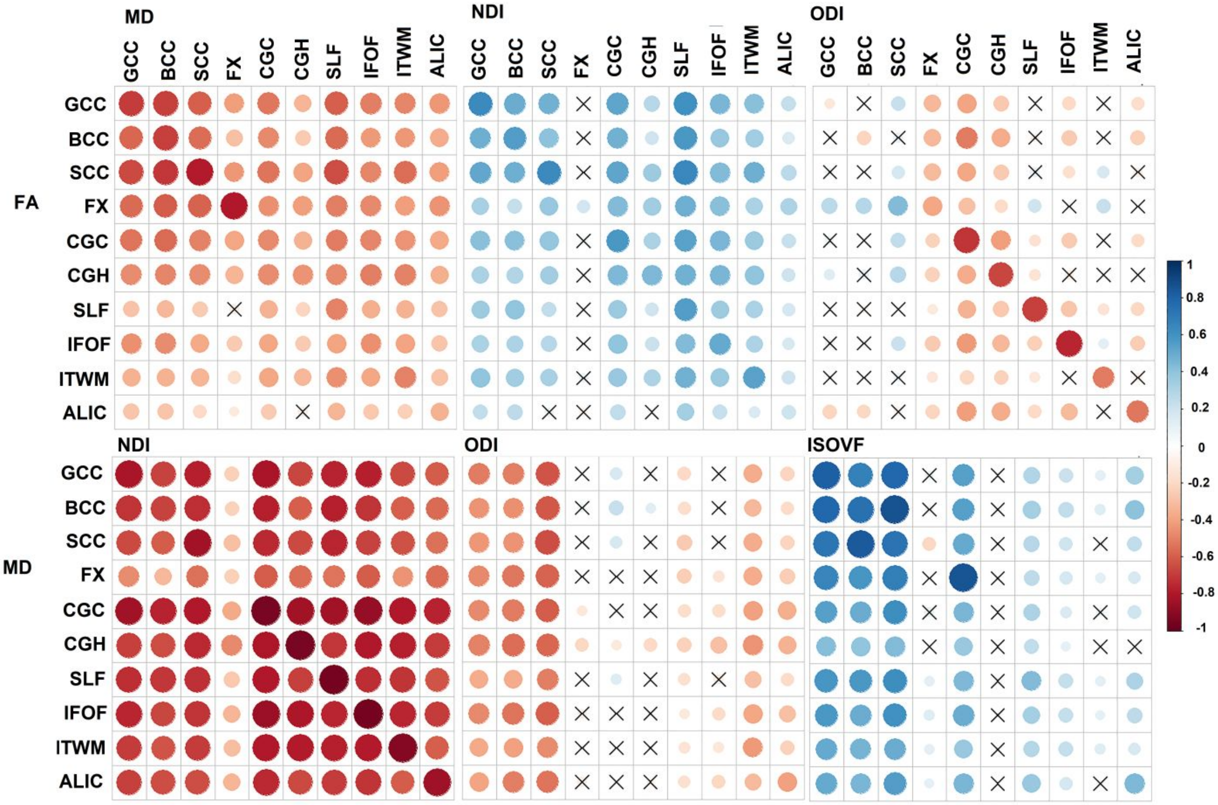

Figure 3: **Correlation Matrix**. **Correlation Matrix**. Association between demographics (age, sex, education/occupation) or white matter hyperintensity (WMH) or amyloid or tau and diffusion measures. Edu.occ represents education/occupation. Color legend indicates the range of correlations, the size of the circle indicates the strength of the correlation, and the symbol "X" indicates the non-significant p value. FA- fractional anisotropy, MD- mean diffusivity, NDI- neurite density, ODI- orientation dispersion index, ISOVF – isotropic volume fraction.

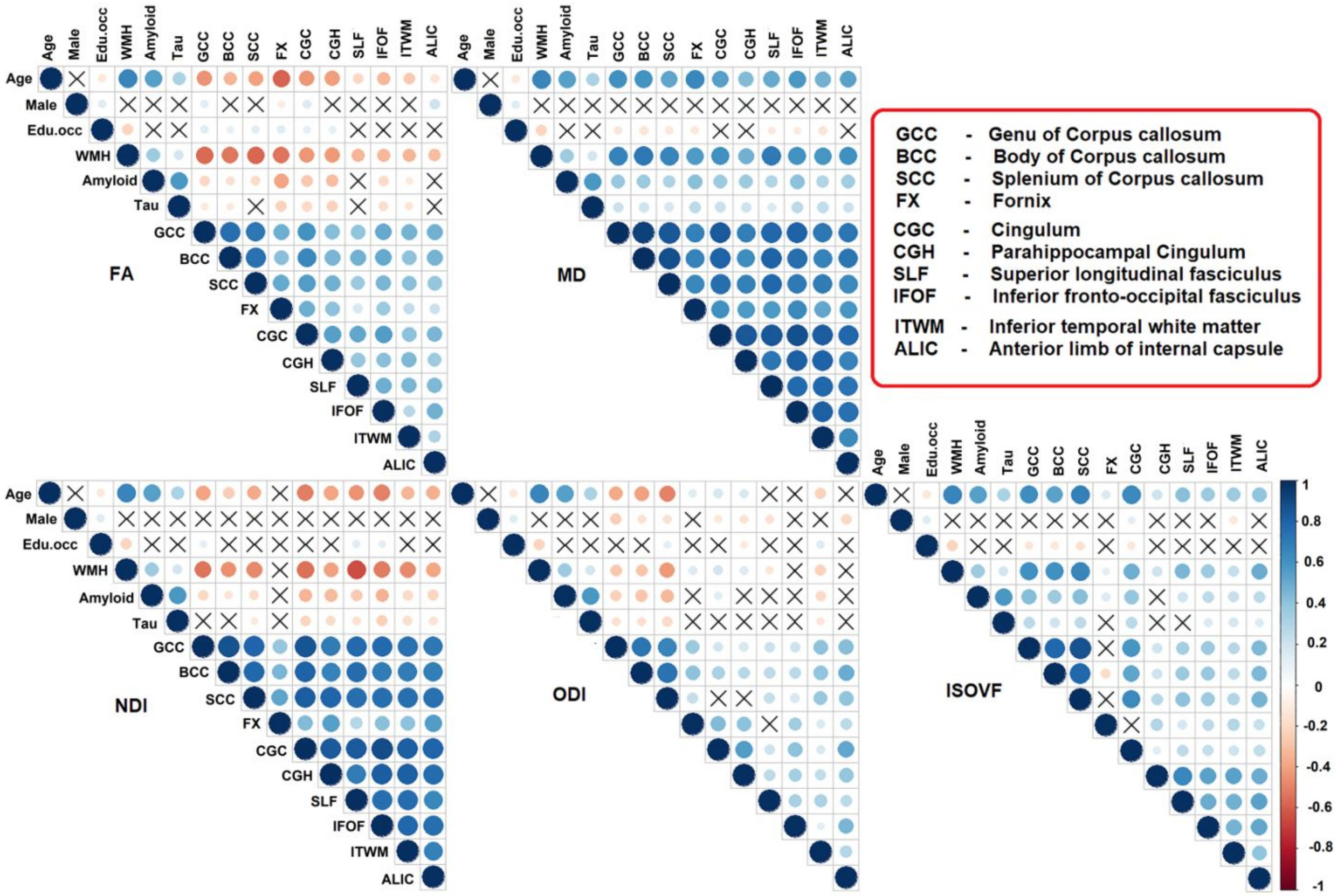

Figure 4: Association of diffusion metrics with white matter hyperintensity (WMH), amyloid, and tau after controlling for age, sex, and education/occupation. Different symbols below are used for each of the primary predictors. FA- fractional anisotropy, MD- mean diffusivity, NDI- neurite density, ODI- orientation dispersion index, ISOVF – isotropic volume fraction.

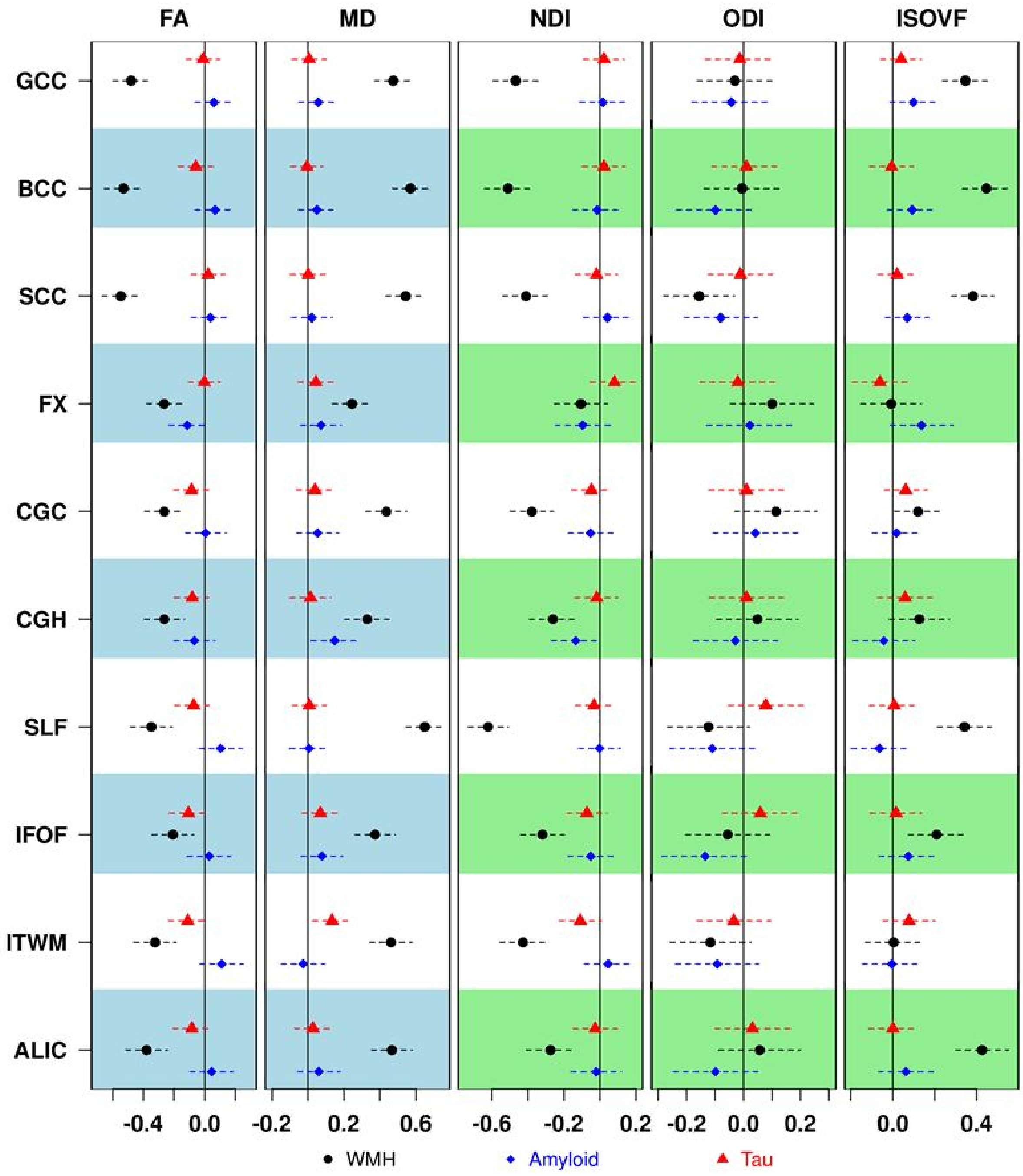

Figure 5: Association between age or white matter hyper intensity (WMH) and diffusion measures, significance level set at p<0.05, FWE corrected with an extend threshold K=100. (+ve and −ve represents the kind of association between variables). FA- fractional anisotropy, MD- mean diffusivity, NDI- neurite density, ODI- orientation dispersion index, ISOVF – isotropic volume fraction.

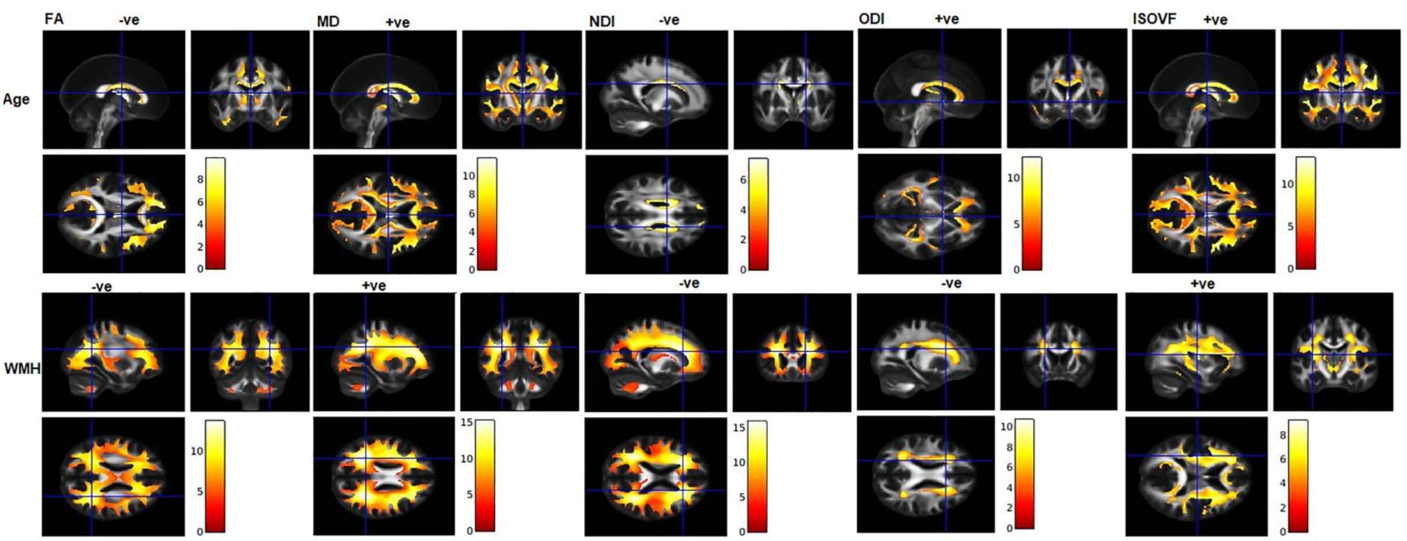

Figure 6: Association of diffusion metrics with cognition after controlling for age, sex and education/occupation, cycle visit, amyloid, and tau. Different symbols below are used for each of the diffusion measures. FA- fractional anisotropy, MD- mean diffusivity, NDI- neurite density, ODI- orientation dispersion index, ISOVF – isotropic volume fraction, GCC- genu of corpus callosum, BCC- body of corpus callosum, and SCC- splenium of corpus callosum

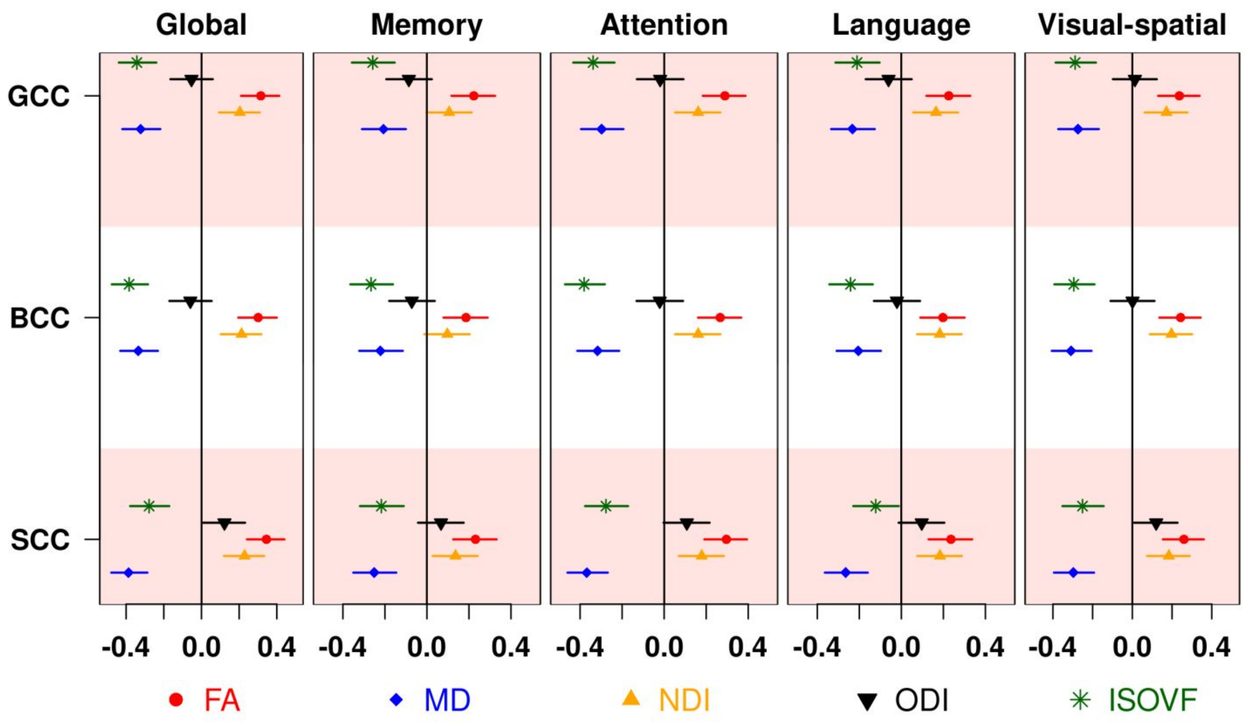

ONLINE SUPPLEMENTARY MATERIALS

Diffusion models reveal white matter microstructural changes with aging, pathology, and cognition

Sheelakumari Raghavan[1], Robert I. Reid[2], Scott A. Przybelski[3], Timothy G. Lesnick[3], Jonathan Graff-Radford[4], Christopher G. Schwarz[1], David S. Knopman[4], Michelle M. Mielke[3,4], Mary M. Machulda[5], Ronald C. Petersen[4], Clifford R. Jack Jr[1], Prashanthi Vemuri[1]

[1]Departments of Radiology, Mayo Clinic, Rochester, MN

[2]Information Technology, Mayo Clinic, Rochester, MN

[3]Health Sciences Research, Mayo Clinic, Rochester, MN

[4]Neurology, [5]Psychology, Mayo Clinic Rochester, MN

Corresponding Author: Prashanthi Vemuri, Ph.D.
Mayo Clinic and Foundation
200 First Street SW, Rochester, MN 55905

Phone: +1 507 538 0761, Fax:+1 507 284 9778, e-mail: **vemuri.prashanthi@mayo.edu**

For Review Only

Supplementary Table 1: Association between white matter hyper intensity (WMH) or amyloid or, tau and diffusion metrics after controlling for age, sex and education/occupation. FA- fractional anisotropy, MD- mean diffusivity, NDI- neurite density, ODI- orientation dispersion index, ISOVF – isotropic volume fraction, GCC- genu of corpus callosum, BCC- body of corpus callosum, and SCC- splenium of corpus callosum, FX- fornix, CGC- cingulum, CGH- parahippocampal cingulum, SLF- superior longitudinal fasciculus, IFOF- inferior fronto-occipital fasciculus, ITWM- inferior temporal WM, and ALIC- anterior limb of internal capsule.

Variable	Regression Coefficient (SE)	p	Regression Coefficient (SE)	p	Regression Coefficient (SE)	p	Regression Coefficient (SE)	p	Regression Coefficient (SE)	p
	FA		MD		NDI		ODI		ISOVF	
**WMH**										
**GCC**	**-0.479 (0.06)**	**<0.001**	**0.474 (0.05)**	**<0.001**	**-0.469 (0.06)**	**<0.001**	-0.03 (0.07)	0.65	**0.346 (0.06)**	**<0.001**
**BCC**	**-0.529 (0.06)**	**<0.001**	**0.569 (0.05)**	**<0.001**	**-0.511 (0.07)**	**<0.001**	-0.005 (0.07)	0.95	**0.447 (0.06)**	**<0.001**
**SCC**	**-0.547 (0.06)**	**<0.001**	**0.543 (0.06)**	**<0.001**	**-0.412 (0.07)**	**<0.001**	**-0.156 (0.06)**	**0.014**	**0.382 (0.05)**	**<0.001**
**FX**	**-0.264 (0.06)**	**<0.001**	**0.245 (0.06)**	**<0.001**	-0.106 (0.08)	0.16	0.1 (0.07)	0.18	-0.007 (0.07)	0.92
**CGC**	**-0.263 (0.07)**	**<0.001**	**0.435 (0.06)**	**<0.001**	**-0.378 (0.06)**	**<0.001**	0.114 (0.07)	0.12	**0.121 (0.06)**	**0.035**
**CGH**	**-0.263 (0.07)**	**<0.001**	**0.329 (0.06)**	**<0.001**	**-0.261 (0.08)**	**<0.001**	0.049 (0.07)	0.5	0.127 (0.07)	0.082
**SLF**	**-0.347 (0.07)**	**<0.001**	**0.649 (0.05)**	**<0.001**	**-0.621 (0.06)**	**<0.001**	-0.123 (0.07)	0.1	**0.342 (0.07)**	**<0.001**
**IFOF**	**-0.206 (0.07)**	**0.003**	**0.374 (0.06)**	**<0.001**	**-0.32 (0.06)**	**<0.001**	-0.056 (0.08)	0.45	**0.21 (0.07)**	**0.002**
**ITWM**	**-0.323 (0.07)**	**<0.001**	**0.461 (0.06)**	**<0.001**	**-0.428 (0.07)**	**<0.001**	-0.116 (0.07)	0.11	0.005 (0.07)	0.94
**ALIC**	**-0.378 (0.07)**	**<0.001**	**0.466 (0.06)**	**<0.001**	**-0.275 (0.07)**	**<0.001**	0.057 (0.07)	0.44	**0.426 (0.06)**	**<0.001**
**Amyloid**										
**GCC**	0.061 (0.06)	0.33	0.057 (0.06)	0.3	0.016 (0.07)	0.8	-0.043 (0.07)	0.54	0.099 (0.06)	0.083
**BCC**	0.068 (0.07)	0.31	0.05 (0.05)	0.34	-0.015 (0.07)	0.82	-0.098 (0.07)	0.16	0.093 (0.06)	0.12
**SCC**	0.038 (0.06)	0.54	0.022 (0.06)	0.7	0.041 (0.07)	0.54	-0.08 (0.07)	0.22	0.069 (0.05)	0.19
**FX**	-0.114 (0.06)	0.058	0.072 (0.06)	0.21	-0.096 (0.08)	0.22	0.022 (0.08)	0.77	0.138 (0.08)	0.071
**CGC**	0.007 (0.07)	0.91	0.054 (0.06)	0.37	-0.053 (0.06)	0.4	0.042 (0.08)	0.58	0.018 (0.06)	0.76
**CGH**	-0.066 (0.07)	0.34	**0.148 (0.07)**	**0.026**	-0.134 (0.07)	0.053	-0.028 (0.08)	0.71	-0.042 (0.08)	0.58
**SLF**	0.105 (0.07)	0.15	0.007 (0.06)	0.9	-0.002 (0.06)	0.97	-0.11 (0.08)	0.15	-0.064 (0.07)	0.35
**IFOF**	0.029 (0.07)	0.69	0.077 (0.06)	0.19	-0.052 (0.06)	0.42	-0.135 (0.08)	0.08	0.074 (0.07)	0.3
**ITWM**	0.111 (0.07)	0.13	-0.027 (0.06)	0.66	0.045 (0.07)	0.51	-0.092 (0.07)	0.21	-0.004 (0.07)	0.96
**ALIC**	0.046 (0.07)	0.52	0.06 (0.06)	0.31	-0.021 (0.07)	0.77	-0.098 (0.08)	0.19	0.063 (0.07)	0.34
**Tau**										
**GCC**	-0.01 (0.06)	0.86	0.007 (0.05)	0.89	0.022 (0.06)	0.71	-0.013 (0.06)	0.83	0.04 (0.05)	0.42
**BCC**	-0.057 (0.06)	0.32	-0.005 (0.05)	0.92	0.022 (0.06)	0.72	0.01 (0.06)	0.87	-0.005 (0.05)	0.93
**SCC**	0.023 (0.06)	0.68	0.002 (0.05)	0.97	-0.019 (0.06)	0.75	-0.011 (0.06)	0.85	0.021 (0.05)	0.65
**FX**	-0.002 (0.05)	0.97	0.044 (0.05)	0.38	0.08 (0.07)	0.24	-0.02 (0.07)	0.77	-0.061 (0.07)	0.36
**CGC**	-0.085 (0.06)	0.15	0.039 (0.05)	0.46	-0.048 (0.06)	0.39	0.01 (0.07)	0.88	0.062 (0.05)	0.23
**CGH**	-0.08 (0.06)	0.18	0.015 (0.06)	0.8	-0.018 (0.06)	0.76	0.01 (0.07)	0.88	0.059 (0.07)	0.38
**SLF**	-0.071 (0.06)	0.27	0.008 (0.05)	0.87	-0.033 (0.05)	0.52	0.078 (0.07)	0.24	0.007 (0.06)	0.9
**IFOF**	-0.105 (0.06)	0.1	0.069 (0.05)	0.19	-0.073 (0.06)	0.19	0.059 (0.07)	0.39	0.016 (0.06)	0.8
**ITWM**	-0.11 (0.06)	0.086	**0.134 (0.05)**	**0.014**	-0.109 (0.06)	0.07	-0.034 (0.07)	0.61	0.078 (0.06)	0.22
**ALIC**	-0.083 (0.06)	0.19	0.028 (0.05)	0.6	-0.027 (0.06)	0.67	0.031 (0.07)	0.64	0.001 (0.06)	0.99

For Review Only

Supplementary Table 2: Association of diffusion metrics with cognition after controlling for age, sex and education/occupation, cycle number, amyloid and tau. FA- fractional anisotropy, MD- mean diffusivity, NDI- neurite density, ODI- orientation dispersion index, ISOVF – isotropic volume fraction, GCC- genu of corpus callosum, BCC- body of corpus callosum, and SCC- splenium of corpus callosum, FX- fornix, CGC- cingulum, CGH- parahippocampal cingulum, SLF- superior longitudinal fasciculus, IFOF- inferior fronto-occipital fasciculus, ITWM- inferior temporal WM, and ALIC- anterior limb of internal capsule.

Variable	Regression Coefficient (SE)	p	Regression Coefficient (SE)	p	Regression Coefficient (SE)		Regression Coefficient (SE)	p	Regression Coefficient (SE)	p
	FA		MD		NDI		ODI		ISOVF	
Global Cognition										
GCC	**0.348 (0.06)**	**<0.001**	**-0.406 (0.07)**	**<0.001**	**0.214 (0.06)**	**<0.001**	-0.056 (0.06)	0.37	**-0.476 (0.08)**	**<0.001**
BCC	**0.308 (0.06)**	**<0.001**	**-0.41(0.07)**	**<0.001**	**0.21 (0.06)**	**<0.001**	-0.062 (0.06)	0.31	**-0.444 (0.06)**	**<0.001**
SCC	**0.363 (0.07)**	**<0.001**	**-0.437 (0.06)**	**<0.001**	**0.235 (0.06)**	**<0.001**	**0.136 (0.06)**	**0.035**	**-0.382 (0.08)**	**<0.001**
FX	**0.293 (0.07)**	**<0.001**	**-0.332 (0.07)**	**<0.001**	0.027 (0.06)	0.63	**-0.11 (0.06)**	**0.044**	0.039 (0.06)	0.49
CGC	**0.209 (0.06)**	**<0.001**	**-0.273 0.06**	**<0.001**	**0.236 (0.06)**	**<0.001**	**-0.168 (0.06)**	**0.003**	**-0.234 (0.07)**	**0.002**
CGH	**0.27 (0.06)**	**<0.001**	**-0.221(0.06)**	**<0.001**	**0.207 (0.06)**	**<0.001**	**-0.115 (0.06)**	**0.042**	0.04 (0.06)	0.49
SLF	**0.137 (0.06)**	**0.016**	**-0.302 (0.06)**	**<0.001**	**0.3 (0.06)**	**<0.001**	0.02 (0.06)	0.72	-0.019 (0.06)	0.76
IFOF	**0.224 (0.06)**	**<0.001**	**-0.324 (0.07)**	**<0.001**	**0.24 (0.06)**	**<0.001**	-0.057 (0.06)	0.31	**-0.151 (0.06)**	**0.014**
ITWM	**0.126 (0.06)**	**0.03**	**-0.332 (0.06)**	**<0.001**	**0.244 (0.06)**	**<0.001**	**0.153 (0.06)**	**0.009**	-0.001 (0.06)	0.99
ALIC	**0.193 (0.06)**	**<0.001**	**-0.242 (0.07)**	**<0.001**	0.107(0.06)	0.067	-0.029 (0.06)	0.6	**-0.191 (0.06)**	**0.002**

For Review Only

Supplementary Figures

Supplementary Figure 1: Association between **1A.** Amyloid and diffusion metrics (NDI, ISOVF and MD). **1B**. Tau and diffusion metrics (ISOVF and MD). Significance level set at $p<0.05$, FWE corrected (MD and ISOVF), and uncorrected $p<0.001$(NDI) with an extend threshold of K=100. FA- fractional anisotropy, MD- mean diffusivity, NDI- neurite density, ODI- orientation dispersion index, ISOVF – isotropic volume fraction.

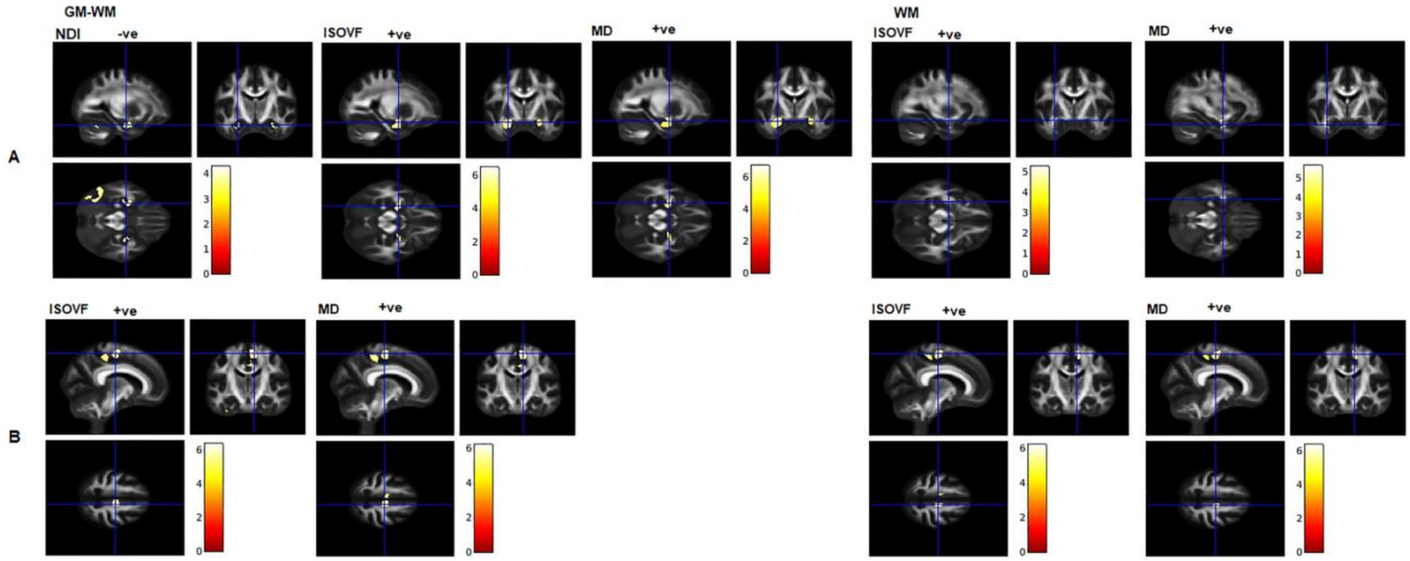

Supplementary Figure 2: Association between diffusion metrics with processing speed after controlling for age, sex and education/occupation, cycle visit, amyloid, and tau. Different symbols below are used for each of the diffusion measures. FA- fractional anisotropy, MD- mean diffusivity, NDI- neurite density, ODI- orientation dispersion index, ISOVF – isotropic volume fraction, GCC- genu of corpus callosum, BCC- body of corpus callosum, and SCC- splenium of corpus callosum.

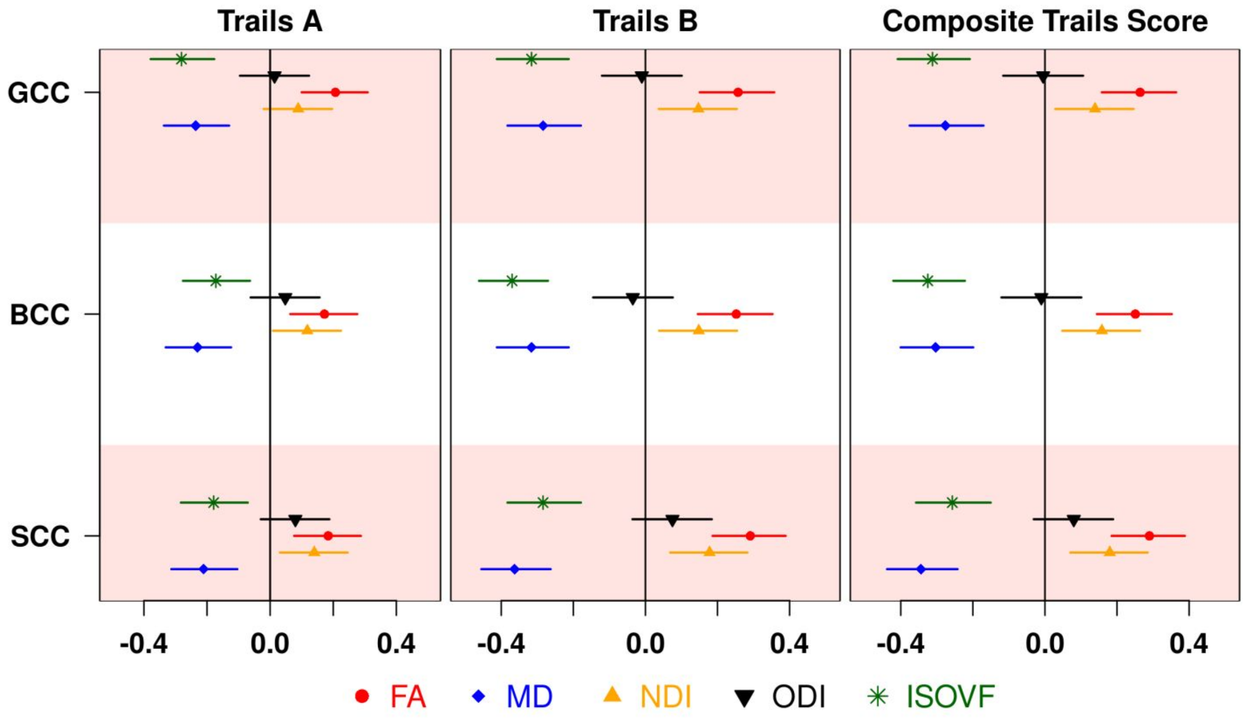

Supplement: fcab106_Supplementary_Data [file fcab106_supplementary_data.zip › Original Submission.pdf]
