# Supplementary material for: Diffusion models reveal white matter microstructural changes with ageing, pathology and cognition
Source: Brain Commun. 2021 May 19;3(2):fcab106. doi: 10.1093/braincomms/fcab106 (PMC8202149; doi:10.1093/braincomms/fcab106)

**ONLINE SUPPLEMENTARY MATERIALS**

**Diffusion models reveal white matter microstructural changes with aging, pathology, and cognition**

Sheelakumari Raghavan^1^, Robert I. Reid^2^, Scott A. Przybelski^3^, Timothy G. Lesnick^3^, Jonathan Graff-Radford^4^, Christopher G. Schwarz^1^, David S. Knopman^4^, Michelle M. Mielke^3,4^, Mary M. Machulda^5^, Ronald C. Petersen^4^, Clifford R. Jack Jr^1^, Prashanthi Vemuri^1^

^1^Departments of Radiology, Mayo Clinic, Rochester, MN, USA

^2^Information Technology, Mayo Clinic, Rochester, MN, USA

^3^Quantitative Health Sciences, Mayo Clinic, Rochester, MN, USA

^4^Neurology, Mayo Clinic Rochester, MN, USA

^5^Psychology, Mayo Clinic Rochester, MN, USA

**Corresponding Author**: Prashanthi Vemuri, Ph.D.

Mayo Clinic and Foundation

200 First Street SW, Rochester, MN 55905, USA

Phone: +1 507 538 0761, Fax:+1 507 284 9778, e-mail: [**vemuri.prashanthi@mayo.edu**](mailto:vemuri.prashanthi@mayo.edu)

**Supplementary Table 1:** Association between white matter hyper intensity (WMH) or amyloid or, tau and diffusion metrics after controlling for age, sex and education/occupation. FA - fractional anisotropy, MD - mean diffusivity, NDI - neurite density index, ODI - orientation dispersion index, ISOVF - isotropic volume fraction, GCC - genu of corpus callosum, BCC - body of corpus callosum, and SCC - splenium of corpus callosum, FX - fornix, CGC - cingulum, CGH - parahippocampal cingulum, SLF - superior longitudinal fasciculus, IFOF - inferior fronto-occipital fasciculus, ITWM - inferior temporal WM, and ALIC - anterior limb of internal capsule.

| **Variable** | **Regression Coefficient (SE)** | **p** | **Regression Coefficient (SE)** | **p** | **Regression Coefficient (SE)** | **p** | **Regression Coefficient (SE)** | **p** | **Regression Coefficient (SE)** | **p** |
| --- | --- | --- | --- | --- | --- | --- | --- | --- | --- | --- |
|  | **FA** |  | **MD** |  | **NDI** |  | **ODI** |  | **ISOVF** |  |
| **WMH** |  |  |  |  |  |  |  |  |  |  |
| **GCC** | **-0.479 (0.06)** | **<0.001** | **0.474 (0.05)** | **<0.001** | **-0.469 (0.06)** | **<0.001** | -0.03 (0.07) | 0.65 | **0.346 (0.06)** | **<0.001** |
| **BCC** | **-0.529 (0.06)** | **<0.001** | **0.569 (0.05)** | **<0.001** | **-0.511 (0.07)** | **<0.001** | -0.005 (0.07) | 0.95 | **0.447 (0.06)** | **<0.001** |
| **SCC** | **-0.547 (0.06)** | **<0.001** | **0.543 (0.06)** | **<0.001** | **-0.412 (0.07)** | **<0.001** | **-0.156 (0.06)** | **0.014** | **0.382 (0.05)** | **<0.001** |
| **FX** | **-0.264 (0.06)** | **<0.001** | **0.245 (0.06)** | **<0.001** | -0.106 (0.08) | 0.16 | 0.1 (0.07) | 0.18 | -0.007 (0.07) | 0.92 |
| **CGC** | **-0.263 (0.07)** | **<0.001** | **0.435 (0.06)** | **<0.001** | **-0.378 (0.06)** | **<0.001** | 0.114 (0.07) | 0.12 | **0.121 (0.06)** | **0.035** |
| **CGH** | **-0.263 (0.07)** | **<0.001** | **0.329 (0.06)** | **<0.001** | **-0.261 (0.08)** | **<0.001** | 0.049 (0.07) | 0.5 | 0.127 (0.07) | 0.082 |
| **SLF** | **-0.347 (0.07)** | **<0.001** | **0.649 (0.05)** | **<0.001** | **-0.621 (0.06)** | **<0.001** | -0.123 (0.07) | 0.1 | **0.342 (0.07)** | **<0.001** |
| **IFOF** | **-0.206 (0.07)** | **0.003** | **0.374 (0.06)** | **<0.001** | **-0.32 (0.06)** | **<0.001** | -0.056 (0.08) | 0.45 | **0.21 (0.07)** | **0.002** |
| **ITWM** | **-0.323 (0.07)** | **<0.001** | **0.461 (0.06)** | **<0.001** | **-0.428 (0.07)** | **<0.001** | -0.116 (0.07) | 0.11 | 0.005 (0.07) | 0.94 |
| **ALIC** | **-0.378 (0.07)** | **<0.001** | **0.466 (0.06)** | **<0.001** | **-0.275 (0.07)** | **<0.001** | 0.057 (0.07) | 0.44 | **0.426 (0.06)** | **<0.001** |
| **Amyloid** |  |  |  |  |  |  |  |  |  |  |
| **GCC** | 0.061 (0.06) | 0.33 | 0.057 (0.06) | 0.3 | 0.016 (0.07) | 0.8 | -0.043 (0.07) | 0.54 | 0.099 (0.06) | 0.083 |
| **BCC** | 0.068 (0.07) | 0.31 | 0.05 (0.05) | 0.34 | -0.015 (0.07) | 0.82 | -0.098 (0.07) | 0.16 | 0.093 (0.06) | 0.12 |
| **SCC** | 0.038 (0.06) | 0.54 | 0.022 (0.06) | 0.7 | 0.041 (0.07) | 0.54 | -0.08 (0.07) | 0.22 | 0.069 (0.05) | 0.19 |
| **FX** | -0.114 (0.06) | 0.058 | 0.072 (0.06) | 0.21 | -0.096 (0.08) | 0.22 | 0.022 (0.08) | 0.77 | 0.138 (0.08) | 0.071 |
| **CGC** | 0.007 (0.07) | 0.91 | 0.054 (0.06) | 0.37 | -0.053 (0.06) | 0.4 | 0.042 (0.08) | 0.58 | 0.018 (0.06) | 0.76 |
| **CGH** | -0.066 (0.07) | 0.34 | **0.148 (0.07)** | **0.026** | -0.134 (0.07) | 0.053 | -0.028 (0.08) | 0.71 | -0.042 (0.08) | 0.58 |
| **SLF** | 0.105 (0.07) | 0.15 | 0.007 (0.06) | 0.9 | -0.002 (0.06) | 0.97 | -0.11 (0.08) | 0.15 | -0.064 (0.07) | 0.35 |
| **IFOF** | 0.029 (0.07) | 0.69 | 0.077 (0.06) | 0.19 | -0.052 (0.06) | 0.42 | -0.135 (0.08) | 0.08 | 0.074 (0.07) | 0.3 |
| **ITWM** | 0.111 (0.07) | 0.13 | -0.027 (0.06) | 0.66 | 0.045 (0.07) | 0.51 | -0.092 (0.07) | 0.21 | -0.004 (0.07) | 0.96 |
| **ALIC** | 0.046 (0.07) | 0.52 | 0.06 (0.06) | 0.31 | -0.021 (0.07) | 0.77 | -0.098 (0.08) | 0.19 | 0.063 (0.07) | 0.34 |
| **Tau** |  |  |  |  |  |  |  |  |  |  |
| **GCC** | -0.01 (0.06) | 0.86 | 0.007 (0.05) | 0.89 | 0.022 (0.06) | 0.71 | -0.013 (0.06) | 0.83 | 0.04 (0.05) | 0.42 |
| **BCC** | -0.057 (0.06) | 0.32 | -0.005 (0.05) | 0.92 | 0.022 (0.06) | 0.72 | 0.01 (0.06) | 0.87 | -0.005 (0.05) | 0.93 |
| **SCC** | 0.023 (0.06) | 0.68 | 0.002 (0.05) | 0.97 | -0.019 (0.06) | 0.75 | -0.011 (0.06) | 0.85 | 0.021 (0.05) | 0.65 |
| **FX** | -0.002 (0.05) | 0.97 | 0.044 (0.05) | 0.38 | 0.08 (0.07) | 0.24 | -0.02 (0.07) | 0.77 | -0.061 (0.07) | 0.36 |
| **CGC** | -0.085 (0.06) | 0.15 | 0.039 (0.05) | 0.46 | -0.048 (0.06) | 0.39 | 0.01 (0.07) | 0.88 | 0.062 (0.05) | 0.23 |
| **CGH** | -0.08 (0.06) | 0.18 | 0.015 (0.06) | 0.8 | -0.018 (0.06) | 0.76 | 0.01 (0.07) | 0.88 | 0.059 (0.07) | 0.38 |
| **SLF** | -0.071 (0.06) | 0.27 | 0.008 (0.05) | 0.87 | -0.033 (0.05) | 0.52 | 0.078 (0.07) | 0.24 | 0.007 (0.06) | 0.9 |
| **IFOF** | -0.105 (0.06) | 0.1 | 0.069 (0.05) | 0.19 | -0.073 (0.06) | 0.19 | 0.059 (0.07) | 0.39 | 0.016 (0.06) | 0.8 |
| **ITWM** | -0.11 (0.06) | 0.086 | **0.134 (0.05)** | **0.014** | -0.109 (0.06) | 0.07 | -0.034 (0.07) | 0.61 | 0.078 (0.06) | 0.22 |
| **ALIC** | -0.083 (0.06) | 0.19 | 0.028 (0.05) | 0.6 | -0.027 (0.06) | 0.67 | 0.031 (0.07) | 0.64 | 0.001 (0.06) | 0.99 |

**Supplementary Table 2:** Association of diffusion metrics with cognition after controlling for age, sex and education/occupation, cycle number, amyloid and tau. FA - fractional anisotropy, MD - mean diffusivity, NDI - neurite density index, ODI - orientation dispersion index, ISOVF - isotropic volume fraction, GCC - genu of corpus callosum, BCC - body of corpus callosum, and SCC - splenium of corpus callosum, FX - fornix, CGC - cingulum, CGH - parahippocampal cingulum, SLF - superior longitudinal fasciculus, IFOF - inferior fronto-occipital fasciculus, ITWM - inferior temporal WM, and ALIC - anterior limb of internal capsule.

| **Variable** | **Regression Coefficient (SE)** | **p** | **Regression Coefficient (SE)** | **p** | **Regression Coefficient (SE)** |  | **Regression Coefficient (SE)** | **p** | **Regression Coefficient (SE)** | **p** |
| --- | --- | --- | --- | --- | --- | --- | --- | --- | --- | --- |
|  | **FA** |  | **MD** |  | **NDI** |  | **ODI** |  | **ISOVF** |  |
| **Global Cognition** |  |  |  |  |  |  |  |  |  |  |
| **GCC** | **0.348 (0.06)** | **<0.001** | **-0.406 (0.07)** | **<0.001** | **0.214 (0.06)** | **<0.001** | -0.056 (0.06) | 0.37 | **-0.476 (0.08)** | **<0.001** |
| **BCC** | **0.308 (0.06)** | **<0.001** | **-0.41(0.07)** | **<0.001** | **0.21 (0.06)** | **<0.001** | -0.062 (0.06) | 0.31 | **-0.444 (0.06)** | **<0.001** |
| **SCC** | **0.363 (0.07)** | **<0.001** | **-0.437 (0.06)** | **<0.001** | **0.235 (0.06)** | **<0.001** | **0.136 (0.06)** | **0.035** | **-0.382 (0.08)** | **<0.001** |
| **FX** | **0.293 (0.07)** | **<0.001** | **-0.332 (0.07)** | **<0.001** | 0.027 (0.06) | 0.63 | **-0.11 (0.06)** | **0.044** | 0.039 (0.06) | 0.49 |
| **CGC** | **0.209 (0.06)** | **<0.001** | **-0.273 0.06** | **<0.001** | **0.236 (0.06)** | **<0.001** | **-0.168 (0.06)** | **0.003** | **-0.234 (0.07)** | **0.002** |
| **CGH** | **0.27 (0.06)** | **<0.001** | **-0.221(0.06)** | **<0.001** | **0.207 (0.06)** | **<0.001** | **-0.115 (0.06)** | **0.042** | 0.04 (0.06) | 0.49 |
| **SLF** | **0.137 (0.06)** | **0.016** | **-0.302 (0.06)** | **<0.001** | **0.3 (0.06)** | **<0.001** | 0.02 (0.06) | 0.72 | -0.019 (0.06) | 0.76 |
| **I**FO**F** | **0.224 (0.06)** | **<0.001** | **-0.324 (0.07)** | **<0.001** | **0.24 (0.06)** | **<0.001** | -0.057 (0.06) | 0.31 | **-0.151 (0.06)** | **0.014** |
| **ITWM** | **0.126 (0.06)** | **0.03** | **-0.332 (0.06)** | **<0.001** | **0.244 (0.06)** | **<0.001** | **0.153 (0.06)** | **0.009** | -0.001 (0.06) | 0.99 |
| **ALIC** | **0.193 (0.06)** | **<0.001** | **-0.242 (0.07)** | **<0.001** | 0.107(0.06) | 0.067 | -0.029 (0.06) | 0.6 | **-0.191 (0.06)** | **0.002** |

**Supplementary Figures**

**Supplementary Figure 1: A).** Association between amyloid and diffusion metrics (NDI, ISOVF and MD) and **B)**. Association between tau and diffusion metrics (ISOVF and MD). Significance level set at p<0.05, FWE corrected (MD and ISOVF), and uncorrected p<0.001(NDI) with an extend threshold of K=100. FA - fractional anisotropy, MD - mean diffusivity, NDI - neurite density index, ODI - orientation dispersion index, ISOVF - isotropic volume fraction.


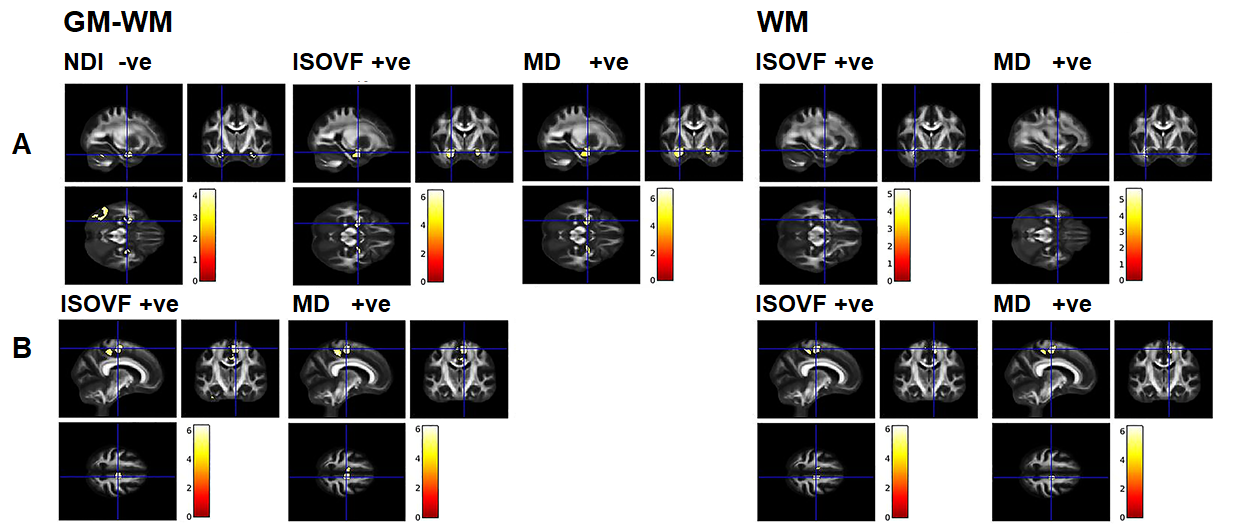


**Supplementary Figure 2:** Association between diffusion metrics with processing speed after controlling for age, sex, education/occupation, cycle visit, amyloid, and tau. Different symbols below are used for each of the diffusion measures. FA - fractional anisotropy, MD - mean diffusivity, NDI - neurite density index, ODI - orientation dispersion index, ISOVF - isotropic volume fraction, GCC - genu of corpus callosum, BCC - body of corpus callosum, and SCC - splenium of corpus callosum.


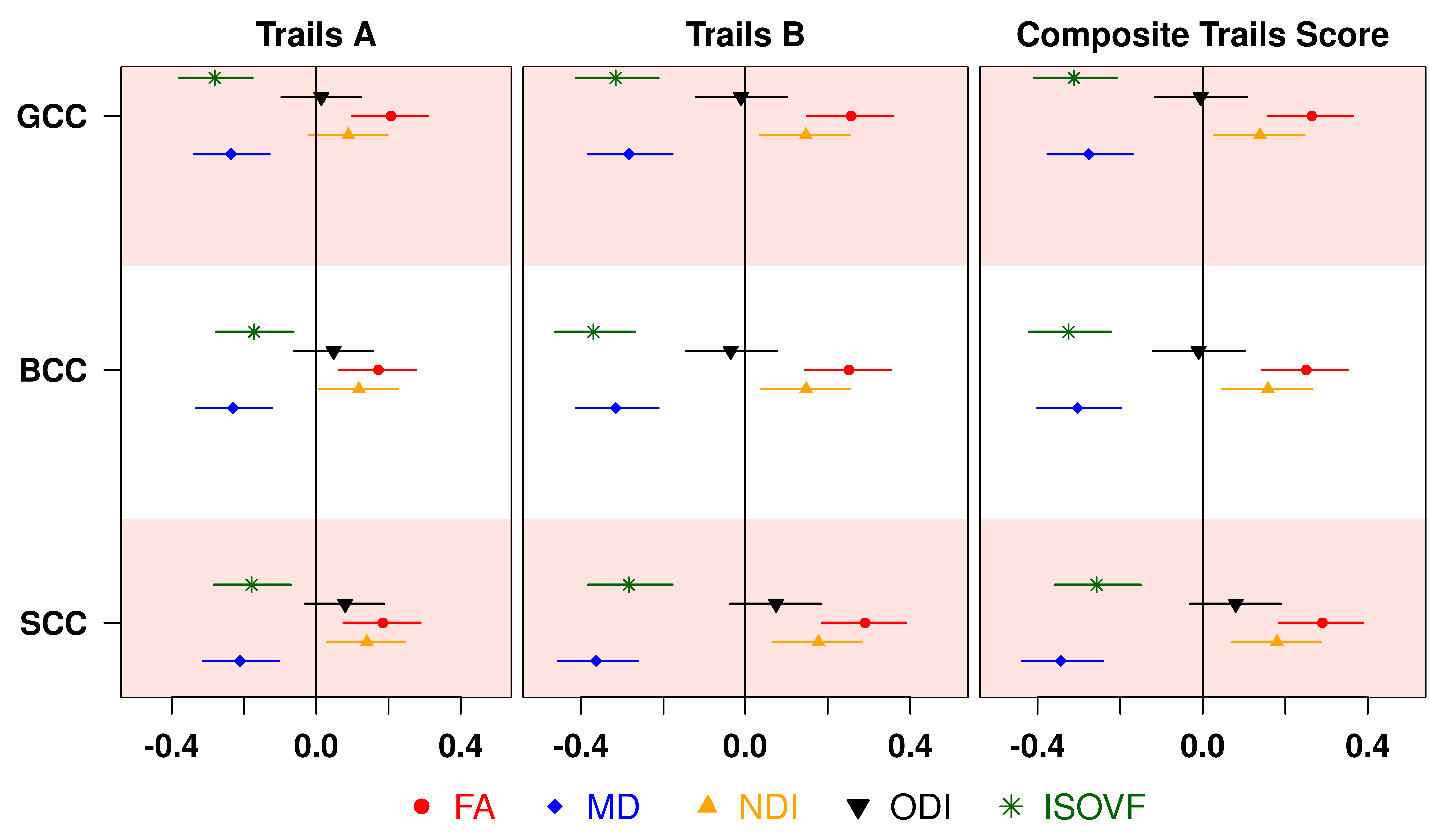


**Supplementary Figure 3:** Association of diffusion metrics with white matter hyperintensity (WMH), amyloid, and tau after controlling for age, sex, and education/occupation in non-demented participants. Different symbols below are used for each of the primary predictors. FA - fractional anisotropy, MD - mean diffusivity, NDI - neurite density index, ODI - orientation dispersion index, ISOVF - isotropic volume fraction.


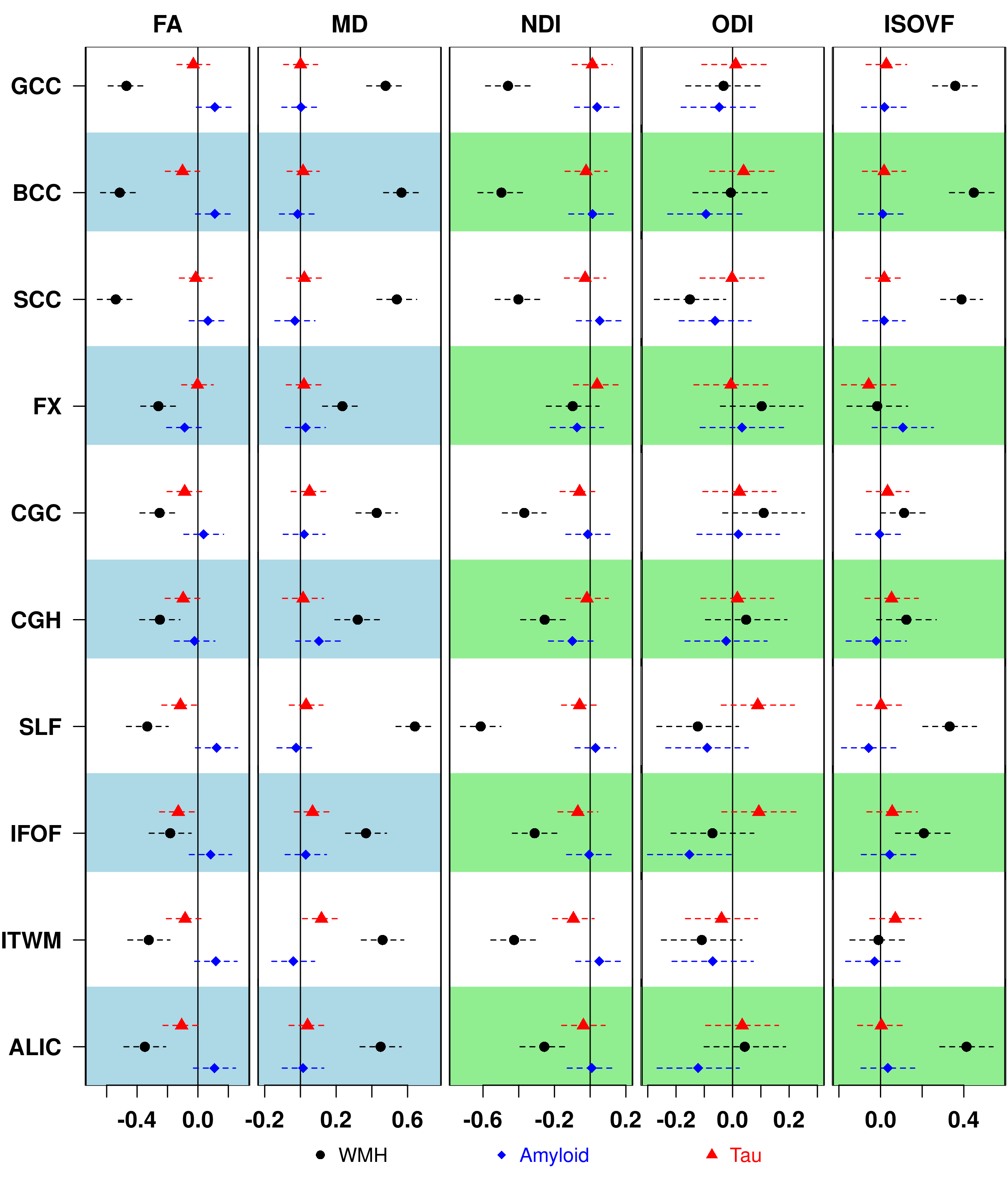


**Supplementary Figure 4:** Association of diffusion metrics with cognition after controlling for age, sex, education/occupation, cycle visit, amyloid, and tau in non-demented participants. Different symbols below are used for each of the diffusion measures. FA - fractional anisotropy, MD - mean diffusivity, NDI - neurite density index, ODI - orientation dispersion index, ISOVF - isotropic volume fraction, GCC - genu of corpus callosum, BCC - body of corpus callosum, and SCC - splenium of corpus callosum.


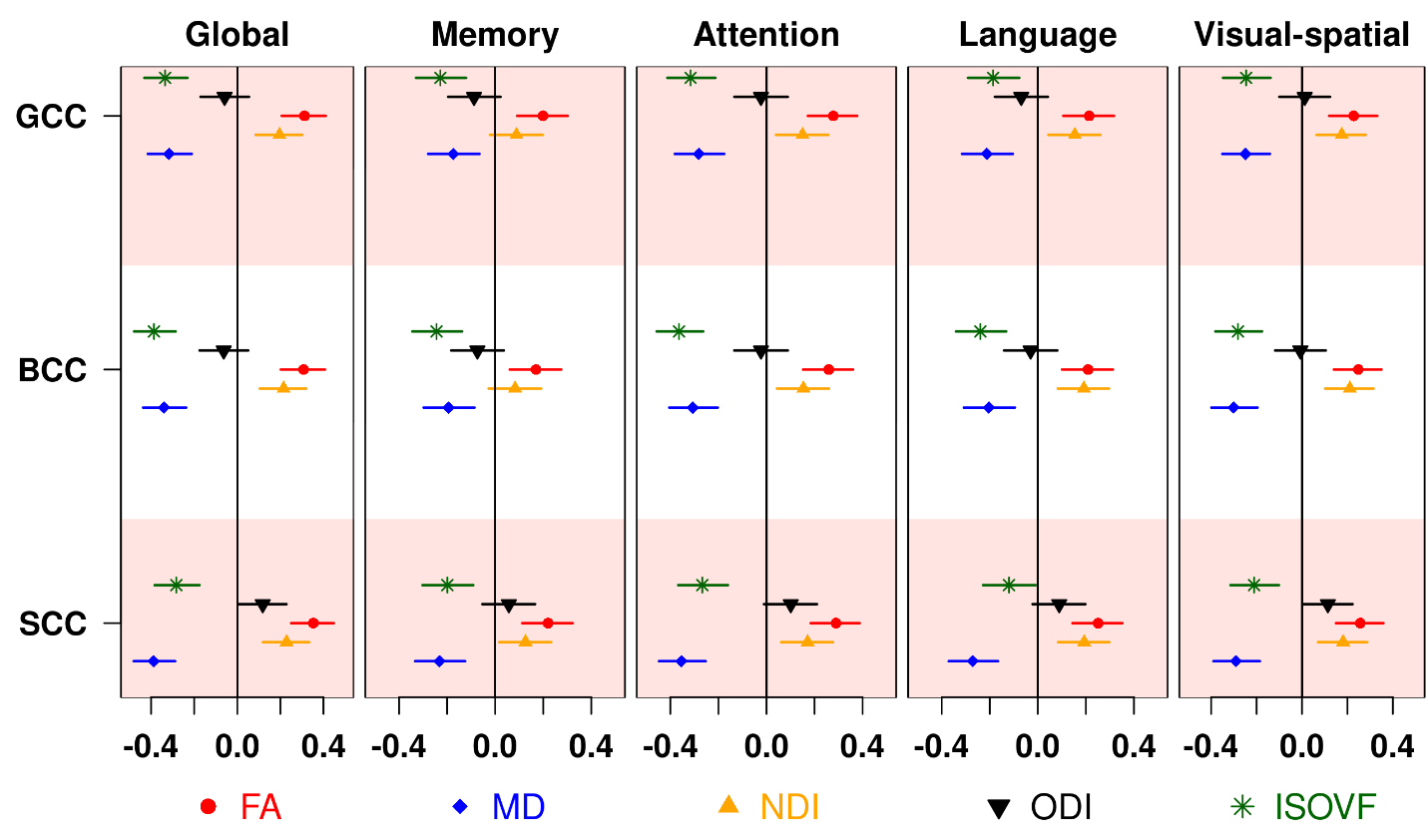


**Supplementary Figure 5:** Association between diffusion metrics with processing speed after controlling for age, sex, education/occupation, cycle visit, amyloid, and tau in non-demented participants. Different symbols below are used for each of the diffusion measures. FA - fractional anisotropy, MD - mean diffusivity, NDI - neurite density index, ODI - orientation dispersion index, ISOVF - isotropic volume fraction, GCC - genu of corpus callosum, BCC - body of corpus callosum, and SCC - splenium of corpus callosum.


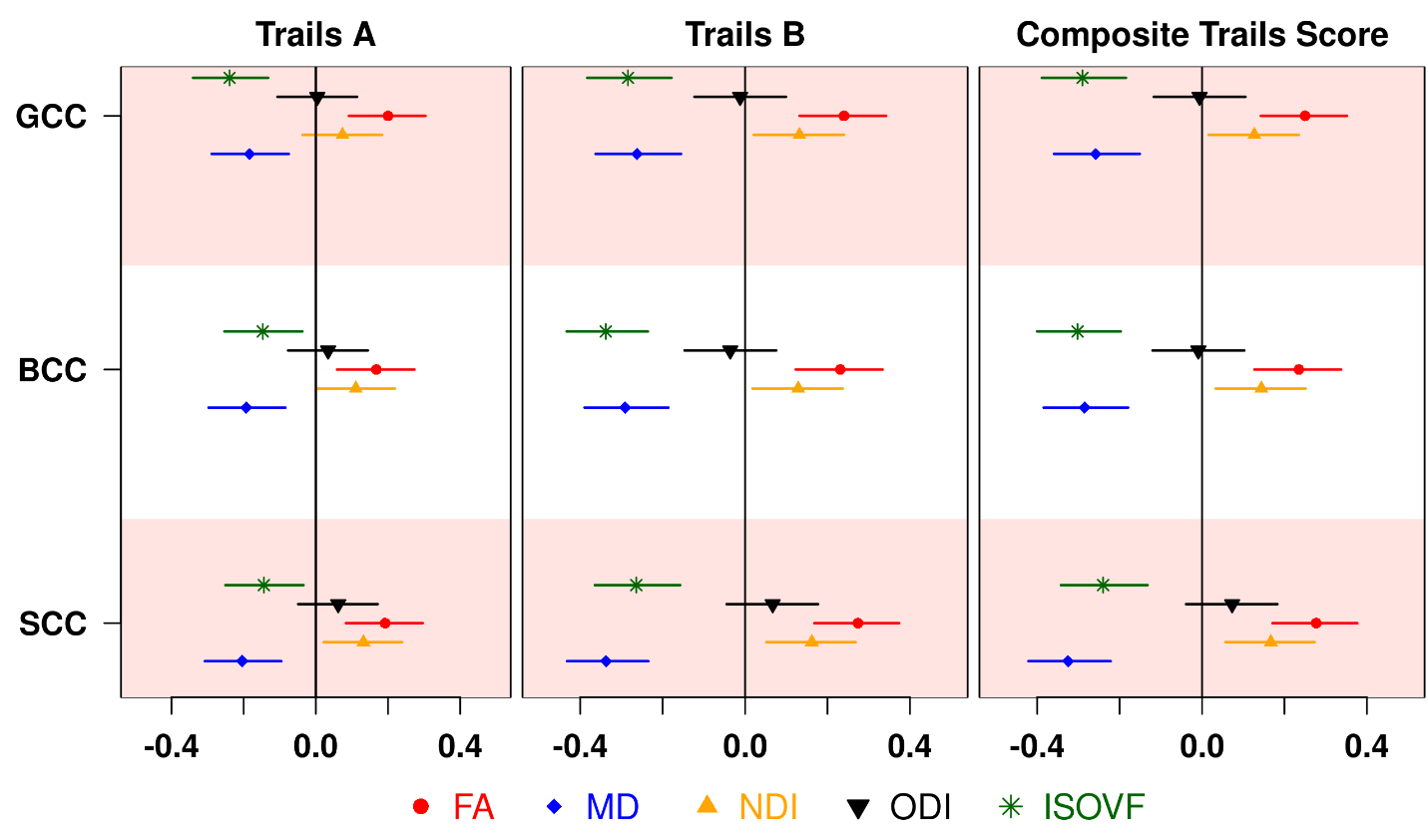

Supplement: fcab106_Supplementary_Data [file fcab106_supplementary_data.zip › Supplementary_material.docx]
