# Supplementary material for: Diffusion models reveal white matter microstructural changes with ageing, pathology and cognition
Source: Brain Commun. 2021 May 19;3(2):fcab106. doi: 10.1093/braincomms/fcab106 (PMC8202149; doi:10.1093/braincomms/fcab106)
Supplement: fcab106_Supplementary_Data [file Original Submission Response_to_reviewer.pdf › Original Submission Response_to_reviewer.pdf]

**<u>Response to reviewer comments:</u>**

***We would like to thank the reviewers and the editor for their suggestions. We believe that addressing the comments has strengthened the manuscript and help make our case more clearly. Changes made to the manuscript in response to the reviewer comments are highlighted in the manuscript in yellow. What follows is a point by point accounting of each comment by the reviewers.***

Reviewer: 1

Comments to the Author
Review BRAINCOM-2021-065:
Diffusion models reveal white matter microstructural changes with aging, pathology, and cognition

The multimodal neuroimaging study investigates microstructural alterations due to demographics, common age-related pathological processes, and cognition by advanced diffusion MRI analysis, i.e., Neurite Orientation Dispersion and Density Imaging (NODDI). Results were supplemented by amyloid and tau PET scans. The results is that all diffusion measures provided information about WM aging and WM disease processes and were associated with cognition. NODDI and DTI are two different biophysical models that provide distinct information about variation in WM microstructural integrity. The authors conclude that these findings suggest that traditional FA and MD measures perform well in detecting disease-specific pathologies and cognitive effects and claim that NODDI provides additional information which may aid in providing mechanistic insights into disease progression.
This is a very good study, the topic is up to date, analysis is straightforward and well performed, statistics are sound.

***Response: We thank the reviewer for the positive comments.***

Major concern:
1. The study includes a high number of subjects, however, the number of subjects with dementia (N=7) is far too low for any valid statement.
The authors should increase the number of dementia patients substantially (about 50 or a number based on effect size calculations) or omit this disease group from their analysis. Then, they have to tone down their statements regarding the mental development.

**Response:** ***We agree with the reviewer that the number of participants in the dementia group is lower. We addressed this as a limitation in the revised manuscript. Even though we have included the entire cohort in the paper, we have included the modified figures (without demented participants) as supplemental material. There were no significant changes observed with removal of demented individuals. Please find the newly added supplemental material below.***

**Supplementary Figure 3.** Association of diffusion metrics with white matter hyperintensity (WMH), amyloid, and tau after controlling for age, sex, and education/occupation in non-demented participants. Different symbols below are used for each of the primary predictors. FA- fractional anisotropy, MD- mean diffusivity, NDI- neurite density, ODI- orientation dispersion index, ISOVF – isotropic volume fraction.

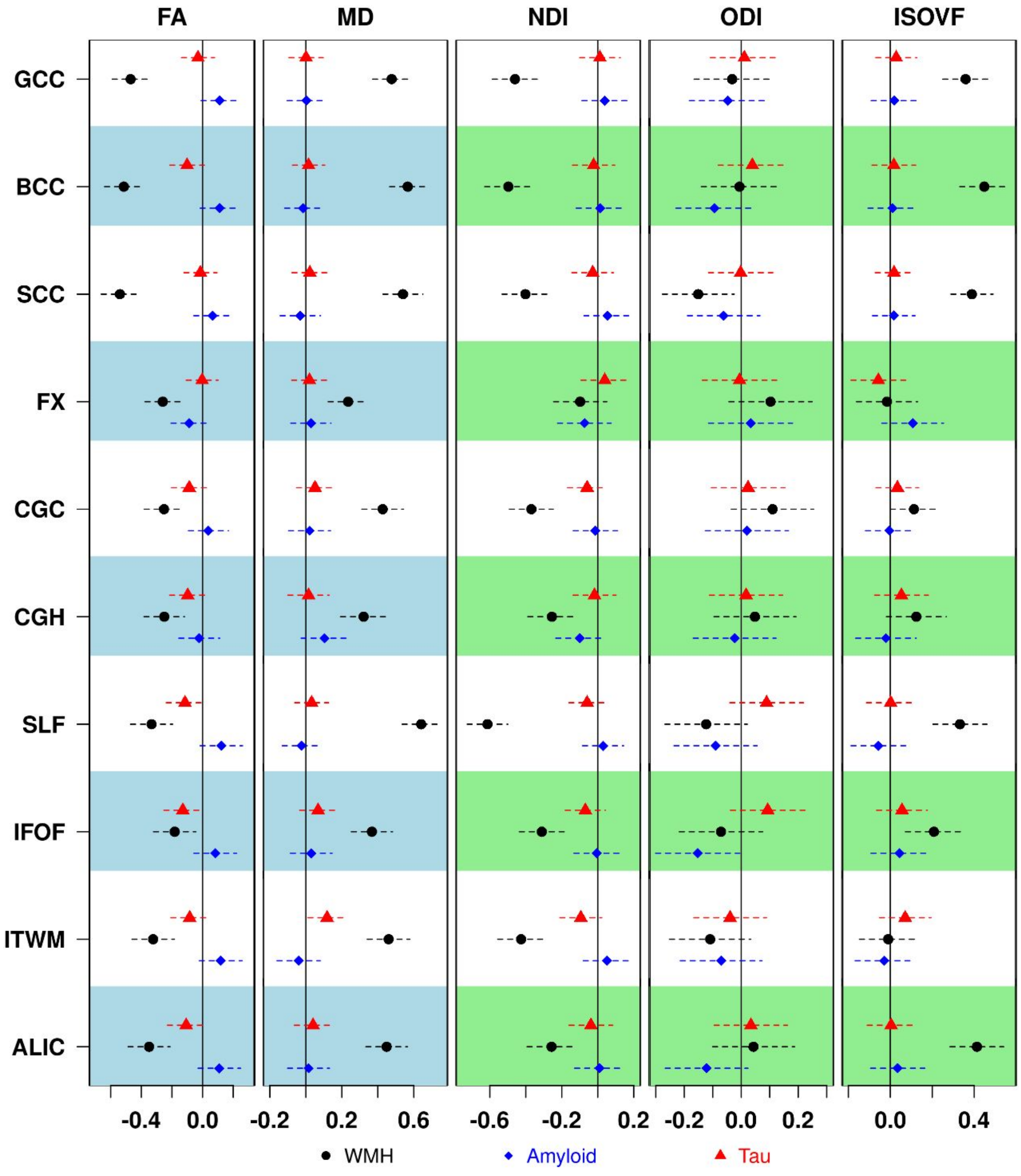

**Supplementary Figure 4**: Association of diffusion metrics with cognition after controlling for age, sex and education/occupation, cycle visit, amyloid, and tau in non-demented participants. Different symbols below are used for each of the diffusion measures. FA- fractional anisotropy, MD- mean diffusivity, NDI- neurite density, ODI- orientation dispersion index, ISOVF – isotropic volume fraction, GCC- genu of corpus callosum, BCC- body of corpus callosum, and SCC- splenium of corpus callosum

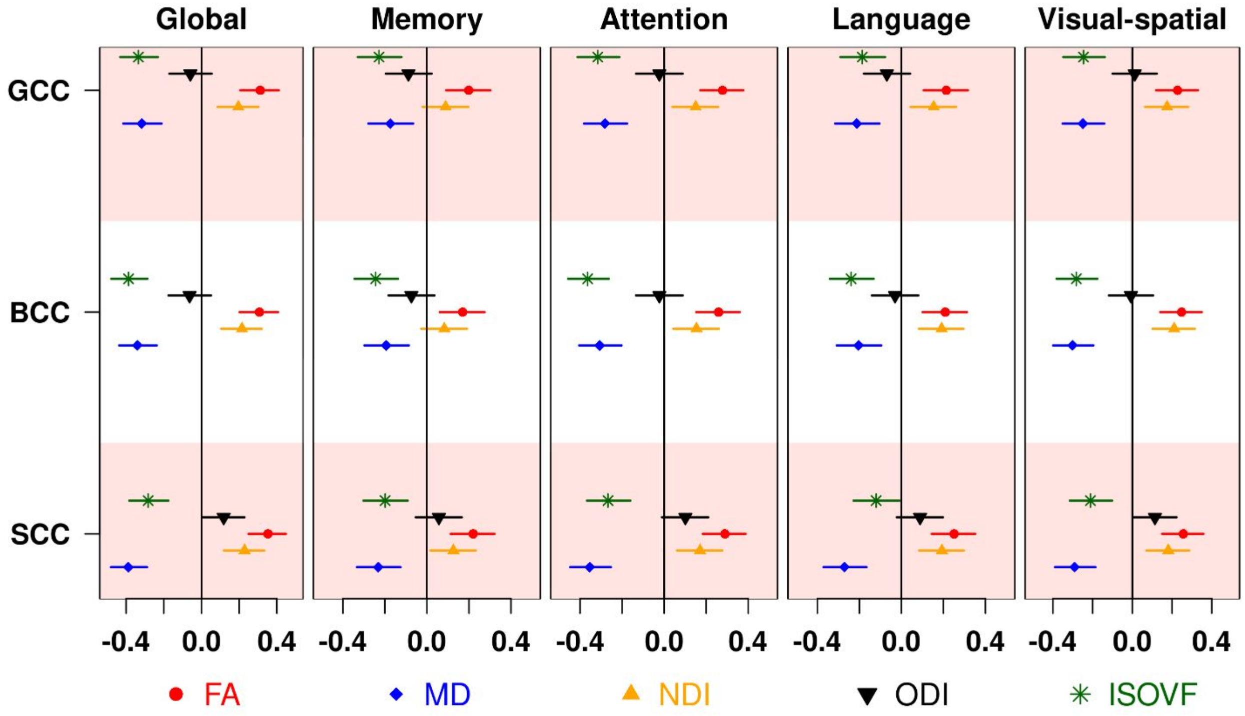

**Supplementary Figure 5**: Association between diffusion metrics with processing speed after controlling for age, sex and education/occupation, cycle visit, amyloid, and tau in non-demented participants. Different symbols below are used for each of the diffusion measures. FA- fractional anisotropy, MD- mean diffusivity, NDI- neurite density, ODI- orientation dispersion index, ISOVF – isotropic volume fraction, GCC- genu of corpus callosum, BCC- body of corpus callosum, and SCC- splenium of corpus callosum.

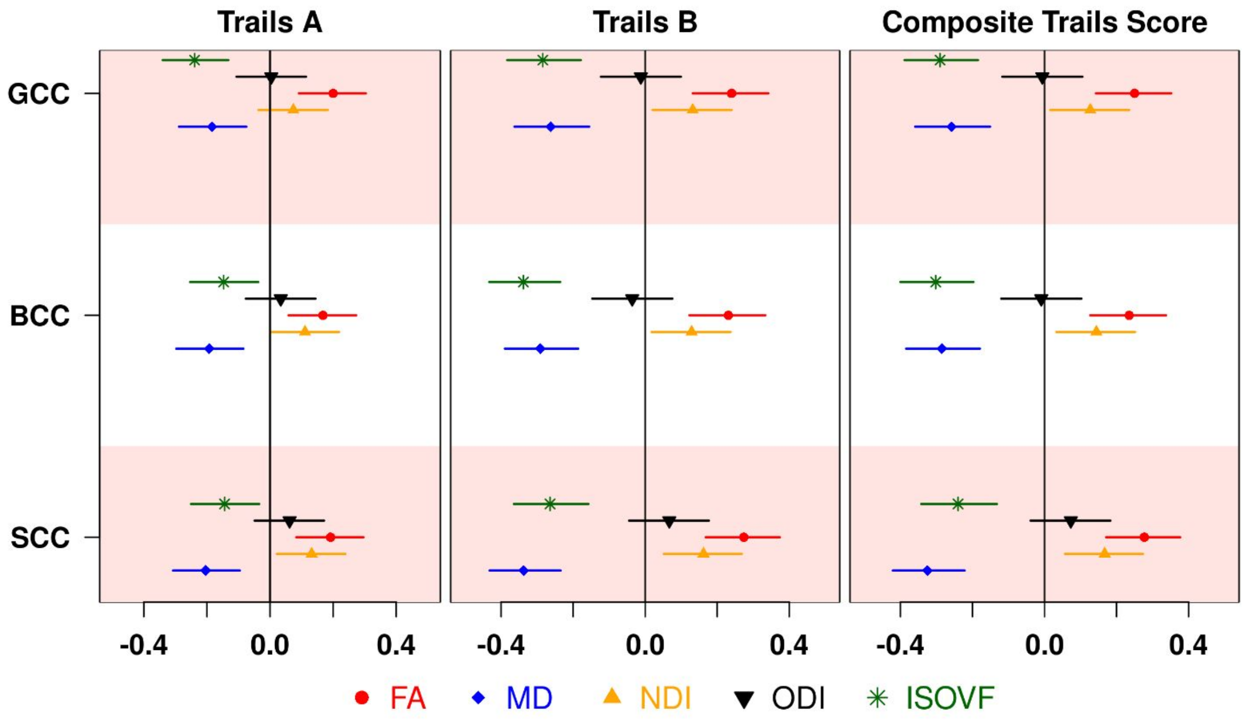


2. In addition, what do the authors mean by „disease-specific pathologies“ in their conclusions. They have only investigated healthy and MCI subjects (and an insufficiently sized dementia group). Please rephrase / clarify.

**Response: We agree with that the wording was very unclear and have clarified this point to say " …. All diffusion measures provided information about WM aging and WM changes due to age-related pathological disease processes…." to the conclusion of the abstract. We have also rephrased the conclusion of the paper.**

Minor:
Figure 1: The demographic data of the „representative subject“ should be given.

***Response: We have now included that the image is from a 67-year-old cognitively unimpaired female participant. We have added this to the revised manuscript in the Figure 1. Legend***

Figure 2 would benefit from an abbreviation legend as included in Figure 3.

***Response: We agree with this suggestion and have added the abbreviation legend in Figure 2***

The manuscript is apparently over-referenced.

***Response: The reviewer is quite correct, and we have tried to reduce some of the references from the discussion without losing the main message.***

Reviewer: 2

Comments to the Author
This is a well-written article investigating NODDI-derived white matter microstructural changes related to age, AD and CVD. The authors provide a thorough assessment of MRI, PET and cognitive metrics and the methods are appropriate and clearly explained. I have a few minor comments which may improve the manuscript slightly:

- A bit more detail could be provided about the participants, particularly the diagnosis of MCI and dementia. Were the diagnoses provided at the same time as the imaging assessment? If they were separate visits, what was the time interval? Both MCI and dementia are pretty heterogeneous conditions. Were there any post-mortem confirmations of particular diagnoses of e.g. AD? Were the MCI and dementia participants tau and/or amyloid positive?

***Response: Yes, the clinical visit at which diagnosis was made was at the same visit as the imaging assessments. In MCSA, almost all clinical and imaging visits are within 60-70 days (in this work the median was 63 days with a range of 0-124 days). There were only 4 autopsied individuals: 2 MCI, 1 AD and 1 DLB which are not sufficient to conduct any extensive analyses. The group amyloid and tau positivity break-down in the cohort were as follows -***
***CU A-T- n=171, CU A-T+ n=23, CU A+T- n=41 and CU A+T+ n=29,***
***MCI A-T- n=16, MCI A-T+ n=3, MCI A+T- n=12 and MCI A+T+ n=26***
***Demented A+T- n=1 and Demented A+T+ n=6***

- Is it possible to include additional columns for Table 1, separating out the demographic characteristics into MCI and dementia too rather than just the group as a whole?
- Participants scanned on one of two PRISMA scanners. Were they balanced across the groups? Even though the same parameters were selected it is still always important to check that there aren't any systematic differences between the groups which could be explained by individual scanners.

***Response: We really appreciate this comment and have added additional columns to Table 1 as shown below.***

**Table 1**. Characteristics table with the mean (SD) listed for the continuous variables and count (%) for the categorical variables.

| | CU n = 264 | MCI n = 57 | Dementia n = 7 | P-value |
|---|---|---|---|---|
| Male, no. (%) | 135 (51%) | 30 (53%) | 4 (57%) | 0.94 |
| Age, yrs | 65.9 (12.8) | 77.2 (9.8) | 84.7 (4.2) | <0.001 |
| E4 Carrier, no. (%) | 63 (27%) | 21 (40%) | 5 (71%) | 0.010 |
| Education/Occupation | 12.9 (2.2) | 11.9 (2.8) | 12.1 (2.3) | 0.013 |
| CMC | 1.5 (1.4) | 2.6 (1.8) | 3.0 (1.0) | <0.001 |
| MMSE | 29.0 (1.1) | 26.1 (1.9) | 20.3 (3.9) | <0.001 |
| zGlobal | 0.63 (0.99) | -1.80 (1.17) | -3.05 (0.49) | <0.001 |
| zMemory | 0.64 (0.96) | -1.85 (0.92) | -3.04 (0.15) | <0.001 |
| zAttention | 0.41 (1.02) | -1.50 (1.57) | -3.24 (0.78) | <0.001 |
| zLanguage | 0.35 (0.99) | -1.48 (1.38) | -2.96 (1.32) | <0.001 |
| zVisual-spatial | 0.56 (0.99) | -1.05 (1.23) | -1.87 (1.36) | <0.001 |
| Trails A | 32.0 (11.7) | 51.4 (24.9) | 91.5 (51.3) | <0.001 |
| Trails B | 75.6 (40.0) | 163.5 (96.2) | 262.3 (93.0) | <0.001 |
| Amyloid, SUVr | 1.49 (0.34) | 1.95 (0.62) | 3.04 (0.41) | <0.001 |
| Amyloid Positive, no. (%) | 70 (27%) | 38 (67%) | 7 (100%) | <0.001 |
| Tau, SUVr | 1.18 (0.11) | 1.29 (0.21) | 1.54 (0.30) | <0.001 |
| Tau Positive, no. (%) | 52 (20%) | 29 (51%) | 6 (86%) | <0.001 |
| WMH percentage | 0.66 (0.88) | 1.22 (0.93) | 1.98 (1.57) | <0.001 |

The p-values reported are from an ANOVA for continuous measurements and a chi-squared for categorical variables.

***We also repeated main analyses in Fig. 4 and Fig. 6 using scanner as a covariate and found no differences in results given the identical scanner settings on both PRISMA's and scan parameters. The additional analyses are shown here.***

Figure 4: Association of diffusion metrics with white matter hyperintensity (WMH), amyloid, and tau after controlling for age, sex, and education/occupation and type of scanner. FA- fractional anisotropy, MD- mean diffusivity, NDI- neurite density, ODI-

orientation dispersion index, ISOVF – isotropic volume fraction, GCC- genu of corpus callosum, BCC- body of corpus callosum, and SCC- splenium of corpus callosum

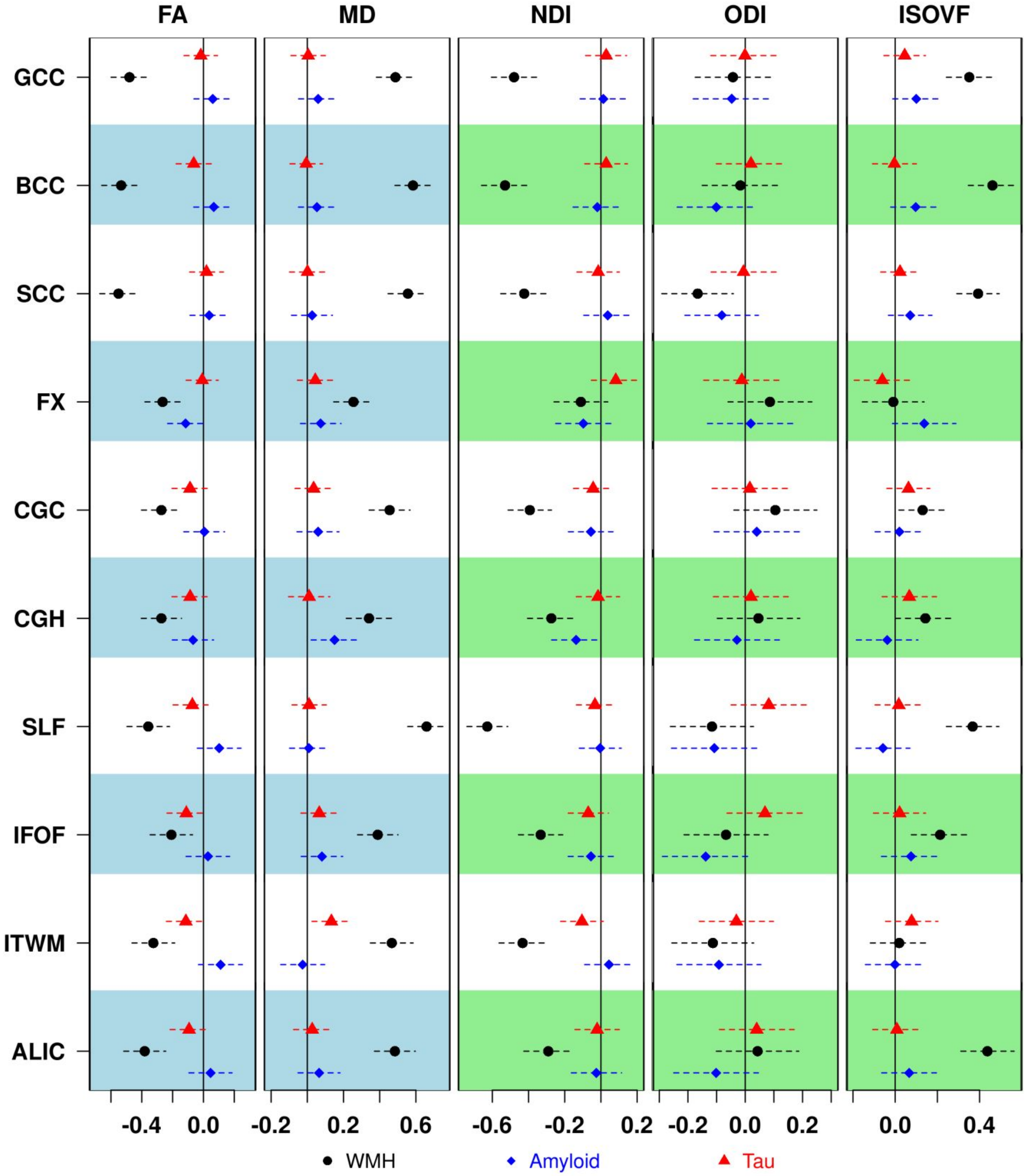


- The number of dementia patients is very small – I think this should be acknowledged as a limitation in that section of the discussion.

**Figure 6**: Association of diffusion metrics with cognition after controlling for age, sex education/occupation, type of scanner, cycle visit, amyloid, and tau. Different symbols below are used for each of the diffusion measures. FA- fractional anisotropy, MD- mean diffusivity, NDI- neurite density, ODI- orientation dispersion index, ISOVF – isotropic volume fraction, GCC- genu of corpus callosum, BCC- body of corpus callosum, and SCC- splenium of corpus callosum

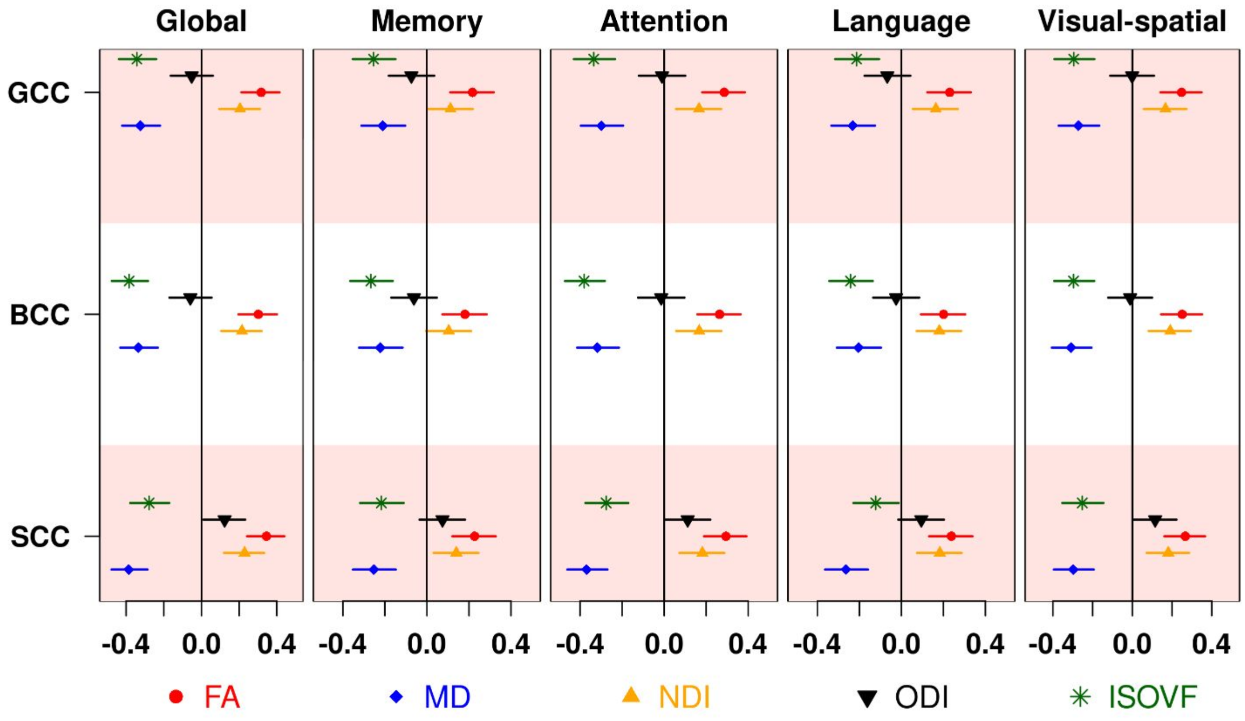


***Response: We agree and have added it as a limitation. We also repeated the analyses without demented individuals, and they are provided as supplemental material.***

- There is a typo on p9 'NOODDI' should be 'NODDI'

***Response: We have corrected the typo in the revised manuscript.***

Editorial Office comments:

-Please supply a graphical abstract. A graphical abstract is a visual representation of the central finding or methodology of the paper. It will be displayed in the table of contents. Authors should strive to make the graphical abstract informative, interesting, visually appealing, and straightforward (no caption needed, no abbreviations except gene names). Please upload as a separate high resolution image file (tiff preferred). The technical requirements for the graphical abstracts are:

--size of the submitted image: 1200 pixels square at 300dpi
--font of the text: Arial 12-16 point (smaller fonts will not be legible online)

***Response: We have added the graphical abstract.***

-Please add an Abbreviated Summary (up to 50 words) of your paper that captures the main purpose and conclusions of the work. The summary should state the main results written in the third person ('[1st author surname] et al. report that …') and conclusion. This summary will be used in the contents list online and should not contain any abbreviations other than those on the accepted abbreviations list and accepted gene/protein names, making it accessible to the non-specialist. Please upload it as a Word document. Allowed abbreviations: AIDS; ANOVA; AMPA; ATP; A,T,C,G; CNS; CSF; CT; DNA; ECG; EEG; EMG; GABA; HIV; MRI; NMDA; PET; PCR; RNA.

***Response***: ***We are adding the abbreviated summary here:***

***Raghavan et. al. report that advanced diffusion MRI can provide specific information about synaptic density, organization, and free water content of white matter microstructure. Age and WMH but not Alzheimer's disease are key drivers of these microstructural changes. Corpus callosum white matter health is fundamental for better cognitive performance.***

-Please include a paragraph about data availability in the 'Materials and Methods' section. We strongly encourage data sharing either as supplementary information or uploads to repositories which you can link in the paper.

***Response***: ***We have added the data availability statement in the revised manuscript.***
