# Supplementary material for: Diffusion models reveal white matter microstructural changes with ageing, pathology and cognition
Source: Brain Commun. 2021 May 19;3(2):fcab106. doi: 10.1093/braincomms/fcab106 (PMC8202149; doi:10.1093/braincomms/fcab106)
Supplement: fcab106_Supplementary_Data [file Original_Submission_Decision_Letter.pdf › Original_Submission_Decision_Letter.pdf]

Revision 1 – Decision Letter

16-Mar-2021

Dear Dr. Vemuri,

Thank you for your recent submission BRAINCOM-2021-065 entitled "Diffusion models reveal white matter microstructural changes with aging, pathology, and cognition" to Brain Communications.

Your manuscript has been reviewed, and while the reviewers and editorial team appreciate many aspects of the paper, they raise some concerns that you will need to address in order for the paper to be considered for publication.

If you can address these concerns, I invite you to submit a revised manuscript and a response to each of the reviewers' comments. The reviewer(s)' comments are included at the bottom of this message.

In addition to the reviewers' comments, please note our editorial comments added below to comply with our requirements for presentation of scientific data at Brain Communications.

Please format your revised manuscript according to our Instructions to Authors https://academic.oup.com/braincomms/pages/General_Instructions – a summary of key points is attached to help with this.

To revise your manuscript, click the link below or log into https://mc.manuscriptcentral.com/braincom and enter your Author Centre, where you will find your manuscript title listed under "Manuscripts with Decisions." Under "Actions," click on "Create a Revision." Your manuscript number will be appended to denote a revision.

*** PLEASE NOTE: This is a two-step process. After clicking on the link, you will be directed to a webpage to confirm. ***

https://mc.manuscriptcentral.com/braincom?URL_MASK=3c15b787295b462897c53c5b10db1bff

Please upload a revised manuscript with changes tracked with track changes or in coloured text.

When submitting your revised manuscript, you will be asked to respond to the reviewer(s) comments. Please be as specific as possible in your response to all of the comments.

The editorial team at Brain Communications aims to increase transparency in scientific publishing and to demonstrate the value that peer review brings to the scientific process. Please therefore consider whether you are willing to have reviewer(s)' comments on your paper and your response published as supplementary data with the paper if it is accepted. This is entirely optional and will only be done if both the authors and reviewers agree. While submitting your revision there will be an opportunity to review or update the answer you gave previously, if necessary.

IMPORTANT: Your original files are available to you when you upload your revised manuscript. Please delete any redundant files before completing the submission.

Because we are trying to facilitate timely publication of manuscripts submitted to Brain Communications, we would appreciate your revised manuscript as soon as possible. If it is not possible for you to resubmit within 3 months, the option to submit a revision will expire, and we would then consider your revised paper as a new submission. However, if you need a little more time, please get in touch and we can extend this deadline for you.

Once again, thank you for submitting your manuscript to Brain Communications and I look forward to receiving your revision.

Yours sincerely,

Dr. Graciela Muniz Terrera
Editor, Brain Communications

Reviewer(s)' Comments to Author:

Reviewer: 1

Comments to the Author

Review BRAINCOM-2021-065:
Diffusion models reveal white matter microstructural changes with aging, pathology, and cognition

The multimodal neuroimaging study investigates microstructural alterations due to demographics, common age-related pathological processes, and cognition by advanced diffusion MRI analysis, i.e., Neurite Orientation Dispersion and Density Imaging (NODDI). Results were supplemented by amyloid and tau PET scans. The results is that all diffusion measures provided information about WM aging and WM disease processes and were associated with cognition. NODDI and DTI are two different biophysical models that provide distinct information about variation in WM microstructural integrity. The authors conclude that these findings suggest that traditional FA and MD measures perform well in detecting disease-specific pathologies and cognitive effects and claim that NODDI provides additional information which may aid in providing mechanistic insights into disease progression.
This is a very good study, the topic is up to date, analysis is straightforward and well performed, statistics are sound.

Major concern:
1. The study includes a high number of subjects, however, the number of subjects with dementia (N=7) is far too low for any valid statement.
The authors should increase the number of dementia patients substantially (about 50 or a number based on effect size calculations) or omit this disease group from their analysis. Then, they have to tone down their statements regarding the mental development.
2. In addition, what do the authors mean by „disease-specific pathologies‟ in their conclusions. They have only investigated healthy and MCI subjects (and an insufficiently sized dementia group). Please rephrase / clarify.

Minor:
Figure 1: The demographic data of the „representative subject‟ should be given.
Figure 2 would benefit from an abbreviation legend as included in Figure 3.
The manuscript is apparently over-referenced.

Reviewer: 2

Comments to the Author
This is a well-written article investigating NODDI-derived white matter microstructural changes related to age, AD and CVD. The authors provide a thorough assessment of MRI, PET and cognitive metrics and the methods are appropriate and clearly explained. I have a few minor comments which may improve the manuscript slightly:
- A bit more detail could be provided about the participants, particularly the diagnosis of MCI and dementia. Were the diagnoses provided at the same time as the imaging assessment? If they were separate visits, what was the time interval? Both MCI and dementia are pretty heterogeneous conditions. Were there any post-mortem confirmations of particular diagnoses of e.g. AD? Were the MCI and dementia participants tau and/or amyloid positive?
- Is it possible to include additional columns for Table 1, separating out the demographic characteristics into MCI and dementia too rather than just the group as a whole?
- Participants scanned on one of two PRISMA scanners. Were they balanced across the groups? Even though the same parameters were selected it is still always important to check that there aren't any systematic differences between the groups which could be explained by individual scanners.
- The number of dementia patients is very small – I think this should be acknowledged as a limitation in that section of the discussion.
- There is a typo on p9 'NOODDI' should be 'NODDI'

Editorial Office comments:

-Please supply a graphical abstract. A graphical abstract is a visual representation of the central finding or methodology of the paper. It will be displayed in the table of contents. Authors should strive to make the graphical abstract informative, interesting, visually appealing, and straightforward (no caption needed, no abbreviations except gene names). Please upload as a separate high resolution image file (tiff preferred). The technical requirements for the graphical abstracts are:
  --size of the submitted image: 1200 pixels square at 300dpi
  --font of the text: Arial 12-16 point (smaller fonts will not be legible online)

-Please add an Abbreviated Summary (up to 50 words) of your paper that captures the main purpose and conclusions of the work. The summary should state the main results written in the third person ('[1st author surname] et al. report that …') and conclusion. This summary will be used in the contents list online and should not contain any abbreviations other than those on the accepted abbreviations list and accepted gene/protein names, making it accessible to the non-specialist. Please upload it as a Word document. Allowed abbreviations: AIDS; ANOVA; AMPA; ATP; A,T,C,G; CNS; CSF; CT; DNA; ECG; EEG;

EMG; GABA; HIV; MRI; NMDA; PET; PCR; RNA.

-Please include a paragraph about data availability in the 'Materials and Methods' section. We strongly encourage data sharing either as supplementary information or uploads to repositories which you can link in the paper.
