# Supplementary material for: Diffusion models reveal white matter microstructural changes with ageing, pathology and cognition
Source: Brain Commun. 2021 May 19;3(2):fcab106. doi: 10.1093/braincomms/fcab106 (PMC8202149; doi:10.1093/braincomms/fcab106)
Supplement: fcab106_Supplementary_Data [file Revision_1_Decision_Letter.pdf › Revision_1_Decision_Letter.pdf]

Revision 1 – Decision Letter

13-Apr-2021

Dear Dr. Vemuri,

It is a pleasure to accept your manuscript entitled "Diffusion models reveal white matter microstructural changes with aging, pathology, and cognition" for publication in Brain Communications. Any comments made by the reviewer(s) are included below.

The next step is for your paper to go through “First Look”. We will contact you again shortly if any final files or formatting changes are required for publication or if we have any queries.

If no changes are needed, your submission will be transferred to our Production Department and you will hear from the Production Editor in due course.

Video abstracts - Brain Communications encourages authors to provide a video abstract to accompany their article. These are hosted on the Brain/Brain Communications YouTube channel. If you are interested in producing a 5-min video abstract to promote your article, then please contact the Editorial Office (brcoms.editorialoffice@oup.com) for further details and guidelines.

Thank you very much for your contribution. On behalf of the Editors of Brain Communications, we look forward to your future contributions to the Journal.

Yours sincerely,

Graciela Muniz-Terrera
Senior Lecturer in Biostatistics and Epidemiology
Centre Dementia Prevention
University of Edinburgh
UK

Reviewer(s)' Comments to Author:

Reviewer: 1

Comments to the Author
The authors replied appropriately to all my comments.

Reviewer: 2

Comments to the Author
The authors have done an excellent job of addressing all reviewers comments and this is a well-written sound paper.
